# Integrated Network Pharmacology and Proteomic Analyses of Targets and Mechanisms of Jianpi Tianjing Decoction in Treating Vascular Dementia

**DOI:** 10.1155/2023/9021546

**Published:** 2023-01-18

**Authors:** Jidan Liu, Juanfen Gong, Jinchao Xu, Mengyuan Fang, Meng Su, Weiguang Li, Yiyi Wu, Yang Hui, Yingchun He

**Affiliations:** ^1^Department of Geriatrics, Hangzhou TCM Hospital Affiliated to Zhejiang Chinese Medical University, Hangzhou 310000, Zhejiang, China; ^2^Department of Sports Medicine, The Second Affiliated Hospital of Fujian University of Traditional Chinese Medicine, Fuzhou 350000, Fujian, China; ^3^Department of Intensive Care Unit, Hangzhou TCM Hospital Affiliated to Zhejiang Chinese Medical University, Hangzhou 310000, Zhejiang, China; ^4^Department of Emergency, Hangzhou TCM Hospital Affiliated to Zhejiang Chinese Medical University, Hangzhou 310000, Zhejiang, China

## Abstract

**Background:**

Vascular dementia (VD), associated with cerebrovascular injury, is characterized by severe cognitive impairment. Jianpi Tianjing Decoction (JTD) has been widely used to treat VD. However, its molecular targets and mechanisms of action in this treatment remain unclear. This study integrated network pharmacology and proteomics to identify targets and mechanisms of JTD in the treatment of VD and to provide new insights and goals for clinical treatments.

**Methods:**

Systematic network pharmacology was used to identify active chemical compositions, potential targets, and mechanisms of JTD in VD treatment. Then, a mouse model of VD was induced via transient bilateral common carotid artery occlusion to verify the identified targets and mechanisms of JTD against VD using 4D label-free quantitative proteomics.

**Results:**

By screening active chemical compositions and potential targets in relevant databases, 187 active chemical compositions and 416 disease-related compound targets were identified. *In vivo* experiments showed that JTD improved learning and memory in mice. Proteomics also identified 112 differentially expressed proteins in the model and sham groups and the JTD and model groups. Integrating the network pharmacology and proteomics results revealed that JTD may regulate expressions of cytochrome c oxidase subunit 7C, metabotropic glutamate receptor 2, Slc30a1 zinc transporter 1, and apolipoprotein A-IV in VD mice and that their mechanisms involve biological processes like oxidative phosphorylation, regulation of neuron death, glutamate secretion, cellular ion homeostasis, and lipoprotein metabolism.

**Conclusions:**

JTD may suppress VD development via multiple components, targets, and pathways. It may thus serve as a complementary treatment option for patients with VD.

## 1. Introduction

Vascular dementia (VD) is a cognitive dysfunction associated with cerebrovascular injury and characterized by severe impairment of cognitive functions, including attention, memory, verbal fluency, and executive function [1]. Epidemiological studies indicate that VD is the second leading cause of dementia and that in addition to economic burdens, it negatively impacts patient health, productivity, and daily activities [2]. Current studies identify VD as a cognitive dysfunction with multifactorial pathogenesis, probably related to atherosclerosis, lipid metabolism, oxidative stress, the inflammatory response, calcium overload, excitotoxicity, and/or hemostatic activation [3–6]. Drugs that may improve VD symptoms include choline esterase inhibitors (donepezil, galantamine, and rivastigmine) and N-methyl-D-aspartate receptor antagonists (memantine) [7]. Studies have shown that donepezil and galantamine treatments modestly improve cognition but have no effect on activities of daily living [8, 9]. In a randomized controlled trial, incidences of adverse events from donepezil (10 mg, 5 mg) and placebo were 16.3%, 10.1%, and 8.8%, respectively [10]. Another clinical trial showed that compared with a placebo, there were more deaths in the donepezil group [11]. In a trial testing galantamine treatment for VD, the incidence of adverse events from the drug and placebo were 13% and 6%, respectively [12]. Rivastigmine has minimal effects on cognitive symptoms [13]. Memantine produces small benefits in patients with mild to moderate vascular dementia, and current data are insufficient to support the widespread use of memantine in vascular dementia [14]. These drugs also have limited efficacy and adverse effects that include nausea, vomiting, diarrhea, dizziness, headache, and hypertension [14, 15]. Therefore, there is an urgent need to develop complementary and alternative VD therapies.

Chinese herbal formula (CHF) has multiple targets and few side effects, playing an active role in VD treatment [16]. CHF showed fewer adverse effects, lower costs, and improved suitability for long-term use compared with currently prescribed drugs [17]. For example, clinical trials have confirmed that the Shenmayizhi formula combined with ginkgo extract tablets effectively improves cognitive function in mild-to-moderate VD without adverse effects, and clinical outcomes from Dingzhi Yicong granules are superior to those with piracetam in patients with VD [18, 19]. Our team developed Jianpi Tianjing Decoction (JTD) based on years of clinical experience and guided by traditional Chinese medicine (TCM) theory. JTD consists of seven Chinese herbal medicines (CHM), including *Panax ginseng C.A. Meyer*, *Gastrodia elata*, *Atractylodes macrocephala*, *Morinda officinalis Radix*, *Acorus tatarinowii Schott*, *Rhizoma coptidis*, and *Semen cuscutae*. Previous studies have shown that *Gastrodia elata* ameliorates vessel elasticity and prevents atherosclerosis [20]. *Panax ginseng C.A. Meyer* extract attenuates neuronal injury and cognitive deficits in a VD rat model by upregulating the apoptosis regulator Bcl-2 and downregulating the apoptosis regulator BAX (Bax) protein expression [21]. *Rhizoma coptidis* improves the rat neurological function after acute brain injury by increasing the hippocampal brain-derived neurotrophic factor expression [22]. Our preliminary study confirmed that JTD significantly improves cognitive function and quality of life in patients with mild cognitive dysfunction [23, 24]. Animal studies have also identified potential mechanisms of JTD for treating VD, including reducing oxidative stress damage, maintaining hippocampal mitochondrial membrane potential and adenosine triphosphate (ATP) levels, and improving mitochondrial dysfunction [25, 26]. However, CHF composition is so intricate that it is difficult to fully clarify its mechanisms through traditional research methods. Therefore, it is necessary to focus on the potential system-level mechanisms of JTD in VD treatment.

Network pharmacology is a novel method for studying CHM and CHF that combines systematic network analysis and pharmacology to identify interactions among compounds, genes, proteins, and diseases [27]. Tian et al. successfully predicted 28 potentially active Shenzhi Jiannao prescription ingredients and 21 VD therapy targets. They found that the potential targets of these 28 active ingredients mainly involve neuroactive ligand-receptor interactions, calcium, apoptosis, and cholinergic synaptic signaling pathways [28]. Through network pharmacology analysis, Shi et al. discovered that the five core compounds in Yizhi Tongmai decoction exert antiVD effects [29]. Proteomics, an important tool for exploring drug targets and molecular mechanisms, is now also widely applied in many life sciences [30]. It can be used to analyze differentially expressed proteins (DEPs) to explore CHM molecular mechanisms of action. Yang et al. identified 245 Fugui Wenyang Decoction (FGWYD) genes and 145 VD genes via network pharmacology, showing that the Nrf2/HO-1 pathway plays an important role in the FGWYD treatment of VD [31]. That group also used proteomics to verify the neuroprotective mechanistic role of the Nrf2/HO-1 pathway in the FGWYD treatment of VD. An integrated network pharmacology and proteomics analysis can provide a more comprehensive description of CHF molecular mechanisms. Hence, this study integrated network pharmacology and proteomics to analyze the molecular targets and mechanisms of action of JTD in the treatment of VD. First, network pharmacology was performed to predict the target proteins and pathways related to JTD in the treatment of VD. Second, mass spectrometry (MS) analysis was used to identify differentially expressed proteins after VD model mice were treated with JTD. Finally, we revealed the targets and mechanisms of JTD by combining the network pharmacology and proteomic results.

## 2. Materials and Methods

### 2.1. Network Pharmacology Analysis

#### 2.1.1. Data Sources

The TCM systems pharmacology (TCMSP) database (https://www.old.tcmsp-e.com/tcmsp.php (accessed October 7, 2022)), BATMAN-TCM (http://www.bionet.ncpsb.org/batman-tcm/(accessed October 7, 2022)), ETCM (http://www.tcmip.cn/ETCM/(accessed October 7, 2022)), chemical database (http://www.organchem.csdb.cn (accessed October 7, 2022)), and more recent research literature were used to collect the chemical compositions of *Panax ginseng C.A. Meyer*, *Gastrodia elata*, *Atractylodes macrocephala*, *Morindae Officinalis Radix*, *Acorus tatarinowii Schott*, *Rhizoma coptidis*, *and Semen Cuscutae*. Next, active chemical compositions were screened using the Swiss ADME database (http://www.swissadme.ch/(accessed October 9, 2022)) and selected based on oral bioavailability “> 30%,” gastrointestinal absorption level “high,” and “Yes” for atleast three of Lipinski, Ghose, Veber, Egan, or Muegge drug-likeness items or drug-likeness “>0.18.”

#### 2.1.2. Targets of Disease-Related Compounds

VD genes were collected from GeneCards (https://www.genecards.org/(accessed October 10, 2022)), OMIM (https://www.omim.org/ (accessed October 10, 2022)) and Drugbank (https://www.go.drugbank.com/ (accessed October 10, 2022)) databases. The protein targets of active chemical compositions were obtained through the TCMSP and Swiss Target Prediction databases (http://www.swisstargetprediction.ch/(accessed October 10, 2022)). Target protein names were transformed into their equivalent official gene names using the UniProt database (https://www.sparql.uniprot.org/ (accessed October 10, 2022)). An online data analysis and visualization platform (http://www.bioinformatics.com.cn/(accessed October 10, 2022)) was used to plot Venn diagrams and access disease-related compound targets.

#### 2.1.3. Network Construction and Analysis

Cytoscape 3.9.1 software was used to construct the “herb-component-target” network diagram. Disease-related compound targets were imported into the String database (https://www.string-db.org/ (accessed October 11, 2022)), with species “Homo sapiens”, and the protein–protein interaction (PPI) network was generated based on a confidence level ≥0.7. The results were imported into Cytoscape 3.9.1 for visualization and analysis, and the top 30 hub genes were ranked with the CytoHubba plug-in of Cytoscape 3.9.1. Finally, Gene Ontology (GO) and Kyoto Encyclopedia of Genes and Genomes (KEGG) pathway enrichment analyses of potential targets were performed on the Metascape platform (https://www.metascape.org/ (accessed October 11, 2022)).

### 2.2. Animal Experiment

#### 2.2.1. Experimental Animals

Forty Institute of Cancer Research mice (6–8 weeks old males, 20 ± 2 g, specific pathogen-free) were purchased from the Shanghai Institute of Planned Parenthood Research Center for Animal Research (laboratory animal certificate: SCXK (Shanghai) 2018-0006). Animal experiments were approved by the Animal Experimentation Ethics Committee of Zhejiang Chinese Medical University (grant number: IACUC-20210906-12). The mice were kept at the Zhejiang Chinese Medical University Laboratory Animal Research Center (license number: SYXK (Zhejiang) 2021-0012). All mice were allowed to acclimatize for one week prior to being used in experiments. Mice were group housed (five per cage) at 22 ± 2°C and humidity 63 ± 2%, with noise level <55 dB and an alternating 12-h light/dark cycle. The mice had free access to standard laboratory food and tap water.

#### 2.2.2. Experimental Drugs

JTD granules were composed of *Panax ginseng C.A. Meyer* 9 g, *Gastrodia elata* 9 g, *Atractylodes macrocephala* 10 g, *Morindae Officinalis Radix* 6 g, *Acorus tatarinowii Schott* 6 g, *Rhizoma coptidis* 3 g, and *Semen Cuscutae* 12 g. The JTD granules are prepared in accordance with the previous methods [32, 33]. The collected CHM was washed with water to remove any dust or foreign particles present on them and shade-dried for one week at room temperature to avoid excessive loss of volatile components. After drying, the CHM was ground to prepare the crude powder. The above crude powder was subjected to extraction using a hydroalcoholic (30 :  70, water: ethanol) solvent to obtain the CHM granules [34]. All components were supplied by the Hangzhou Hospital of Traditional Chinese Medicine, which is affiliated with Zhejiang Chinese Medical University (*Panax ginseng C.A. Meyer* lot no. 21021463, *Gastrodia elata* lot no. 21041643, *Atractylodes macrocephala* lot no. 21073513, *Morindae Officinalis Radix* lot no. 2101010, *Acorus tatarinowii Schott* lot no. 2104010, *Rhizoma coptidis* lot no. 21051343, and *Semen Cuscutae* lot no. 20111003). Previous studies have shown that the optimal therapeutic dose of JTD in mice is 20.160 g/kg/d [25, 26] and that the typical daily intragastric dose in mice is 10 mg/kg [35]. Thus, we dissolve JTD granules in an appropriate amount of normal saline to achieve a final solution concentration of 2.016 g/ml.

#### 2.2.3. Animal Modeling, Grouping, and Intervention

The 40 mice were randomly divided into three groups: sham surgery (*n* = 10), model (*n* = 15), and JTD (*n* = 15). The transient bilateral common carotid artery occlusion (BCCAO) surgery was performed as previously described with minor modifications [36]. The mice in each group were anesthetized by intraperitoneal injection of 0.3% pentobarbital sodium solution (0.25 ml/10 g). Mice in the model and JTD groups had a midline cervical incision. After exposure, both the right and left common carotid arteries were isolated from the adjacent vagus nerve, and silk was passed below each carotid artery for closure. The bilateral carotid arteries were locked by silk strings for 10 min and then released for 10 min, and this was repeated three times. The strings were then removed and the incision sutured. In the sham group, the same neck region was surgically opened to isolate the vagus nerve and then sutured without a transaction. To prevent wound infection, each mouse received an intramuscular injection of penicillin at a rate of 5,000 units per day for three days. Seven days after surgery, mice in the JTD group were treated with daily intragastric 10 ml/kg/d JTD solution for 28 days. Sham and model groups were treated on the same schedule with saline.

#### 2.2.4. Morris Water Maze Test

After the 28 treatment days, learning and memory were assessed by the Morris water maze test. This test uses a circular pool (100 cm in diameter) with a circular escape platform (6 cm in diameter, 1.0 cm below the water's surface) and an image acquisition system. The pool is divided into four quadrants, with the circular escape platform in the third quadrant. Powdered milk is added to make the water opaque. The mice were released from the four quadrants, respectively, and given 90 s (max) to find the platform. If the mice could not find the platform in 90 s, they were guided onto the platform and allowed to remain for 30 s. Training occurred on four consecutive days. At the end of this training period, the mice were randomly released into the first, second, or fourth quadrant, and their time to reach the platform, or escape latency (EL), was recorded. Testing lasted five days. On day 6, the circular escape platform was removed, and each mouse was placed in the first quadrant. Duration spent in the third quadrant and number of times crossing the platform (TCP) during 1 min were recorded.

### 2.3. Proteomics Methods

#### 2.3.1. Sample Preparation and Protein Extraction

Following Morris water maze testing, all mice were sacrificed, and the extracted brains were immediately placed in liquid nitrogen and stored at −80°C. Three samples from each group were randomly assigned to subsequent analysis. SDT buffer (P0015F, Beyotime, 4% SDS, 100 mM Tris-HCl, pH = 7.6) was added to samples to extract proteins. The supernatant was quantified with the BCA Protein Assay Kit (P0012, Beyotime). Proteins were then digested using the filter-aided sample preparation procedure [37]. The C18 column (IonOpticks, Australia; 25 cm × 75 *μ*m, 1.6 *μ*m C18 beads) was used to desalt the peptide segment.

#### 2.3.2. MS Analysis

Samples were analyzed on a nanoElute (Bruker, Bremen, Germany) coupled to a TIMS TOF Pro (Bruker, Bremen, Germany) equipped with a CaptiveSpray source. Peptides were separated on a 25 cm × 75 *μ*m analytical column, 1.6 *μ*m C18 beads with a packed emitter tip (IonOpticks, Australia). The column was equilibrated using 4 column volumes before loading a sample in 100% buffer A (0.1% formic acid). Samples were separated at 300 nl/min using a linear gradient as follows: 2–22% buffer B (99.9% acetonitrile and 0.1% FA) for 75 min, 22–37% buffer B for 5 min, 37–80% buffer B for 5 min, and hold in 80% buffer B for 5 min. The TIMS TOF Pro was operated in parallel accumulation-serial fragmentation (PASEF) mode. Specifications were as follows: mass range 100–1700 m/z; 1/K0 start 0.75 V·s/cm^2^; end 1.4 V·s/cm^2^; ramp time 100 ms; lock duty cycle to 100%; capillary voltage 1500 V; dry gas 3 L/min; dry temp 180°C. PASEF settings were as follows: 10 MS/MS scans (total cycle time 1.16 sec); charge range 0–5, active exclusion for 0.5 min; scheduling target intensity 10,000; intensity threshold 2,500; and CID collision energy 20–59 eV.

#### 2.3.3. DEP Bioinformatics Analysis

MS data were analyzed using MaxQuant software version 1.6.17.0. A label-free quantitation strategy [38] was used for protein quantitation. Proteins with a fold change >1.2 (or <0.8), and *p* < 0.05 were considered DEPs. Hierarchical cluster analysis was performed using Matplotlib 3.5.1. GO annotations were performed on the DEPs using Blast2GO. The KEGG database (http://www.kegg.jp/) was used to obtain information on the biological pathways of DEPs. GO and KEGG pathway enrichment analyses were evaluated using Fisher's exact probability test. The DEPs were imported into the String database with the species “MusMusculus”, and results were imported into Cytoscape 3.9.1 for visual analysis.

### 2.4. Statistical Analysis

All data were analyzed using IBM SPSS Statistics for Windows, Version 25.0 (Armonk, NY: IBM Corp). Continuous data are expressed as mean ± standard deviation. Significant between-group differences are represented by ^*∗*^*p* < 0.05 or ^*∗∗*^*p* < 0.01. Normality was tested by the Shapiro–Wilk test. Homogeneity of variance was tested by the Levene test. A one-way analysis of variance (ANOVA) was used when variance was homogeneous. For samples with unequal variance, the Mann–Whitney test was used.

**Figure 1:**
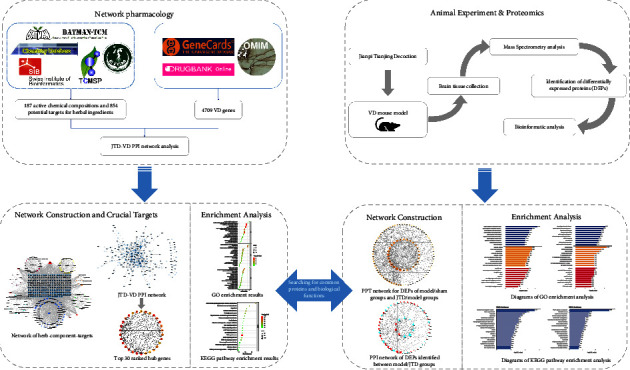
Research protocol.

In addition, the study protocol is shown in Figure 1.

## 3. Results

### 3.1. Targets of Disease-Related Compounds

**Figure 2:**
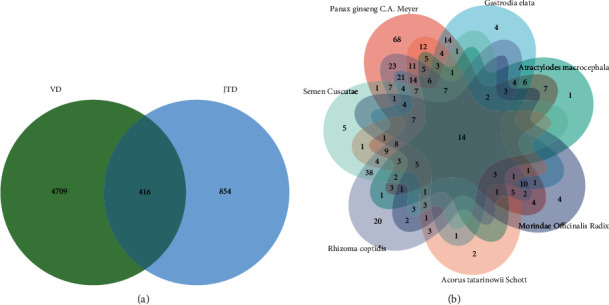
(a) Venn diagram of potential targets for herbal ingredients and VD genes. (b) Venn diagram of intersection targets for each JTD herb.

A total of 187 active chemical compositions in JTD and 854 potential targets for its herbal ingredients were screened from various databases and published literature. A total of 4,709 VD genes were obtained from the GeneCards, OMIM, and DrugBank databases. The 416 disease-related compound targets were obtained by a Venn diagram (Figure 2(a)). Among those disease-related compound targets, *Panax ginseng C.A. Meyer* had 322 potential targets, *Gastrodia elata* had 143 potential targets, *Atractylodes macrocephala* had 84 potential targets, *Morindae Officinalis Radix* had 88 potential targets, *Acorus tatarinowii Schott* had 149 potential targets, *Rhizoma coptidis* had 269 potential targets, and *Semen Cuscutae* had 139 potential targets (Figure 2(b)).

### 3.2. Construction of the JTD-VD PPI Network and Crucial Targets

**Figure 3:**
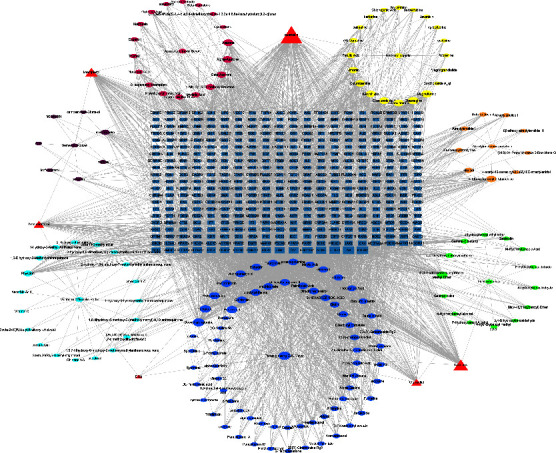
Network of herb-component-targets. Rectangles represent VD genes; ellipses and triangles represent active chemical compositions.

**Figure 4:**
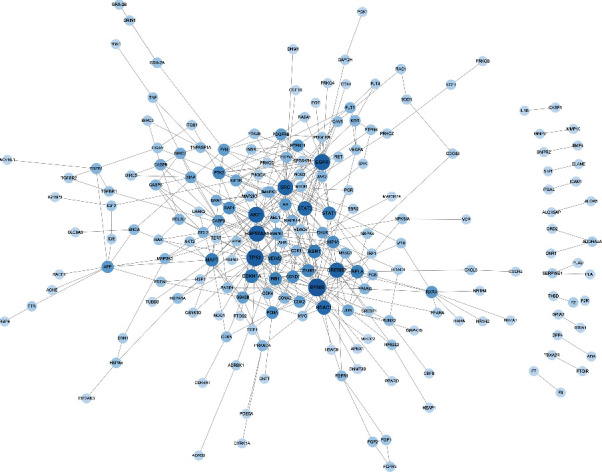
JTD-VD PPI network. Larger degrees are indicated by bigger nodes; darker colors indicate more important nodes.

**Figure 5:**
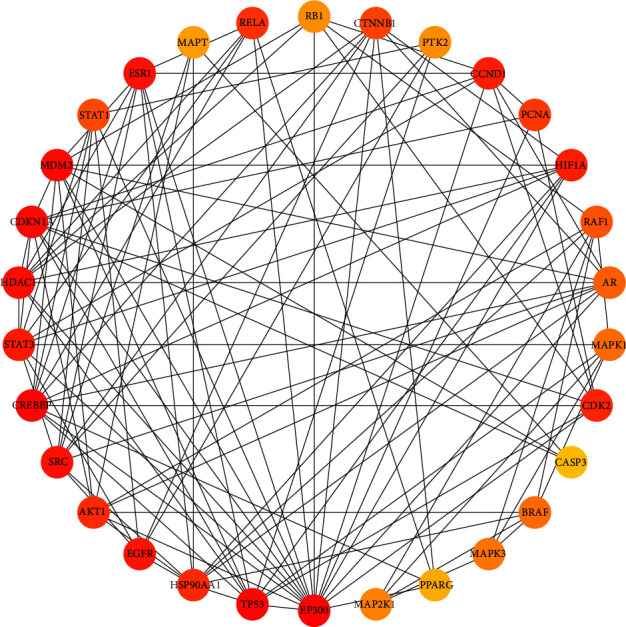
Top 30 ranked hub genes among potential targets. Darker colors indicate more important nodes.

The active chemical compositions and disease-related compound targets were imported into Cytoscape 3.9.1 to construct the herb-component-target network diagram (Figure 3), which consists of 3,671 edges and 585 nodes. The higher the degree value, the larger the node. The top five degrees among all active chemical compositions were quercetin, dauricine, kaempferol, deoxyharringtonine, and panaxacol. Next, the 416 disease-related compound targets were imported into the String database to build the JTD-VD PPI network (Figure 4). The top 30 Hub genes were selected and mapped using the CytoHubba plug-in of Cytoscape 3.9.1, and the top-ranked genes were RAC-alpha serine/threonine-protein kinase, cellular tumor antigen p53, CREB-binding protein, ethylene-responsive transcription factor ESR1, and cyclin-dependent kinase inhibitor 1 (Figure 5).

### 3.3. GO and KEGG Pathways Enrichment Analysis of Potential Targets

**Figure 6:**
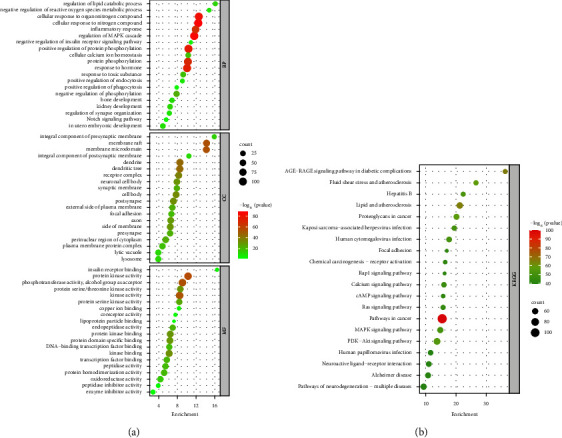
Bubble diagram of GO and KEGG pathway enrichment results. (a) GO enrichment results. (b) KEGG pathway enrichment results. Letters at left of graphs are names of GO terms/KEGG pathways; spot sizes represent gene numbers.

To investigate potential signaling pathways or biological processes (BPs), GO and KEGG pathways were analyzed for potential JTD targets. GO enrichment analysis showed that potential targets were involved in 2,766 GO terms: 3,414 in BP, 234 in cellular components (CC), and 395 in molecular functions (MF). The top 20 GO enrichment analyses are shown in Figure 6(a). GO-BP analysis showed that potential targets focused mainly on the negative regulation of phosphorylation, the inflammatory response, cellular calcium ion homeostasis, regulation of synapse organization, the regulation of the lipid catabolic process, and the cellular response to nitrogen compounds. GO-CC analysis showed that potential targets were primarily focused on the receptor complex, postsynaptic membrane, and presynaptic membrane. Additionally, GO-MF analysis showed that potential targets were concentrated mainly on protease binding, copper ion binding, and lipoprotein particle binding. KEGG enrichment analysis showed that potential targets were involved in 236 pathways (Figure 6(b)). Thus, the mechanisms of action of JTD in the treatment of VD may be closely related to multiple pathways, including lipid and atherosclerosis, neurodegeneration in multiple diseases, calcium signaling, fluid shear stress and atherosclerosis, PI3K-Akt signaling, and MAPK signaling.

### 3.4. General Condition of Experimental Animals and Morris Water Maze Results

No death was observed in the sham group mice, and three mice in each of the JTD and model groups died within three days post-surgery. The cause of death of mice may be accidental death caused by vagus nerve injury caused by excessive traction during operation. Two mice in each of the JTD and model groups died during the 2 weeks. During this period, the cause of death in mice may be due to the stress response, low mood, and inability to eat and drink independently. The rest of the experimental mice survived until the end of the experiment.

**Figure 7:**
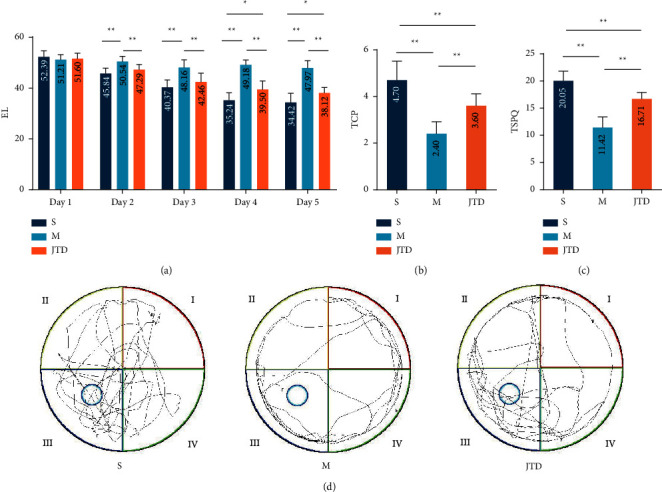
Morris water maze test results. (a) Escape latency (EL) of each mouse group. (b) Number of times for crossing platform (TCP) for each mouse group. (c) Time spent in the platform quadrant (TSPQ) for each mouse group. (d) Representative trajectory plots for each group; S indicates a sham group; M indicates a model group; JTD indicates the Jianpi Tianjing group. ^*∗*^*p* < 0.05; ^*∗∗*^*p* < 0.01.

Morris water maze results are shown in Figures 7(a)–7(d). All groups showed a progressive decrease in EL during training, indicating that mice in each group could learn the platform's location. On trial days 2–5, EL was significantly longer in the model group compared with the sham group and significantly shorter in the JTD group compared with the model group (*p* < 0.01). When the hidden platform was removed on day 6, compared with the sham group, TCP and time spent in the platform quadrant (TSPQ) of the model and JTD groups decreased significantly (*p* < 0.01) and increased significantly in the JTD group compared with the model group (*p* < 0.01).

### 3.5. DEPs Identification

**Figure 8:**
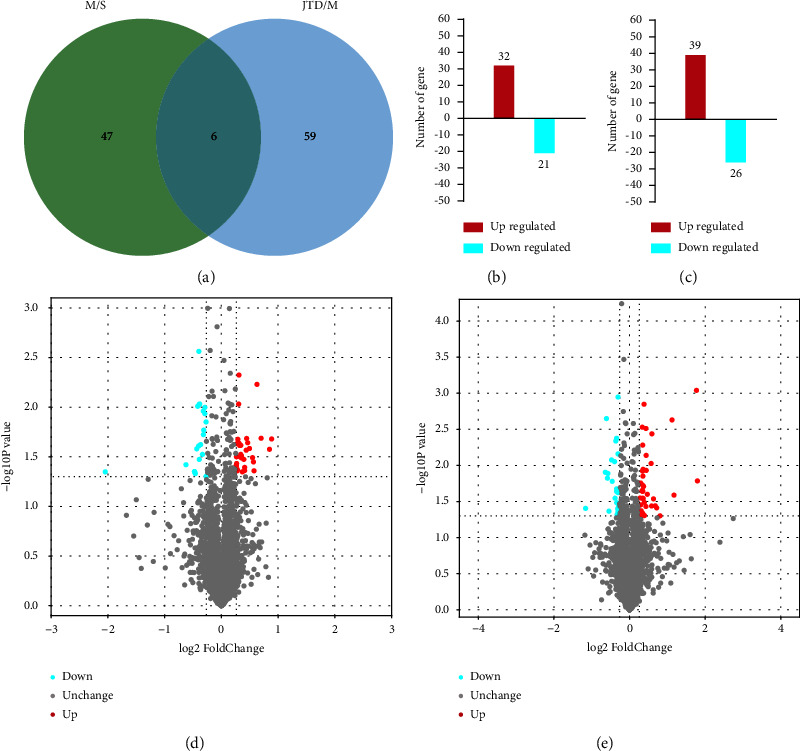
DEP identification results. (a) Venn diagram and overlap. (b) DEPs of model/sham groups. (c) DEPs of JTD/model groups. (d) Volcano plot of model/sham groups. (e) Volcano plot of JTD/model groups. A horizontal coordinate of the volcano plot is the differential expression multiplier (using log2-fold change). The vertical coordinate is *p* value (based on a log transformation of 10, Student's *t*-test). Further horizontal and vertical coordinates from 0 points indicates greater differences. Red represents upregulated proteins. Cyan represents downregulated proteins. Gray and black represent no difference.

**Figure 9:**
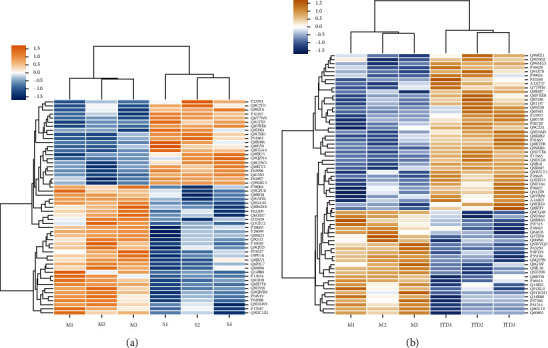
Cluster analysis diagrams. (a) Heat map of model/sham groups. (b) Heat map of model/JTD groups. Horizontal coordinates indicate sample information. Vertical coordinates indicate significant DEPs. Orange represents significantly upregulated proteins. Blue represents significantly downregulated proteins. Gray represents proteins with no quantitative information.

MS analysis identified 5,236 protein groups and 50,097 unique peptides. There were 152 DEPs identified according to the stated criteria (fold-change ratio ≥1.2 or ≤0.833, and *p* < 0.05) (Figures 8(a)–8(e)). Compared with the sham group, there were 32 upregulated proteins and 21 downregulated proteins in the model group (*p* < 0.05). Compared with the model group, there were 39 upregulated proteins and 26 downregulated proteins in the JTD group (*p* < 0.05). The Venn diagram shows DEP overlap (Figure 8(a)). Cluster analysis showed that the up- or downregulated model group proteins showed back regulation in the sham and JTD groups (Figures 9(a) and 9(b)). Alpha-1-antitrypsin 1–3 (serpina1c), potassium voltage-gated channel subfamily C member 2, histone H2A, and protein FAM234B showed upregulation in the model group and downregulation in the JTD group. Adar and Ighg showed downregulation in the model group and upregulation in the JTD group. There were also three overlapping proteins among the DEPs identified by proteomics and the potential targets selected by the network pharmacology: metabotropic glutamate receptor 2 (Grm2), carbonic anhydrase 1, and glycolate oxidase 1.

### 3.6. GO Terms and KEGG Pathway Analysis of DEPs

**Figure 10:**
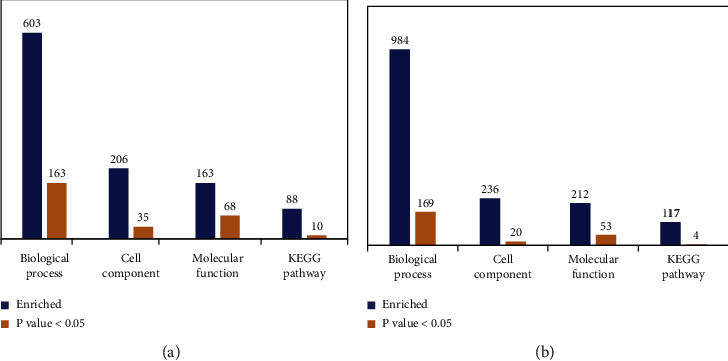
Bioinformatic analysis of DEPs using four analysis categories: BP, CC, MF, and KEGG pathway. Values for each category represent the relations between total protein and DEPs. *p* < 0.05 is statistically significant. (a) Bioinformatic analysis of DEPs identified between model/sham groups. (b) Bioinformatic analysis of DEPs identified between JTD/model groups.

GO enrichment analysis revealed 163 BP terms, 35 CC terms, and 68 MF terms between the model and sham groups; there were 169 BP terms, 20 CC terms, and 53 MF terms between the JTD and model groups (Figure 10).

**Figure 11:**
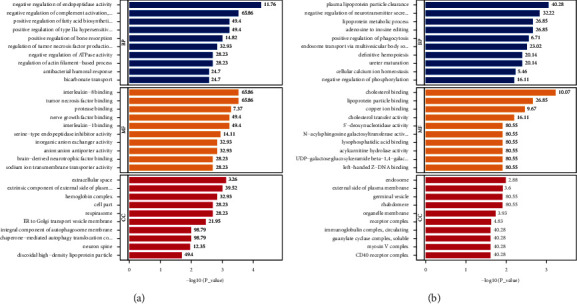
Diagrams of GO enrichment analysis. (a) Top 10 significantly enriched GO terms for DEPs identified between model/sham groups. (b) Top 10 significantly enriched GO terms for DEPs identified between JTD/model groups.

GO analysis of the DEPs of the model and sham groups showed that negative regulation of endopeptidase activity, negative regulation of complement activation, the lectin pathway, positive regulation of the fatty acid biosynthetic process, and negative regulation of ATPase activity were the primary BPs. These DEPs are mainly in the extracellular space, the external plasma membrane, and the hemoglobin complex. They are also associated with receptor binding, ion exchange, and functions (Figure 11(a)).

Next, GO enrichment analysis was performed on the DEPs of the JTD and model groups (Figure 11(b)). GO-BP analysis showed that these DEPs are significantly involved in plasma lipoprotein particle clearance, negative regulation of neurotransmitter secretion, the lipoprotein metabolism process, positive regulation of phagocytosis, definitive hemopoiesis, cellular calcium ion homeostasis, and negative regulation of phosphorylation. GO-CC analysis showed that these DEPs are mainly located in the organelle membrane, receptor complex, and outer side of the plasma membrane. The GO-MF analysis showed they are associated with MFs like cholesterol binding, lipoprotein binding, ion binding, and protease activity.

**Figure 12:**
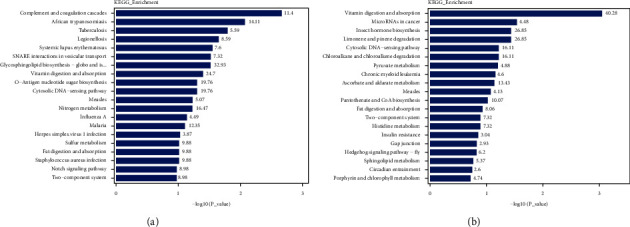
Diagrams of KEGG pathway enrichment analysis. (a) Top 20 significantly enriched KEGG pathways for DEPs identified between model/sham groups. (b) Top 20 significantly enriched KEGG pathways for DEPs identified between JTD/model groups.

KEGG pathway enrichment analysis showed that the top five signaling pathways were complement and coagulation cascades, African trypanosomiasis, tuberculosis, legionellosis, and systemic lupus erythematosus in the DEPs of the model and sham groups (Figure 12(a)). Similarly, the DEPs of the JTD and model groups are mainly involved in vitamin digestion and absorption, fat digestion and absorption, and pyruvate metabolism (Figure 12(b)).

### 3.7. PPI Network

**Figure 13:**
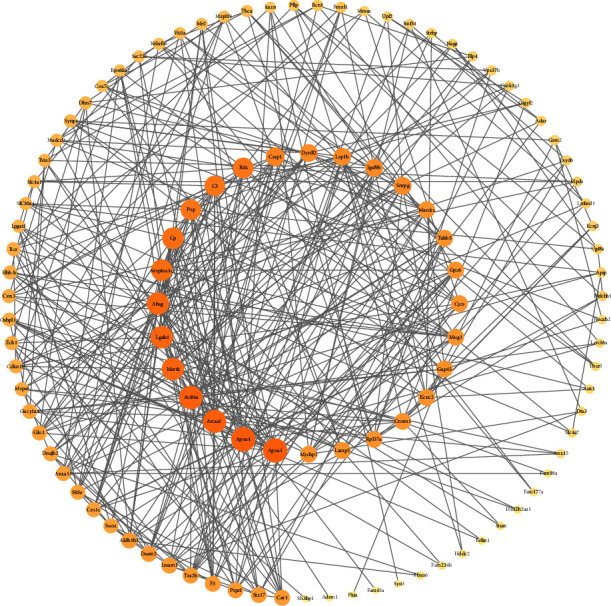
PPT network for DEPs of model/sham groups and JTD/model groups. A larger degree is indicated by a bigger node; darker colors indicate more important nodes.

**Figure 14:**
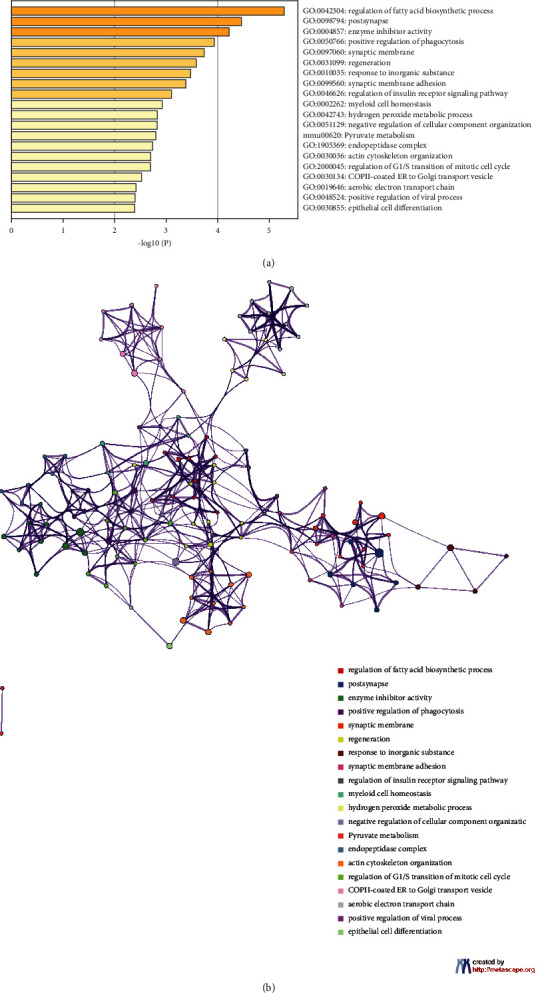
Metascape analysis results. (a) Top 20 DEP enrichment results. (b) PPI network is colored by enrichment results. A circle node represents GO or KEGG terms, whose size is proportional to the number of genes under that term, and nodes of the same color belong to the same cluster.

**Figure 15:**
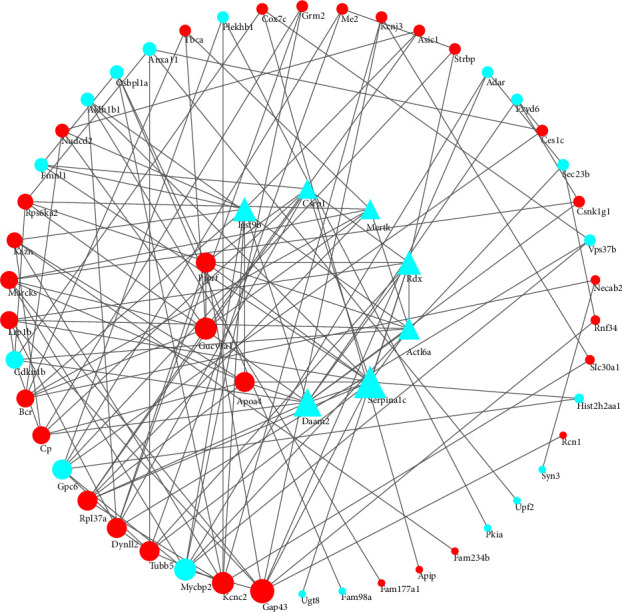
PPI network of DEPs identified between model/JTD groups. Red represents upregulated proteins. Cyan represents downregulated proteins. Triangles represent hub genes filtered by the cytoHubba plug-in of Cytoscape 3.9.1.

DEPs of the model and sham groups and the JTD and model groups were combined and de-duplicated to obtain 112 common DEPs. Next, Cytoscape 3.9.1 was used to construct a PPI network diagram for these DEPs (Figure 13). Metascape analysis of this network included regulation of the fatty acid biosynthetic process, positive regulation of phagocytosis, response to inorganic substance, pyruvate metabolism, and aerobic electron transport chain (Figures 14(a) and 14(b)). Similarly, the DEPs of the JTD and model groups were imported into String to construct a PPI network (Figure 15). Based on the CytoHubba plug-in of Cytoscape 3.9.1, 10 hub genes were screened, including actin-like protein 6A, tyrosine-protein kinase Mer, cysteine and glycine-rich protein 1, protein turtle homolog B, Apolipoprotein A-IV (Apoa4), radixin, disheveled-associated activator of morphogenesis 2, Serpina1c, and guanylate cyclase soluble subunit alpha-1. Grm2, cytochrome c oxidase subunit 7C (Cox7c), and Slc30a1 (also known as zinc transporter 1 (Znt1)) also participated in this PPI network.

## 4. Discussion

VD is a cluster of cognitive disorder syndromes caused by cerebrovascular lesions. Current risk factors for VD include advanced age, diabetes, hypertension, hyperlipidemia, atherosclerosis, and stroke [39]. In particular, VD risk nearly doubles post-stroke [40]. Cerebral hypoperfusion from cerebrovascular disorders may be a potential VD mechanism [41, 42]. Neuropathology studies have reported that cerebral hypoperfusion results in reduced glucose and oxygen supplies, leading to cellular energy metabolism [43], ionic imbalance [44], excitotoxicity [45], oxidative stress [46], and neuroinflammation [47]. These mechanisms drive downstream structural changes, including blood–brain barrier dysfunction, white matter lesions, microinfarcts, and hippocampal atrophy, which may play a direct pathogenic role in VD [48, 49]. VD accounts for ∼15–20% of dementia cases, and its incidence increases dramatically with age [50, 51]. VD both affects patient quality of life and increases the risk of death [52]. Therefore, finding effective treatments remains a research focus. JTD has long been used to effectively treat VD [23, 24]. To further investigate the molecular mechanisms of JTD in VD treatment, this study combined network pharmacology and proteomics data to gain a global overview.

### 4.1. Regulation of Mitochondrial Dysfunction

Mitochondrial dysfunction has also been reported to be a significant factor in VD [43]. Under anoxic conditions, the mitochondrial electron transport chain is disturbed, leading to increased reactive oxygen species (ROS) production [53]. Oxidative stress, which occurs when the ROS-antioxidant balance is disrupted, is increasingly understood to be involved in VD [54]. Under normal conditions, the brain depends on a constant energy supply from ATP through mitochondrial oxidative phosphorylation [55]. However, oxidative stress can cause mitochondrial dysfunction, triggering impaired cerebral energy metabolism and neuronal death [56, 57]. Cyclooxygenases (COX) are the main enzyme in the mitochondrial electron transport chain, which uses oxygen in the generation of ATP via oxidative phosphorylation [58]. The network pharmacology and proteomic analyses herein show that negative regulation of phosphorylation is another important BP. Proteomics identified Cox7c to be involved in oxidative phosphorylation and metabolism pathways. Cox7c, a member of the cytochrome c oxidase complex responsible for mitochondrial respiration promotes ATP synthesis and reduces mitochondrial dysfunction [59]. Recent studies have revealed Cox7c to be a potential biomarker of pathogenesis in Alzheimer's disease [60]. However, the effects of Cox7c in VD require further clarification. Herein, Cox7c expression was upregulated in brain tissue of VD mice treated with JTD, indicating that this CHF may play a role in treating VD by attenuating mitochondrial dysfunction.

### 4.2. Neuronal Glutamate Excitotoxicity Regulation

A main cause of VD-induced cognitive dysfunction is excitotoxicity. Glutamate excitotoxicity has been hypothesized to be excessively activated by excitatory glutamate receptors, causing neuronal dysfunction or death [45]. Grm2 encodes metabotropic glutamate subtype receptor 2, known as mGluR2. Past studies have shown that GRM2 may be involved in regulating neural apoptosis, or cell death caused by hypoxia and ischemia [61]. Herein, Grm2 was an overlap protein between potential JTD targets and DEPs, which is mainly involved in the regulation of neuronal death, glutamate receptor activity, and glutamate secretion. Therefore, JTD may regulate the GRM2 expression and modulate these processes, reducing brain tissue damage and improving cognitive function.

### 4.3. Cellular Ion Homeostasis Regulation

Cerebral ischemia enhances synaptic activity, leading to increased zinc release. However, high intracellular zinc levels may become toxic to neurons and neuroglia [62], rapidly leading to cell death [63]. This may be another contributor to VD. Among the 65 DEPs, Slc30a1 was the only protein that regulates cellular zinc ions and calcium ion homeostatic processes. Slc30a1/Znt1 is well-known as a crucial regulator of zinc absorption and transport [64]. Znt1 reduces glial and neuronal zinc levels, protecting these cells from zinc toxicity and reducing their deaths [65, 66]. The proteomics data herein also confirmed that Znt1 levels are significantly increased in VD mice treated with JTD, further confirming the neuroprotective effects of JTD.

### 4.4. Atherosclerosis Regulation

Herein, the KEGG pathway enrichment analysis revealed that ApoA4 participates in several signaling pathways, including lipid and atherosclerosis, fat digestion and absorption, and cholesterol metabolism. Carotid artery stenosis and atherosclerosis are risk factors for cognitive impairment [67]. It is well accepted that ApoA4 is a major component of high-density lipoprotein and chylomicrons, which have anti-atherosclerotic effects [68]. Exogenous administration of ApoA4 reduces the incidence of acute rupture of arterial plaques in the apolipoprotein E knockout mouse model, confirming its ability to stabilize plaque [69]. In addition, previous research has confirmed that ApoA4 is involved in lipid uptake and metabolism [70] and anti-atherosclerosis [71] and inhibits thrombosis [72]. This is consistent with our results showing that, compared with the model group, ApoA4 expression was upregulated in the JTD group, further confirming its important role in the anti-atherosclerotic effects of JTD.

## 5. Conclusions

Integrated network pharmacology and proteomics analysis revealed that Cox7c, Grm2, Slc30a1, and ApoA4 are critical targets of JTD in VD treatment. *In vivo* mechanisms may be involved in attenuating mitochondrial dysfunction, reducing excitotoxicity, maintaining cellular ion homeostasis and antiatherosclerosis.

## Data Availability

The data used to support the findings of this study are available from the corresponding author upon request.

## Abbreviations

ApoA4: Apolipoprotein A-IV
ATP: Adenosine triphosphate
Bax: Apoptosis regulator BAX
BPs: Biological processes
CC: Cellular components
CHF: Chinese herbal formula
CHM: Chinese herbal medicine
COX: Cyclooxygenases
Cox7c: Cytochrome c oxidase subunit 7C
DEPs: Differentially expressed proteins
EL: Escape latency
FGWYD: Fugui Wenyang Decoction
GO: Gene Ontology
GRM2: Metabotropic glutamate receptor 2
JTD: Jianpi Tianjing Decoction
KEGG: Kyoto Encyclopedia of Genes and Genomes
MF: Molecular functions
MS: Mass spectrometry
PASEF: Parallel accumulation-serial fragmentation
PPI: Protein-protein interaction
ROS: Reactive oxygen species
Serpina1c: Alpha-1-antitrypsin 1–3
TCP: Times crossing platform
TCM: Traditional Chinese medicine
TCMSP: Traditional Chinese medicine systems pharmacology
TSPQ: Time spent in platform quadrant
Znt1: Zinc transporter 1.

## Supplementary Materials

Supplementary Materials Supplementary Table 1: Active JTD chemical compositions and targets. Supplementary Table 2: Morris water maze results. Supplementary Table 3: Differentially expressed proteins (DEPs) identification results.
