# Supplementary material for: Integrated Network Pharmacology and Proteomic Analyses of Targets and Mechanisms of Jianpi Tianjing Decoction in Treating Vascular Dementia
Source: Evid Based Complement Alternat Med. 2023 Jan 18;2023:9021546. doi: 10.1155/2023/9021546 (PMC9876684; doi:10.1155/2023/9021546)
Supplement: Supplementary Materials — Supplementary Table 1: Active JTD chemical compositions and targets. Supplementary Table 2: Morris water maze results. Supplementary Table 3: Differentially expressed proteins (DEPs) identification results. [file 9021546.f1.zip › Table 1 Active JTD chemical compositions and targets.pdf]

Table 1: Network pharmacology data

Herb name	Active Chemical Compositions of JTD	Disease-related compound targets
Acorus tatarinowii Schott	(1R,3aS,4R,6aS)-1,4-bis(3,4-dimethoxyphenyl)-1,3,3a,4,6,6a-hexahydrofuro[4,3-c]furan	CALB2
Acorus tatarinowii Schott	(1R,3aS,4R,6aS)-1,4-bis(3,4-dimethoxyphenyl)-1,3,3a,4,6,6a-hexahydrofuro[4,3-c]furan	F10
Acorus tatarinowii Schott	(1R,3aS,4R,6aS)-1,4-bis(3,4-dimethoxyphenyl)-1,3,3a,4,6,6a-hexahydrofuro[4,3-c]furan	PTGS2
Acorus tatarinowii Schott	1-Allyl-2,4,5-Trimethoxy-Benzene	ADORA1
Acorus tatarinowii Schott	1-Allyl-2,4,5-Trimethoxy-Benzene	ADORA2A
Acorus tatarinowii Schott	1-Allyl-2,4,5-Trimethoxy-Benzene	BDKRB2
Acorus tatarinowii Schott	1-Allyl-2,4,5-Trimethoxy-Benzene	C5AR1
Acorus tatarinowii Schott	1-Allyl-2,4,5-Trimethoxy-Benzene	CASP3
Acorus tatarinowii Schott	1-Allyl-2,4,5-Trimethoxy-Benzene	CDK1
Acorus tatarinowii Schott	1-Allyl-2,4,5-Trimethoxy-Benzene	CDK2
Acorus tatarinowii Schott	1-Allyl-2,4,5-Trimethoxy-Benzene	CHRM1
Acorus tatarinowii Schott	1-Allyl-2,4,5-Trimethoxy-Benzene	CHRM2
Acorus tatarinowii Schott	1-Allyl-2,4,5-Trimethoxy-Benzene	CREBBP
Acorus tatarinowii Schott	1-Allyl-2,4,5-Trimethoxy-Benzene	CSNK1D
Acorus tatarinowii Schott	1-Allyl-2,4,5-Trimethoxy-Benzene	CYP11B2
Acorus tatarinowii Schott	1-Allyl-2,4,5-Trimethoxy-Benzene	CYP3A4
Acorus tatarinowii Schott	1-Allyl-2,4,5-Trimethoxy-Benzene	EP300
Acorus tatarinowii Schott	1-Allyl-2,4,5-Trimethoxy-Benzene	GALR3
Acorus tatarinowii Schott	1-Allyl-2,4,5-Trimethoxy-Benzene	GSK3B
Acorus tatarinowii Schott	1-Allyl-2,4,5-Trimethoxy-Benzene	HMGCR
Acorus tatarinowii Schott	1-Allyl-2,4,5-Trimethoxy-Benzene	HMOX1
Acorus tatarinowii Schott	1-Allyl-2,4,5-Trimethoxy-Benzene	HRH2
Acorus tatarinowii Schott	1-Allyl-2,4,5-Trimethoxy-Benzene	HSD11B1
Acorus tatarinowii Schott	1-Allyl-2,4,5-Trimethoxy-Benzene	HTR2A
Acorus tatarinowii Schott	1-Allyl-2,4,5-Trimethoxy-Benzene	IDO1
Acorus tatarinowii Schott	1-Allyl-2,4,5-Trimethoxy-Benzene	JAK2
Acorus tatarinowii Schott	1-Allyl-2,4,5-Trimethoxy-Benzene	KDR
Acorus tatarinowii Schott	1-Allyl-2,4,5-Trimethoxy-Benzene	MAP3K14
Acorus tatarinowii Schott	1-Allyl-2,4,5-Trimethoxy-Benzene	MAPK10
Acorus tatarinowii Schott	1-Allyl-2,4,5-Trimethoxy-Benzene	MPO
Acorus tatarinowii Schott	1-Allyl-2,4,5-Trimethoxy-Benzene	NOS1
Acorus tatarinowii Schott	1-Allyl-2,4,5-Trimethoxy-Benzene	NOS3
Acorus tatarinowii Schott	1-Allyl-2,4,5-Trimethoxy-Benzene	NQO1
Acorus tatarinowii Schott	1-Allyl-2,4,5-Trimethoxy-Benzene	P2RX7
Acorus tatarinowii Schott	1-Allyl-2,4,5-Trimethoxy-Benzene	PARP1

Acorus tatarinowii Schott	1-Allyl-2,4,5-Trimethoxy-Benzene	PDE10A
Acorus tatarinowii Schott	1-Allyl-2,4,5-Trimethoxy-Benzene	RGS4
Acorus tatarinowii Schott	8-Isopentenyl-kaempferol	AR
Acorus tatarinowii Schott	8-Isopentenyl-kaempferol	CALB2
Acorus tatarinowii Schott	8-Isopentenyl-kaempferol	CCNA2
Acorus tatarinowii Schott	8-Isopentenyl-kaempferol	CDK2
Acorus tatarinowii Schott	8-Isopentenyl-kaempferol	DPP4
Acorus tatarinowii Schott	8-Isopentenyl-kaempferol	ESR2
Acorus tatarinowii Schott	8-Isopentenyl-kaempferol	F10
Acorus tatarinowii Schott	8-Isopentenyl-kaempferol	F2
Acorus tatarinowii Schott	8-Isopentenyl-kaempferol	F7
Acorus tatarinowii Schott	8-Isopentenyl-kaempferol	GSK3B
Acorus tatarinowii Schott	8-Isopentenyl-kaempferol	KCNMA1
Acorus tatarinowii Schott	8-Isopentenyl-kaempferol	KDR
Acorus tatarinowii Schott	8-Isopentenyl-kaempferol	MAPK14
Acorus tatarinowii Schott	8-Isopentenyl-kaempferol	PDE3A
Acorus tatarinowii Schott	8-Isopentenyl-kaempferol	PPARG
Acorus tatarinowii Schott	8-Isopentenyl-kaempferol	PTGS1
Acorus tatarinowii Schott	8-Isopentenyl-kaempferol	PTGS2
Acorus tatarinowii Schott	8-Isopentenyl-kaempferol	RXRA
Acorus tatarinowii Schott	Alpha-Asarone	ABCB1
Acorus tatarinowii Schott	Alpha-Asarone	AHR
Acorus tatarinowii Schott	Alpha-Asarone	APP
Acorus tatarinowii Schott	Alpha-Asarone	BDKRB2
Acorus tatarinowii Schott	Alpha-Asarone	C5AR1
Acorus tatarinowii Schott	Alpha-Asarone	CA1
Acorus tatarinowii Schott	Alpha-Asarone	CASP3
Acorus tatarinowii Schott	Alpha-Asarone	CDK1
Acorus tatarinowii Schott	Alpha-Asarone	CDK2
Acorus tatarinowii Schott	Alpha-Asarone	CETP
Acorus tatarinowii Schott	Alpha-Asarone	CHRM1
Acorus tatarinowii Schott	Alpha-Asarone	CHRM2
Acorus tatarinowii Schott	Alpha-Asarone	CREBBP
Acorus tatarinowii Schott	Alpha-Asarone	CRHR1
Acorus tatarinowii Schott	Alpha-Asarone	CSNK1D
Acorus tatarinowii Schott	Alpha-Asarone	CTSB
Acorus tatarinowii Schott	Alpha-Asarone	CTSF
Acorus tatarinowii Schott	Alpha-Asarone	CTSL
Acorus tatarinowii Schott	Alpha-Asarone	CYP11B2
Acorus tatarinowii Schott	Alpha-Asarone	CYP1A2
Acorus tatarinowii Schott	Alpha-Asarone	EGFR
Acorus tatarinowii Schott	Alpha-Asarone	ELANE
Acorus tatarinowii Schott	Alpha-Asarone	ESR1
Acorus tatarinowii Schott	Alpha-Asarone	GRM2

Acorus tatarinowii Schott	Alpha-Asarone	HMGCR
Acorus tatarinowii Schott	Alpha-Asarone	HRH2
Acorus tatarinowii Schott	Alpha-Asarone	HTR6
Acorus tatarinowii Schott	Alpha-Asarone	IDO1
Acorus tatarinowii Schott	Alpha-Asarone	JAK2
Acorus tatarinowii Schott	Alpha-Asarone	MAOA
Acorus tatarinowii Schott	Alpha-Asarone	MAP3K14
Acorus tatarinowii Schott	Alpha-Asarone	MAPK10
Acorus tatarinowii Schott	Alpha-Asarone	MAPK14
Acorus tatarinowii Schott	Alpha-Asarone	MAPT
Acorus tatarinowii Schott	Alpha-Asarone	MIF
Acorus tatarinowii Schott	Alpha-Asarone	MPO
Acorus tatarinowii Schott	Alpha-Asarone	NOS1
Acorus tatarinowii Schott	Alpha-Asarone	NOS3
Acorus tatarinowii Schott	Alpha-Asarone	NQO1
Acorus tatarinowii Schott	Alpha-Asarone	P2RX7
Acorus tatarinowii Schott	Alpha-Asarone	PARP1
Acorus tatarinowii Schott	Alpha-Asarone	PDE10A
Acorus tatarinowii Schott	Alpha-Asarone	PTGS1
Acorus tatarinowii Schott	Alpha-Asarone	PTGS2
Acorus tatarinowii Schott	Alpha-Asarone	RELA
Acorus tatarinowii Schott	Alpha-Asarone	RGS4
Acorus tatarinowii Schott	Alpha-Asarone	SLC6A3
Acorus tatarinowii Schott	Alpha-Asarone	TUBB3
Acorus tatarinowii Schott	Asaronaldehyde	CA1
Acorus tatarinowii Schott	Asaronaldehyde	ERN1
Acorus tatarinowii Schott	Asaronaldehyde	FAAH
Acorus tatarinowii Schott	Asaronaldehyde	MMP12
Acorus tatarinowii Schott	Asaronaldehyde	NAMPT
Acorus tatarinowii Schott	Asaronaldehyde	TGM2
Acorus tatarinowii Schott	Asaronaldehyde	TYMP
Acorus tatarinowii Schott	Asarone	ABCB1
Acorus tatarinowii Schott	Asarone	AHR
Acorus tatarinowii Schott	Asarone	APP
Acorus tatarinowii Schott	Asarone	BDKRB2
Acorus tatarinowii Schott	Asarone	C5AR1
Acorus tatarinowii Schott	Asarone	CA1
Acorus tatarinowii Schott	Asarone	CASP3
Acorus tatarinowii Schott	Asarone	CDK1
Acorus tatarinowii Schott	Asarone	CDK2
Acorus tatarinowii Schott	Asarone	CETP
Acorus tatarinowii Schott	Asarone	CHRM1
Acorus tatarinowii Schott	Asarone	CHRM2
Acorus tatarinowii Schott	Asarone	CREBBP

Acorus tatarinowii Schott	Asarone	CRHR1
Acorus tatarinowii Schott	Asarone	CSNK1D
Acorus tatarinowii Schott	Asarone	CTSB
Acorus tatarinowii Schott	Asarone	CTSF
Acorus tatarinowii Schott	Asarone	CTSL
Acorus tatarinowii Schott	Asarone	CYP11B2
Acorus tatarinowii Schott	Asarone	CYP1A2
Acorus tatarinowii Schott	Asarone	EGFR
Acorus tatarinowii Schott	Asarone	ELANE
Acorus tatarinowii Schott	Asarone	ESR1
Acorus tatarinowii Schott	Asarone	GRM2
Acorus tatarinowii Schott	Asarone	HMGCR
Acorus tatarinowii Schott	Asarone	HRH2
Acorus tatarinowii Schott	Asarone	HTR6
Acorus tatarinowii Schott	Asarone	IDO1
Acorus tatarinowii Schott	Asarone	JAK2
Acorus tatarinowii Schott	Asarone	MAOA
Acorus tatarinowii Schott	Asarone	MAP3K14
Acorus tatarinowii Schott	Asarone	MAPK10
Acorus tatarinowii Schott	Asarone	MAPK14
Acorus tatarinowii Schott	Asarone	MAPT
Acorus tatarinowii Schott	Asarone	MIF
Acorus tatarinowii Schott	Asarone	MPO
Acorus tatarinowii Schott	Asarone	NOS1
Acorus tatarinowii Schott	Asarone	NOS3
Acorus tatarinowii Schott	Asarone	NQO1
Acorus tatarinowii Schott	Asarone	P2RX7
Acorus tatarinowii Schott	Asarone	PARP1
Acorus tatarinowii Schott	Asarone	PDE10A
Acorus tatarinowii Schott	Asarone	PTGS1
Acorus tatarinowii Schott	Asarone	PTGS2
Acorus tatarinowii Schott	Asarone	RELA
Acorus tatarinowii Schott	Asarone	RGS4
Acorus tatarinowii Schott	Asarone	SLC6A3
Acorus tatarinowii Schott	Asarone	TUBB3
Acorus tatarinowii Schott	Beta-Asarone	ABCB1
Acorus tatarinowii Schott	Beta-Asarone	AHR
Acorus tatarinowii Schott	Beta-Asarone	APP
Acorus tatarinowii Schott	Beta-Asarone	BDKRB2
Acorus tatarinowii Schott	Beta-Asarone	C5AR1
Acorus tatarinowii Schott	Beta-Asarone	CA1
Acorus tatarinowii Schott	Beta-Asarone	CASP3
Acorus tatarinowii Schott	Beta-Asarone	CETP
Acorus tatarinowii Schott	Beta-Asarone	CHRM1

Acorus tatarinowii Schott	Beta-Asarone	CHRM2
Acorus tatarinowii Schott	Beta-Asarone	CRHR1
Acorus tatarinowii Schott	Beta-Asarone	CSNK1D
Acorus tatarinowii Schott	Beta-Asarone	CYP11B2
Acorus tatarinowii Schott	Beta-Asarone	CYP1A2
Acorus tatarinowii Schott	Beta-Asarone	EGFR
Acorus tatarinowii Schott	Beta-Asarone	ELANE
Acorus tatarinowii Schott	Beta-Asarone	ESR1
Acorus tatarinowii Schott	Beta-Asarone	GRM2
Acorus tatarinowii Schott	Beta-Asarone	HMGCR
Acorus tatarinowii Schott	Beta-Asarone	HRH2
Acorus tatarinowii Schott	Beta-Asarone	IDO1
Acorus tatarinowii Schott	Beta-Asarone	JAK2
Acorus tatarinowii Schott	Beta-Asarone	MAOA
Acorus tatarinowii Schott	Beta-Asarone	MAPK10
Acorus tatarinowii Schott	Beta-Asarone	MAPK14
Acorus tatarinowii Schott	Beta-Asarone	MAPT
Acorus tatarinowii Schott	Beta-Asarone	MIF
Acorus tatarinowii Schott	Beta-Asarone	MPO
Acorus tatarinowii Schott	Beta-Asarone	NOS1
Acorus tatarinowii Schott	Beta-Asarone	NOS3
Acorus tatarinowii Schott	Beta-Asarone	NQO1
Acorus tatarinowii Schott	Beta-Asarone	P2RX7
Acorus tatarinowii Schott	Beta-Asarone	PARP1
Acorus tatarinowii Schott	Beta-Asarone	PDE10A
Acorus tatarinowii Schott	Beta-Asarone	PTGS1
Acorus tatarinowii Schott	Beta-Asarone	PTGS2
Acorus tatarinowii Schott	Beta-Asarone	RELA
Acorus tatarinowii Schott	Beta-Asarone	RGS4
Acorus tatarinowii Schott	Beta-Asarone	SLC6A3
Acorus tatarinowii Schott	Beta-Asarone	TUBB3
Acorus tatarinowii Schott	Beta-Humulene	ADORA1
Acorus tatarinowii Schott	Beta-Humulene	ADORA2A
Acorus tatarinowii Schott	Beta-Humulene	CNR2
Acorus tatarinowii Schott	Beta-Humulene	MAOB
Acorus tatarinowii Schott	Beta-Humulene	PPARA
Acorus tatarinowii Schott	Cycloartenol	NR3C2
Acorus tatarinowii Schott	Eugenol	ADORA1
Acorus tatarinowii Schott	Eugenol	ADORA2A
Acorus tatarinowii Schott	Eugenol	ALPL
Acorus tatarinowii Schott	Eugenol	AR
Acorus tatarinowii Schott	Eugenol	CHRNA4
Acorus tatarinowii Schott	Eugenol	CXCR2
Acorus tatarinowii Schott	Eugenol	DAO

Acorus tatarinowii Schott	Eugenol	HDAC6
Acorus tatarinowii Schott	Eugenol	KCNMA1
Acorus tatarinowii Schott	Eugenol	KDR
Acorus tatarinowii Schott	Eugenol	PARP1
Acorus tatarinowii Schott	Eugenol	PTGS1
Acorus tatarinowii Schott	Eugenol	SRC
Acorus tatarinowii Schott	Eugenol	VEGFA
Acorus tatarinowii Schott	Eugenol Methyl Ether	ACHE
Acorus tatarinowii Schott	Eugenol Methyl Ether	ADORA2A
Acorus tatarinowii Schott	Eugenol Methyl Ether	AR
Acorus tatarinowii Schott	Eugenol Methyl Ether	CHRM1
Acorus tatarinowii Schott	Eugenol Methyl Ether	CHRM2
Acorus tatarinowii Schott	Eugenol Methyl Ether	CREBBP
Acorus tatarinowii Schott	Eugenol Methyl Ether	CYP11B2
Acorus tatarinowii Schott	Eugenol Methyl Ether	EP300
Acorus tatarinowii Schott	Eugenol Methyl Ether	GALR3
Acorus tatarinowii Schott	Eugenol Methyl Ether	GRM2
Acorus tatarinowii Schott	Eugenol Methyl Ether	HMGCR
Acorus tatarinowii Schott	Eugenol Methyl Ether	HMOX1
Acorus tatarinowii Schott	Eugenol Methyl Ether	HTR2A
Acorus tatarinowii Schott	Eugenol Methyl Ether	JAK2
Acorus tatarinowii Schott	Eugenol Methyl Ether	NOS1
Acorus tatarinowii Schott	Eugenol Methyl Ether	NOS3
Acorus tatarinowii Schott	Eugenol Methyl Ether	NQO1
Acorus tatarinowii Schott	Eugenol Methyl Ether	P2RX7
Acorus tatarinowii Schott	Eugenol Methyl Ether	PARP1
Acorus tatarinowii Schott	Eugenol Methyl Ether	PDE10A
Acorus tatarinowii Schott	Eugenol Methyl Ether	PIK3CA
Acorus tatarinowii Schott	Eugenol Methyl Ether	PIK3CB
Acorus tatarinowii Schott	Eugenol Methyl Ether	PTGS1
Acorus tatarinowii Schott	Eugenol Methyl Ether	RGS4
Acorus tatarinowii Schott	Eugenol Methyl Ether	TGM2
Acorus tatarinowii Schott	Isocembrol	AR
Acorus tatarinowii Schott	Isocembrol	BACE1
Acorus tatarinowii Schott	Isocembrol	CHRM1
Acorus tatarinowii Schott	Isocembrol	CNR2
Acorus tatarinowii Schott	Isocembrol	PSEN2
Acorus tatarinowii Schott	Isocembrol	PTPN1
Acorus tatarinowii Schott	Isocembrol	SPHK1
Acorus tatarinowii Schott	Isocembrol	SPHK2
Acorus tatarinowii Schott	kaempferol	ACHE
Acorus tatarinowii Schott	kaempferol	AHR
Acorus tatarinowii Schott	kaempferol	AKT1
Acorus tatarinowii Schott	kaempferol	ALOX5

Acorus tatarinowii Schott	kaempferol	AR
Acorus tatarinowii Schott	kaempferol	BAX
Acorus tatarinowii Schott	kaempferol	BCL2
Acorus tatarinowii Schott	kaempferol	CALB2
Acorus tatarinowii Schott	kaempferol	CASP3
Acorus tatarinowii Schott	kaempferol	CDK1
Acorus tatarinowii Schott	kaempferol	CHRM1
Acorus tatarinowii Schott	kaempferol	CHRM2
Acorus tatarinowii Schott	kaempferol	CYP1A2
Acorus tatarinowii Schott	kaempferol	CYP3A4
Acorus tatarinowii Schott	kaempferol	DPP4
Acorus tatarinowii Schott	kaempferol	F2
Acorus tatarinowii Schott	kaempferol	F7
Acorus tatarinowii Schott	kaempferol	HMOX1
Acorus tatarinowii Schott	kaempferol	ICAM1
Acorus tatarinowii Schott	kaempferol	INSR
Acorus tatarinowii Schott	kaempferol	JUN
Acorus tatarinowii Schott	kaempferol	MAPK8
Acorus tatarinowii Schott	kaempferol	MMP1
Acorus tatarinowii Schott	kaempferol	NOS2
Acorus tatarinowii Schott	kaempferol	NOS3
Acorus tatarinowii Schott	kaempferol	PGR
Acorus tatarinowii Schott	kaempferol	PIK3CG
Acorus tatarinowii Schott	kaempferol	PPARG
Acorus tatarinowii Schott	kaempferol	PRKACA
Acorus tatarinowii Schott	kaempferol	PTGS1
Acorus tatarinowii Schott	kaempferol	PTGS2
Acorus tatarinowii Schott	kaempferol	RELA
Acorus tatarinowii Schott	kaempferol	SELE
Acorus tatarinowii Schott	kaempferol	SLC6A2
Acorus tatarinowii Schott	kaempferol	SLPI
Acorus tatarinowii Schott	kaempferol	STAT1
Acorus tatarinowii Schott	kaempferol	TNF
Acorus tatarinowii Schott	kaempferol	VCAM1
Acorus tatarinowii Schott	kaempferol	XDH
Acorus tatarinowii Schott	Marmesin	ADRB2
Acorus tatarinowii Schott	Marmesin	AR
Acorus tatarinowii Schott	Marmesin	CDK2
Acorus tatarinowii Schott	Marmesin	CHRM1
Acorus tatarinowii Schott	Marmesin	CHRM2
Acorus tatarinowii Schott	Marmesin	DPP4
Acorus tatarinowii Schott	Marmesin	ESR2
Acorus tatarinowii Schott	Marmesin	F2
Acorus tatarinowii Schott	Marmesin	PIK3CG

Acorus tatarinowii Schott	Marmesin	PRKACA
Acorus tatarinowii Schott	Marmesin	PTGS1
Acorus tatarinowii Schott	Marmesin	PTGS2
Acorus tatarinowii Schott	Marmesin	RXRA
Acorus tatarinowii Schott	Marmesin	SLC6A4
Acorus tatarinowii Schott	Myristic Acid	ADRA2B
Acorus tatarinowii Schott	Myristic Acid	AGTR1
Acorus tatarinowii Schott	Myristic Acid	ALOX12
Acorus tatarinowii Schott	Myristic Acid	AR
Acorus tatarinowii Schott	Myristic Acid	CA1
Acorus tatarinowii Schott	Myristic Acid	CHRNA7
Acorus tatarinowii Schott	Myristic Acid	EDNRA
Acorus tatarinowii Schott	Myristic Acid	FABP3
Acorus tatarinowii Schott	Myristic Acid	G6PD
Acorus tatarinowii Schott	Myristic Acid	HSD11B1
Acorus tatarinowii Schott	Myristic Acid	MAPK1
Acorus tatarinowii Schott	Myristic Acid	MAPK14
Acorus tatarinowii Schott	Myristic Acid	MDM2
Acorus tatarinowii Schott	Myristic Acid	NR1H4
Acorus tatarinowii Schott	Myristic Acid	PLA2G4A
Acorus tatarinowii Schott	Myristic Acid	PLG
Acorus tatarinowii Schott	Myristic Acid	PPARA
Acorus tatarinowii Schott	Myristic Acid	PPARD
Acorus tatarinowii Schott	Myristic Acid	PPARG
Acorus tatarinowii Schott	Myristic Acid	PTPN1
Acorus tatarinowii Schott	Myristic Acid	RARA
Acorus tatarinowii Schott	Myristic Acid	RBP4
Acorus tatarinowii Schott	Myristic Acid	RXRA
Acorus tatarinowii Schott	Myristic Acid	TBXA2R
Acorus tatarinowii Schott	Myristic Acid	VDR
Acorus tatarinowii Schott	Nonanoic Acid	AKR1B1
Acorus tatarinowii Schott	Nonanoic Acid	AR
Acorus tatarinowii Schott	Nonanoic Acid	CA1
Acorus tatarinowii Schott	Nonanoic Acid	CHRNA7
Acorus tatarinowii Schott	Nonanoic Acid	CXCL8
Acorus tatarinowii Schott	Nonanoic Acid	FABP3
Acorus tatarinowii Schott	Nonanoic Acid	G6PD
Acorus tatarinowii Schott	Nonanoic Acid	HSD11B1
Acorus tatarinowii Schott	Nonanoic Acid	NR1H4
Acorus tatarinowii Schott	Nonanoic Acid	PLG
Acorus tatarinowii Schott	Nonanoic Acid	PPARA
Acorus tatarinowii Schott	Nonanoic Acid	PPARD
Acorus tatarinowii Schott	Nonanoic Acid	PTGS2
Acorus tatarinowii Schott	Nonanoic Acid	PTPRC

Acorus tatarinowii Schott	Nonanoic Acid	VDR
Acorus tatarinowii Schott	Octanoic Acid	AKR1B1
Acorus tatarinowii Schott	Octanoic Acid	AR
Acorus tatarinowii Schott	Octanoic Acid	CA1
Acorus tatarinowii Schott	Octanoic Acid	FABP3
Acorus tatarinowii Schott	Octanoic Acid	G6PD
Acorus tatarinowii Schott	Octanoic Acid	HSD11B1
Acorus tatarinowii Schott	Octanoic Acid	NR1H4
Acorus tatarinowii Schott	Octanoic Acid	PLG
Acorus tatarinowii Schott	Octanoic Acid	PPARA
Acorus tatarinowii Schott	Octanoic Acid	PPARD
Acorus tatarinowii Schott	Octanoic Acid	PTGS2
Acorus tatarinowii Schott	Octanoic Acid	PTPN1
Acorus tatarinowii Schott	Octanoic Acid	VDR
Acorus tatarinowii Schott	P-Methoxycinnamic Acid	AKR1B1
Acorus tatarinowii Schott	P-Methoxycinnamic Acid	ALOX5
Acorus tatarinowii Schott	P-Methoxycinnamic Acid	APEX1
Acorus tatarinowii Schott	P-Methoxycinnamic Acid	APP
Acorus tatarinowii Schott	P-Methoxycinnamic Acid	BACE1
Acorus tatarinowii Schott	P-Methoxycinnamic Acid	CA1
Acorus tatarinowii Schott	P-Methoxycinnamic Acid	CA4
Acorus tatarinowii Schott	P-Methoxycinnamic Acid	ESR2
Acorus tatarinowii Schott	P-Methoxycinnamic Acid	F3
Acorus tatarinowii Schott	P-Methoxycinnamic Acid	FOLH1
Acorus tatarinowii Schott	P-Methoxycinnamic Acid	GRK2
Acorus tatarinowii Schott	P-Methoxycinnamic Acid	GSK3B
Acorus tatarinowii Schott	P-Methoxycinnamic Acid	MAOB
Acorus tatarinowii Schott	P-Methoxycinnamic Acid	MAPK10
Acorus tatarinowii Schott	P-Methoxycinnamic Acid	MMP1
Acorus tatarinowii Schott	P-Methoxycinnamic Acid	MMP2
Acorus tatarinowii Schott	P-Methoxycinnamic Acid	MMP9
Acorus tatarinowii Schott	P-Methoxycinnamic Acid	PARP1
Acorus tatarinowii Schott	P-Methoxycinnamic Acid	PTGS2
Acorus tatarinowii Schott	P-Methoxycinnamic Acid	PTPN1
Acorus tatarinowii Schott	P-Methoxycinnamic Acid	PTPRC
Acorus tatarinowii Schott	P-Methoxycinnamic Acid	RELA
Acorus tatarinowii Schott	P-Methoxycinnamic Acid	TLR4
Acorus tatarinowii Schott	Thymol	ACHE
Acorus tatarinowii Schott	Thymol	ALB
Acorus tatarinowii Schott	Thymol	CDK2
Acorus tatarinowii Schott	Thymol	CDK2
Acorus tatarinowii Schott	Thymol	CHRM1
Acorus tatarinowii Schott	Thymol	CHRM2
Acorus tatarinowii Schott	Thymol	HTR2C

Acorus tatarinowii Schott	Thymol	JAK2
Acorus tatarinowii Schott	Thymol	PRKCA
Acorus tatarinowii Schott	Thymol	PTGS1
Acorus tatarinowii Schott	Thymol	SLC6A2
Acorus tatarinowii Schott	Thymol	SLC6A3
Acorus tatarinowii Schott	Thymol	SLC6A4
Acorus tatarinowii Schott	Thymol	TYR
Atractylodes macrocephala	(24S)-24-Propylcholesta-5-Ene-3beta-Ol	PGR
Atractylodes macrocephala	14-acetyl-12-senecioyl-2E,8Z,10E-atractylentriol	PTGS2
Atractylodes macrocephala	3β-acetoxyatractylone	ACHE
Atractylodes macrocephala	3β-acetoxyatractylone	ADRA1A
Atractylodes macrocephala	3β-acetoxyatractylone	ADRB2
Atractylodes macrocephala	3β-acetoxyatractylone	AR
Atractylodes macrocephala	3β-acetoxyatractylone	CHRM1
Atractylodes macrocephala	3β-acetoxyatractylone	CHRM2
Atractylodes macrocephala	3β-acetoxyatractylone	CHRNA7
Atractylodes macrocephala	3β-acetoxyatractylone	DPP4
Atractylodes macrocephala	3β-acetoxyatractylone	F2
Atractylodes macrocephala	3β-acetoxyatractylone	NOS3
Atractylodes macrocephala	3β-acetoxyatractylone	OPRM1
Atractylodes macrocephala	3β-acetoxyatractylone	PTGS2
Atractylodes macrocephala	3β-acetoxyatractylone	PTPN1
Atractylodes macrocephala	3β-acetoxyatractylone	RXRA
Atractylodes macrocephala	4-Ethoxycarbonyl-2-Quinolone	ACE
Atractylodes macrocephala	4-Ethoxycarbonyl-2-Quinolone	ADORA1
Atractylodes macrocephala	4-Ethoxycarbonyl-2-Quinolone	ADORA2A
Atractylodes macrocephala	4-Ethoxycarbonyl-2-Quinolone	APP
Atractylodes macrocephala	4-Ethoxycarbonyl-2-Quinolone	CASP3
Atractylodes macrocephala	4-Ethoxycarbonyl-2-Quinolone	CCR3
Atractylodes macrocephala	4-Ethoxycarbonyl-2-Quinolone	CDK2
Atractylodes macrocephala	4-Ethoxycarbonyl-2-Quinolone	CDK2
Atractylodes macrocephala	4-Ethoxycarbonyl-2-Quinolone	CDK5
Atractylodes macrocephala	4-Ethoxycarbonyl-2-Quinolone	CDK5
Atractylodes macrocephala	4-Ethoxycarbonyl-2-Quinolone	CHRM1
Atractylodes macrocephala	4-Ethoxycarbonyl-2-Quinolone	CHRM2
Atractylodes macrocephala	4-Ethoxycarbonyl-2-Quinolone	CRHR1
Atractylodes macrocephala	4-Ethoxycarbonyl-2-Quinolone	EGFR
Atractylodes macrocephala	4-Ethoxycarbonyl-2-Quinolone	ELANE
Atractylodes macrocephala	4-Ethoxycarbonyl-2-Quinolone	F2
Atractylodes macrocephala	4-Ethoxycarbonyl-2-Quinolone	FAAH
Atractylodes macrocephala	4-Ethoxycarbonyl-2-Quinolone	GALR3
Atractylodes macrocephala	4-Ethoxycarbonyl-2-Quinolone	GRM2

Atractylodes macrocephala	4-Ethoxycarbonyl-2-Quinolone	GSR
Atractylodes macrocephala	4-Ethoxycarbonyl-2-Quinolone	HMOX1
Atractylodes macrocephala	4-Ethoxycarbonyl-2-Quinolone	HSD11B1
Atractylodes macrocephala	4-Ethoxycarbonyl-2-Quinolone	HTR6
Atractylodes macrocephala	4-Ethoxycarbonyl-2-Quinolone	IDO1
Atractylodes macrocephala	4-Ethoxycarbonyl-2-Quinolone	LRRK2
Atractylodes macrocephala	4-Ethoxycarbonyl-2-Quinolone	MAOA
Atractylodes macrocephala	4-Ethoxycarbonyl-2-Quinolone	MAPK10
Atractylodes macrocephala	4-Ethoxycarbonyl-2-Quinolone	MAPK8
Atractylodes macrocephala	4-Ethoxycarbonyl-2-Quinolone	MPO
Atractylodes macrocephala	4-Ethoxycarbonyl-2-Quinolone	NOS1
Atractylodes macrocephala	4-Ethoxycarbonyl-2-Quinolone	NOS2
Atractylodes macrocephala	4-Ethoxycarbonyl-2-Quinolone	NR1H2
Atractylodes macrocephala	4-Ethoxycarbonyl-2-Quinolone	P2RX7
Atractylodes macrocephala	4-Ethoxycarbonyl-2-Quinolone	PARP1
Atractylodes macrocephala	4-Ethoxycarbonyl-2-Quinolone	PDE2A
Atractylodes macrocephala	4-Ethoxycarbonyl-2-Quinolone	PDE5A
Atractylodes macrocephala	4-Ethoxycarbonyl-2-Quinolone	PLAU
Atractylodes macrocephala	4-Ethoxycarbonyl-2-Quinolone	QPCT
Atractylodes macrocephala	4-Ethoxycarbonyl-2-Quinolone	SHH
Atractylodes macrocephala	4-Ethoxycarbonyl-2-Quinolone	SLC6A2
Atractylodes macrocephala	4-Ethoxycarbonyl-2-Quinolone	TERT
Atractylodes macrocephala	4-Ethoxycarbonyl-2-Quinolone	TGFBR1
Atractylodes macrocephala	4-Ethoxycarbonyl-2-Quinolone	TGFBR2
Atractylodes macrocephala	4-Ethoxycarbonyl-2-Quinolone	TGM2
Atractylodes macrocephala	4-Ethoxycarbonyl-2-Quinolone	XDH
Atractylodes macrocephala	8β-ethoxy atractylenolide III	CHRNA7
Atractylodes macrocephala	8β-ethoxy atractylenolide III	PTGS2
Atractylodes macrocephala	8β-ethoxy atractylenolide III	PTPN1
Atractylodes macrocephala	Atractylenolide I	AR
Atractylodes macrocephala	Atractylenolide I	CHRM1
Atractylodes macrocephala	Atractylenolide I	CHRM2
Atractylodes macrocephala	Atractylenolide I	CYP11B2
Atractylodes macrocephala	Atractylenolide I	KIF11
Atractylodes macrocephala	Atractylenolide I	MPO
Atractylodes macrocephala	Atractylenolide I	PGR
Atractylodes macrocephala	Atractylenolide I	PTPRC
Atractylodes macrocephala	Atractylenolide I	TYMS
Atractylodes macrocephala	Atractylenolide Iii	AR
Atractylodes macrocephala	Atractylenolide Iii	HMOX1
Atractylodes macrocephala	Atractylenolide Iii	MDM2
Atractylodes macrocephala	Atractylenolide Iii	P2RX7
Atractylodes macrocephala	Atractylenolide Iii	PARP1
Atractylodes macrocephala	Atractylenolide Iii	PGR

Atractylodes macrocephala	Atractylenolide Iii	PLA2G1B
Atractylodes macrocephala	Atractylenolide Iii	PLA2G2A
Atractylodes macrocephala	Atractylenolide Iii	PTK2B
Atractylodes macrocephala	Atractylenolide Iii	PTPN1
Atractylodes macrocephala	Atractylenolide Iii	SIGMAR1
Atractylodes macrocephala	Atractylenolide Iii	SLC6A2
Atractylodes macrocephala	Atractylenolide Iii	SLC6A3
Atractylodes macrocephala	Atractylenolide Iii	SLC6A4
Atractylodes macrocephala	Beta-Eudesmol	ACHE
Atractylodes macrocephala	Beta-Eudesmol	AR
Atractylodes macrocephala	Beta-Eudesmol	BCHE
Atractylodes macrocephala	Beta-Eudesmol	CHRM2
Atractylodes macrocephala	Beta-Eudesmol	CNR2
Atractylodes macrocephala	Beta-Eudesmol	CYP2C19
Atractylodes macrocephala	Beta-Eudesmol	ESR1
Atractylodes macrocephala	Beta-Eudesmol	HMGCR
Atractylodes macrocephala	Beta-Eudesmol	HSD11B1
Atractylodes macrocephala	Beta-Eudesmol	PSEN2
Atractylodes macrocephala	Beta-Eudesmol	PTGS1
Atractylodes macrocephala	Beta-Eudesmol	SLC6A2
Atractylodes macrocephala	Beta-Eudesmol	SLC6A4
Atractylodes macrocephala	Hinesol	ACHE
Atractylodes macrocephala	Hinesol	AR
Atractylodes macrocephala	Hinesol	BCHE
Atractylodes macrocephala	Hinesol	CHRM2
Atractylodes macrocephala	Hinesol	CYP2C19
Atractylodes macrocephala	Hinesol	ESR1
Atractylodes macrocephala	Hinesol	ESR2
Atractylodes macrocephala	Hinesol	FABP3
Atractylodes macrocephala	Hinesol	G6PD
Atractylodes macrocephala	Hinesol	HMGCR
Atractylodes macrocephala	Hinesol	HSD11B1
Atractylodes macrocephala	Hinesol	NR3C1
Atractylodes macrocephala	Hinesol	PPARA
Atractylodes macrocephala	Hinesol	PPARD
Atractylodes macrocephala	Hinesol	PREP
Atractylodes macrocephala	Hinesol	PTPN1
Atractylodes macrocephala	Hinesol	PTPN11
Atractylodes macrocephala	Hinesol	PTPN6
Atractylodes macrocephala	Hinesol	SLC6A2
Atractylodes macrocephala	Hinesol	SLC6A4
Atractylodes macrocephala	Hinesol	SREBF2
Atractylodes macrocephala	Hinesol	TERT
Atractylodes macrocephala	Hinesol	TOP1

Gastrodia elata	p-hydroxybenzyl ethyl ether	ADORA1
Gastrodia elata	p-hydroxybenzyl ethyl ether	ADORA2A
Gastrodia elata	p-hydroxybenzyl ethyl ether	ALOX5
Gastrodia elata	p-hydroxybenzyl ethyl ether	CA1
Gastrodia elata	p-hydroxybenzyl ethyl ether	DRD3
Gastrodia elata	p-hydroxybenzyl ethyl ether	ESR2
Gastrodia elata	p-hydroxybenzyl ethyl ether	PTGS2
Gastrodia elata	p-hydroxybenzyl ethyl ether	SLC6A2
Gastrodia elata	3,4-Dihydroxybenzaldehyde	CA1
Gastrodia elata	3,4-Dihydroxybenzaldehyde	CA4
Gastrodia elata	3,4-Dihydroxybenzaldehyde	COMT
Gastrodia elata	3,4-Dihydroxybenzaldehyde	ERN1
Gastrodia elata	3,4-Dihydroxybenzaldehyde	TYR
Gastrodia elata	4-(4'-Hydroxybenzyloxy)Benzyl Methyl Ether	ABCC9
Gastrodia elata	4-(4'-Hydroxybenzyloxy)Benzyl Methyl Ether	ACHE
Gastrodia elata	4-(4'-Hydroxybenzyloxy)Benzyl Methyl Ether	ADAM17
Gastrodia elata	4-(4'-Hydroxybenzyloxy)Benzyl Methyl Ether	AKT1
Gastrodia elata	4-(4'-Hydroxybenzyloxy)Benzyl Methyl Ether	ALOX5
Gastrodia elata	4-(4'-Hydroxybenzyloxy)Benzyl Methyl Ether	ALPL
Gastrodia elata	4-(4'-Hydroxybenzyloxy)Benzyl Methyl Ether	CA1
Gastrodia elata	4-(4'-Hydroxybenzyloxy)Benzyl Methyl Ether	CACNA1C
Gastrodia elata	4-(4'-Hydroxybenzyloxy)Benzyl Methyl Ether	CDK1
Gastrodia elata	4-(4'-Hydroxybenzyloxy)Benzyl Methyl Ether	CDK2
Gastrodia elata	4-(4'-Hydroxybenzyloxy)Benzyl Methyl Ether	CDK2
Gastrodia elata	4-(4'-Hydroxybenzyloxy)Benzyl Methyl Ether	COMT
Gastrodia elata	4-(4'-Hydroxybenzyloxy)Benzyl Methyl Ether	CREBBP
Gastrodia elata	4-(4'-Hydroxybenzyloxy)Benzyl Methyl Ether	DYRK1A
Gastrodia elata	4-(4'-Hydroxybenzyloxy)Benzyl Methyl Ether	EIF2AK3

Gastrodia elata	4-(4'-Hydroxybenzyloxy)Benzyl Methyl Ether	ESR2
Gastrodia elata	4-(4'-Hydroxybenzyloxy)Benzyl Methyl Ether	F2
Gastrodia elata	4-(4'-Hydroxybenzyloxy)Benzyl Methyl Ether	FLT1
Gastrodia elata	4-(4'-Hydroxybenzyloxy)Benzyl Methyl Ether	HDAC1
Gastrodia elata	4-(4'-Hydroxybenzyloxy)Benzyl Methyl Ether	HMGCR
Gastrodia elata	4-(4'-Hydroxybenzyloxy)Benzyl Methyl Ether	HSD11B1
Gastrodia elata	4-(4'-Hydroxybenzyloxy)Benzyl Methyl Ether	JAK2
Gastrodia elata	4-(4'-Hydroxybenzyloxy)Benzyl Methyl Ether	KDR
Gastrodia elata	4-(4'-Hydroxybenzyloxy)Benzyl Methyl Ether	KIF11
Gastrodia elata	4-(4'-Hydroxybenzyloxy)Benzyl Methyl Ether	KIT
Gastrodia elata	4-(4'-Hydroxybenzyloxy)Benzyl Methyl Ether	LRRK2
Gastrodia elata	4-(4'-Hydroxybenzyloxy)Benzyl Methyl Ether	MAOB
Gastrodia elata	4-(4'-Hydroxybenzyloxy)Benzyl Methyl Ether	MAP2K1
Gastrodia elata	4-(4'-Hydroxybenzyloxy)Benzyl Methyl Ether	MAPK14
Gastrodia elata	4-(4'-Hydroxybenzyloxy)Benzyl Methyl Ether	MME
Gastrodia elata	4-(4'-Hydroxybenzyloxy)Benzyl Methyl Ether	MMP1
Gastrodia elata	4-(4'-Hydroxybenzyloxy)Benzyl Methyl Ether	MMP2
Gastrodia elata	4-(4'-Hydroxybenzyloxy)Benzyl Methyl Ether	MMP3
Gastrodia elata	4-(4'-Hydroxybenzyloxy)Benzyl Methyl Ether	MMP9
Gastrodia elata	4-(4'-Hydroxybenzyloxy)Benzyl Methyl Ether	MTOR
Gastrodia elata	4-(4'-Hydroxybenzyloxy)Benzyl Methyl Ether	NLRP3
Gastrodia elata	4-(4'-Hydroxybenzyloxy)Benzyl Methyl Ether	NR3C1

Gastrodia elata	4-(4'-Hydroxybenzyloxy)Benzyl Methyl Ether	NTRK1
Gastrodia elata	4-(4'-Hydroxybenzyloxy)Benzyl Methyl Ether	OPRM1
Gastrodia elata	4-(4'-Hydroxybenzyloxy)Benzyl Methyl Ether	PCNA
Gastrodia elata	4-(4'-Hydroxybenzyloxy)Benzyl Methyl Ether	PDE10A
Gastrodia elata	4-(4'-Hydroxybenzyloxy)Benzyl Methyl Ether	PDE2A
Gastrodia elata	4-(4'-Hydroxybenzyloxy)Benzyl Methyl Ether	PIK3CA
Gastrodia elata	4-(4'-Hydroxybenzyloxy)Benzyl Methyl Ether	PIK3CB
Gastrodia elata	4-(4'-Hydroxybenzyloxy)Benzyl Methyl Ether	PIK3CD
Gastrodia elata	4-(4'-Hydroxybenzyloxy)Benzyl Methyl Ether	PIK3CG
Gastrodia elata	4-(4'-Hydroxybenzyloxy)Benzyl Methyl Ether	PLA2G7
Gastrodia elata	4-(4'-Hydroxybenzyloxy)Benzyl Methyl Ether	PRKCB
Gastrodia elata	4-(4'-Hydroxybenzyloxy)Benzyl Methyl Ether	PTGS1
Gastrodia elata	4-(4'-Hydroxybenzyloxy)Benzyl Methyl Ether	PTGS2
Gastrodia elata	4-(4'-Hydroxybenzyloxy)Benzyl Methyl Ether	RET
Gastrodia elata	4-(4'-Hydroxybenzyloxy)Benzyl Methyl Ether	ROCK2
Gastrodia elata	4-(4'-Hydroxybenzyloxy)Benzyl Methyl Ether	SCN9A
Gastrodia elata	4-(4'-Hydroxybenzyloxy)Benzyl Methyl Ether	SLC6A2
Gastrodia elata	4-(4'-Hydroxybenzyloxy)Benzyl Methyl Ether	XIAP
Gastrodia elata	4,4'-Dihydroxydiphenylmethane	ALOX12
Gastrodia elata	4,4'-Dihydroxydiphenylmethane	ALOX5
Gastrodia elata	4,4'-Dihydroxydiphenylmethane	ALPL
Gastrodia elata	4,4'-Dihydroxydiphenylmethane	AR
Gastrodia elata	4,4'-Dihydroxydiphenylmethane	BCL2
Gastrodia elata	4,4'-Dihydroxydiphenylmethane	BCL2L1
Gastrodia elata	4,4'-Dihydroxydiphenylmethane	CA4
Gastrodia elata	4,4'-Dihydroxydiphenylmethane	CDK5R1

Gastrodia elata	4,4'-Dihydroxydiphenylmethane	DAO
Gastrodia elata	4,4'-Dihydroxydiphenylmethane	DYRK1A
Gastrodia elata	4,4'-Dihydroxydiphenylmethane	ESR1
Gastrodia elata	4,4'-Dihydroxydiphenylmethane	ESR2
Gastrodia elata	4,4'-Dihydroxydiphenylmethane	HTR6
Gastrodia elata	4,4'-Dihydroxydiphenylmethane	IDO1
Gastrodia elata	4,4'-Dihydroxydiphenylmethane	MMP1
Gastrodia elata	4,4'-Dihydroxydiphenylmethane	MMP2
Gastrodia elata	4,4'-Dihydroxydiphenylmethane	MMP9
Gastrodia elata	4,4'-Dihydroxydiphenylmethane	PARP1
Gastrodia elata	4,4'-Dihydroxydiphenylmethane	PTGS1
Gastrodia elata	4,4'-Dihydroxydiphenylmethane	PTGS2
Gastrodia elata	4,4'-Dihydroxydiphenylmethane	SLC6A2
Gastrodia elata	4-hydroxybenzaldehyde	ACHE
Gastrodia elata	4-hydroxybenzaldehyde	CA1
Gastrodia elata	4-hydroxybenzaldehyde	CA4
Gastrodia elata	4-hydroxybenzaldehyde	COMT
Gastrodia elata	4-hydroxybenzaldehyde	ERN1
Gastrodia elata	4-hydroxybenzaldehyde	TYR
Gastrodia elata	4-Hydroxybenzyl alcohol	ACHE
Gastrodia elata	4-Hydroxybenzyl alcohol	CA1
Gastrodia elata	4-Hydroxybenzyl alcohol	CA4
Gastrodia elata	4-Hydroxybenzyl alcohol	HTR3A
Gastrodia elata	4-hydroxybenzyl methyl ether	ACHE
Gastrodia elata	4-hydroxybenzyl methyl ether	CA1
Gastrodia elata	4-hydroxybenzyl methyl ether	PTGS1
Gastrodia elata	4-hydroxybenzyl methyl ether	PTGS2
Gastrodia elata	Bis(4-Hydroxybenzyl) Ether	ADORA1
Gastrodia elata	Bis(4-Hydroxybenzyl) Ether	ADORA2A
Gastrodia elata	Bis(4-Hydroxybenzyl) Ether	CA1
Gastrodia elata	Bis(4-Hydroxybenzyl) Ether	DYRK1A
Gastrodia elata	Bis(4-Hydroxybenzyl) Ether	ESR1
Gastrodia elata	Bis(4-Hydroxybenzyl) Ether	ESR2
Gastrodia elata	Citronellal	ACE
Gastrodia elata	Citronellal	ADH1C
Gastrodia elata	Citronellal	AR
Gastrodia elata	Citronellal	CCND1
Gastrodia elata	Citronellal	CHRM1
Gastrodia elata	Citronellal	CTSB
Gastrodia elata	Citronellal	CTSD
Gastrodia elata	Citronellal	CTSL
Gastrodia elata	Citronellal	DRD2
Gastrodia elata	Citronellal	FAAH
Gastrodia elata	Citronellal	FABP3

Gastrodia elata	Citronellal	HDAC1
Gastrodia elata	Citronellal	HDAC6
Gastrodia elata	Citronellal	JAK2
Gastrodia elata	Citronellal	MAOA
Gastrodia elata	Citronellal	MAOB
Gastrodia elata	Citronellal	MMP1
Gastrodia elata	Citronellal	PARP1
Gastrodia elata	Citronellal	PGR
Gastrodia elata	Citronellal	PPARA
Gastrodia elata	Citronellal	PPARD
Gastrodia elata	Citronellal	PPARG
Gastrodia elata	Citronellal	PSEN2
Gastrodia elata	Citronellal	PTGS1
Gastrodia elata	Citronellal	SIGMAR1
Gastrodia elata	Dauricine	ABCB1
Gastrodia elata	Dauricine	ABL1
Gastrodia elata	Dauricine	ACHE
Gastrodia elata	Dauricine	ADRA1A
Gastrodia elata	Dauricine	ADRA2A
Gastrodia elata	Dauricine	ADRB2
Gastrodia elata	Dauricine	ADRB3
Gastrodia elata	Dauricine	AKT2
Gastrodia elata	Dauricine	BCHE
Gastrodia elata	Dauricine	BRAF
Gastrodia elata	Dauricine	CDK4
Gastrodia elata	Dauricine	CSF1R
Gastrodia elata	Dauricine	DPP4
Gastrodia elata	Dauricine	DRD1
Gastrodia elata	Dauricine	DRD2
Gastrodia elata	Dauricine	DRD3
Gastrodia elata	Dauricine	ESR1
Gastrodia elata	Dauricine	ESR2
Gastrodia elata	Dauricine	F3
Gastrodia elata	Dauricine	FGFR1
Gastrodia elata	Dauricine	FGFR3
Gastrodia elata	Dauricine	HRH3
Gastrodia elata	Dauricine	HTR1A
Gastrodia elata	Dauricine	HTR2A
Gastrodia elata	Dauricine	HTR3A
Gastrodia elata	Dauricine	IGF1R
Gastrodia elata	Dauricine	KCNN2
Gastrodia elata	Dauricine	KCNN3
Gastrodia elata	Dauricine	KIT
Gastrodia elata	Dauricine	MAOA

Gastrodia elata	Dauricine	MAP2
Gastrodia elata	Dauricine	MAPK10
Gastrodia elata	Dauricine	MAPK7
Gastrodia elata	Dauricine	MAPK8
Gastrodia elata	Dauricine	MTOR
Gastrodia elata	Dauricine	OPRM1
Gastrodia elata	Dauricine	PDE1A
Gastrodia elata	Dauricine	PIK3CA
Gastrodia elata	Dauricine	PRKCD
Gastrodia elata	Dauricine	PSEN2
Gastrodia elata	Dauricine	RAF1
Gastrodia elata	Dauricine	RET
Gastrodia elata	Dauricine	SELP
Gastrodia elata	Dauricine	SIGMAR1
Gastrodia elata	Dauricine	SLC18A2
Gastrodia elata	Dauricine	SLC6A2
Gastrodia elata	Dauricine	SLC6A3
Gastrodia elata	Dauricine	SLC6A4
Gastrodia elata	Dauricine	SYK
Gastrodia elata	Dauricine	TBXA2R
Gastrodia elata	Dauricine	TEK
Gastrodia elata	Dauricine	TERT
Gastrodia elata	Dauricine	TGFBR1
Gastrodia elata	Gamma-Sitosterol	ACHE
Gastrodia elata	Gamma-Sitosterol	AR
Gastrodia elata	Gamma-Sitosterol	BCHE
Gastrodia elata	Gamma-Sitosterol	CHRM2
Gastrodia elata	Gamma-Sitosterol	CYP2C19
Gastrodia elata	Gamma-Sitosterol	ESR1
Gastrodia elata	Gamma-Sitosterol	ESR2
Gastrodia elata	Gamma-Sitosterol	G6PD
Gastrodia elata	Gamma-Sitosterol	HMGCR
Gastrodia elata	Gamma-Sitosterol	HSD11B1
Gastrodia elata	Gamma-Sitosterol	NOS2
Gastrodia elata	Gamma-Sitosterol	NR1H2
Gastrodia elata	Gamma-Sitosterol	PPARD
Gastrodia elata	Gamma-Sitosterol	PPARG
Gastrodia elata	Gamma-Sitosterol	PTPN1
Gastrodia elata	Gamma-Sitosterol	PTPN6
Gastrodia elata	Gamma-Sitosterol	SLC6A2
Gastrodia elata	Gamma-Sitosterol	SLC6A4
Gastrodia elata	Gamma-Sitosterol	SREBF2
Gastrodia elata	Gamma-Sitosterol	VDR
Gastrodia elata	Gastrodin	ADA

Gastrodia elata	Gastrodin	ADORA1
Gastrodia elata	Gastrodin	ADORA2A
Gastrodia elata	Gastrodin	AKR1B1
Gastrodia elata	Gastrodin	FOLH1
Gastrodia elata	Gastrodin	FUCA1
Gastrodia elata	Gastrodin	TYR
Gastrodia elata	Gastrodioside	AKR1B1
Gastrodia elata	Gastrodioside	HSP90AA1
Gastrodia elata	Gastrodioside	TYR
Gastrodia elata	M-Hydroxybenzoic Acid	ALB
Gastrodia elata	M-Hydroxybenzoic Acid	BCL2L1
Gastrodia elata	M-Hydroxybenzoic Acid	CA1
Gastrodia elata	M-Hydroxybenzoic Acid	CA4
Gastrodia elata	M-Hydroxybenzoic Acid	DAO
Gastrodia elata	M-Hydroxybenzoic Acid	HDAC6
Gastrodia elata	M-Hydroxybenzoic Acid	TTR
Gastrodia elata	P-Hydroxybenzaldehyde	ACHE
Gastrodia elata	P-Hydroxybenzaldehyde	CA1
Gastrodia elata	P-Hydroxybenzaldehyde	CA4
Gastrodia elata	P-Hydroxybenzaldehyde	COMT
Gastrodia elata	P-Hydroxybenzaldehyde	ERN1
Gastrodia elata	P-Hydroxybenzaldehyde	TYR
Gastrodia elata	P-Hydroxybenzyl Alcohol	ACHE
Gastrodia elata	P-Hydroxybenzyl Alcohol	CA1
Gastrodia elata	P-Hydroxybenzyl Alcohol	CA4
Gastrodia elata	P-Hydroxybenzyl Alcohol	HTR3A
Morindae Officinalis Radix	(2R,3S)-(+)-3',5-Dihydroxy-4 ,7-dimethoxydihydroflavonol	CALB2
Morindae Officinalis Radix	(2R,3S)-(+)-3',5-Dihydroxy-4 ,7-dimethoxydihydroflavonol	F10
Morindae Officinalis Radix	(2R,3S)-(+)-3',5-Dihydroxy-4 ,7-dimethoxydihydroflavonol	GRM2
Morindae Officinalis Radix	(2R,3S)-(+)-3',5-Dihydroxy-4 ,7-dimethoxydihydroflavonol	PIK3CG
Morindae Officinalis Radix	(2R,3S)-(+)-3',5-Dihydroxy-4 ,7-dimethoxydihydroflavonol	PTGS1
Morindae Officinalis Radix	(2R,3S)-(+)-3',5-Dihydroxy-4 ,7-dimethoxydihydroflavonol	PTGS2
Morindae Officinalis Radix	1,5,7-trihydroxy-6-methoxy-2-methoxymethylanthracenequinone	CALB2
Morindae Officinalis Radix	1,5,7-trihydroxy-6-methoxy-2-methoxymethylanthracenequinone	F10
Morindae Officinalis Radix	1,5,7-trihydroxy-6-methoxy-2-methoxymethylanthracenequinone	F7

Morindae Officinalis Radix	1,5,7-trihydroxy-6-methoxy-2-methoxymethylanthracenequinone	PTGS1
Morindae Officinalis Radix	1,5,7-trihydroxy-6-methoxy-2-methoxymethylanthracenequinone	PTGS2
Morindae Officinalis Radix	1,6-Dihydroxy-2-Methoxyanthraquinone	ACHE
Morindae Officinalis Radix	1,6-Dihydroxy-2-Methoxyanthraquinone	ADORA1
Morindae Officinalis Radix	1,6-Dihydroxy-2-Methoxyanthraquinone	ADORA2A
Morindae Officinalis Radix	1,6-Dihydroxy-2-Methoxyanthraquinone	ALK
Morindae Officinalis Radix	1,6-Dihydroxy-2-Methoxyanthraquinone	AXL
Morindae Officinalis Radix	1,6-Dihydroxy-2-Methoxyanthraquinone	BCL2
Morindae Officinalis Radix	1,6-Dihydroxy-2-Methoxyanthraquinone	CXCR2
Morindae Officinalis Radix	1,6-Dihydroxy-2-Methoxyanthraquinone	EGFR
Morindae Officinalis Radix	1,6-Dihydroxy-2-Methoxyanthraquinone	ELANE
Morindae Officinalis Radix	1,6-Dihydroxy-2-Methoxyanthraquinone	ESR1
Morindae Officinalis Radix	1,6-Dihydroxy-2-Methoxyanthraquinone	ESR2
Morindae Officinalis Radix	1,6-Dihydroxy-2-Methoxyanthraquinone	HTR2A
Morindae Officinalis Radix	1,6-Dihydroxy-2-Methoxyanthraquinone	KDR
Morindae Officinalis Radix	1,6-Dihydroxy-2-Methoxyanthraquinone	MIF
Morindae Officinalis Radix	1,6-Dihydroxy-2-Methoxyanthraquinone	PARP1
Morindae Officinalis Radix	1,6-Dihydroxy-2-Methoxyanthraquinone	XDH
Morindae Officinalis Radix	1,6-dihydroxy-5-methoxy-2-(methoxymethyl)-9,10-anthraquinone	CALB2
Morindae Officinalis Radix	1,6-dihydroxy-5-methoxy-2-(methoxymethyl)-9,10-anthraquinone	F10

Morindae Officinalis Radix	1,6-dihydroxy-5-methoxy-2-(methoxymethyl)-9,10-anthraquinone	F7
Morindae Officinalis Radix	1,6-dihydroxy-5-methoxy-2-(methoxymethyl)-9,10-anthraquinone	PIK3CG
Morindae Officinalis Radix	1,6-dihydroxy-5-methoxy-2-(methoxymethyl)-9,10-anthraquinone	PTGS1
Morindae Officinalis Radix	1,6-dihydroxy-5-methoxy-2-(methoxymethyl)-9,10-anthraquinone	PTGS2
Morindae Officinalis Radix	1,6-dihydroxy-5-methoxy-2-(methoxymethyl)-9,10-anthraquinone	RXRA
Morindae Officinalis Radix	1-Hydroxy-2-Methyl-Anthraquinone	ADORA1
Morindae Officinalis Radix	1-Hydroxy-2-Methyl-Anthraquinone	BACE1
Morindae Officinalis Radix	1-Hydroxy-2-Methyl-Anthraquinone	BCL2
Morindae Officinalis Radix	1-Hydroxy-2-Methyl-Anthraquinone	CSNK1D
Morindae Officinalis Radix	1-Hydroxy-2-Methyl-Anthraquinone	EGFR
Morindae Officinalis Radix	1-Hydroxy-2-Methyl-Anthraquinone	ELANE
Morindae Officinalis Radix	1-Hydroxy-2-Methyl-Anthraquinone	ESR1
Morindae Officinalis Radix	1-Hydroxy-2-Methyl-Anthraquinone	ESR2
Morindae Officinalis Radix	1-Hydroxy-2-Methyl-Anthraquinone	FGFR1
Morindae Officinalis Radix	1-Hydroxy-2-Methyl-Anthraquinone	HMGCR
Morindae Officinalis Radix	1-Hydroxy-2-Methyl-Anthraquinone	HSP90AA1
Morindae Officinalis Radix	1-Hydroxy-2-Methyl-Anthraquinone	HTR1A
Morindae Officinalis Radix	1-Hydroxy-2-Methyl-Anthraquinone	MAOB
Morindae Officinalis Radix	1-Hydroxy-2-Methyl-Anthraquinone	MSR1

Morindae Officinalis Radix	1-Hydroxy-2-Methyl-Anthraquinone	OPRM1
Morindae Officinalis Radix	1-Hydroxy-2-Methyl-Anthraquinone	PARP1
Morindae Officinalis Radix	1-hydroxy-6-hydroxymethylanthracenequinone	ADRB2
Morindae Officinalis Radix	1-hydroxy-6-hydroxymethylanthracenequinone	CHRNA7
Morindae Officinalis Radix	1-hydroxy-6-hydroxymethylanthracenequinone	PDE3A
Morindae Officinalis Radix	1-hydroxy-6-hydroxymethylanthracenequinone	PIK3CG
Morindae Officinalis Radix	1-hydroxy-6-hydroxymethylanthracenequinone	PRKACA
Morindae Officinalis Radix	1-hydroxy-6-hydroxymethylanthracenequinone	PTGS1
Morindae Officinalis Radix	1-hydroxy-6-hydroxymethylanthracenequinone	PTGS2
Morindae Officinalis Radix	2-hydroxy-1,5-dimethoxy-6-(methoxymethyl)-9,10-anthraquinone	CALB2
Morindae Officinalis Radix	2-hydroxy-1,5-dimethoxy-6-(methoxymethyl)-9,10-anthraquinone	F10
Morindae Officinalis Radix	2-hydroxy-1,5-dimethoxy-6-(methoxymethyl)-9,10-anthraquinone	F7
Morindae Officinalis Radix	2-hydroxy-1,5-dimethoxy-6-(methoxymethyl)-9,10-anthraquinone	KCNMA1
Morindae Officinalis Radix	2-hydroxy-1,5-dimethoxy-6-(methoxymethyl)-9,10-anthraquinone	PIK3CG
Morindae Officinalis Radix	2-hydroxy-1,5-dimethoxy-6-(methoxymethyl)-9,10-anthraquinone	PTGS1
Morindae Officinalis Radix	2-hydroxy-1,5-dimethoxy-6-(methoxymethyl)-9,10-anthraquinone	PTGS2
Morindae Officinalis Radix	2-hydroxy-1,5-dimethoxy-6-(methoxymethyl)-9,10-anthraquinone	RXRA
Morindae Officinalis Radix	2-hydroxy-1,8-dimethoxy-7-methoxymethylanthracenequinone	CALB2

Morindae Officinalis Radix	2-hydroxy-1,8-dimethoxy-7-methoxymethylanthracenequinone	F10
Morindae Officinalis Radix	2-hydroxy-1,8-dimethoxy-7-methoxymethylanthracenequinone	F7
Morindae Officinalis Radix	2-hydroxy-1,8-dimethoxy-7-methoxymethylanthracenequinone	KCNMA1
Morindae Officinalis Radix	2-hydroxy-1,8-dimethoxy-7-methoxymethylanthracenequinone	PIK3CG
Morindae Officinalis Radix	2-hydroxy-1,8-dimethoxy-7-methoxymethylanthracenequinone	PTGS1
Morindae Officinalis Radix	2-hydroxy-1,8-dimethoxy-7-methoxymethylanthracenequinone	PTGS2
Morindae Officinalis Radix	2-hydroxy-1,8-dimethoxy-7-methoxymethylanthracenequinone	RXRA
Morindae Officinalis Radix	2-Hydroxyanthraquinone	BCL2
Morindae Officinalis Radix	2-Hydroxyanthraquinone	BCL2L1
Morindae Officinalis Radix	2-Hydroxyanthraquinone	DYRK1A
Morindae Officinalis Radix	2-Hydroxyanthraquinone	ELANE
Morindae Officinalis Radix	2-Hydroxyanthraquinone	ESR1
Morindae Officinalis Radix	2-Hydroxyanthraquinone	ESR2
Morindae Officinalis Radix	2-Hydroxyanthraquinone	HTR2A
Morindae Officinalis Radix	2-Hydroxyanthraquinone	IDH1
Morindae Officinalis Radix	3beta,20(R),5-alkenyl-stigmastol	GRM2
Morindae Officinalis Radix	3beta,20(R),5-alkenyl-stigmastol	PGR
Morindae Officinalis Radix	3beta-24S(R)-butyl-5-alkenyl-cholestol	PGR
Morindae Officinalis Radix	Alizarin-2-methylether	CHRNA7
Morindae Officinalis Radix	Alizarin-2-methylether	ESR1
Morindae Officinalis Radix	Alizarin-2-methylether	PDE3A
Morindae Officinalis Radix	Alizarin-2-methylether	PIK3CG
Morindae Officinalis Radix	Alizarin-2-methylether	PRKACA
Morindae Officinalis Radix	Alizarin-2-methylether	PTGS1
Morindae Officinalis Radix	Alizarin-2-methylether	PTGS2
Morindae Officinalis Radix	Alizarin-2-methylether	RXRA
Morindae Officinalis Radix	Alizarin-2-methylether	SLC6A4
Morindae Officinalis Radix	americanin A	CCNA2
Morindae Officinalis Radix	americanin A	CDK2
Morindae Officinalis Radix	americanin A	DPP4
Morindae Officinalis Radix	americanin A	ESR1
Morindae Officinalis Radix	americanin A	GSK3B
Morindae Officinalis Radix	americanin A	PRKACA
Morindae Officinalis Radix	americanin A	PTGS2
Morindae Officinalis Radix	Ascorbic Acid	GSK3B
Morindae Officinalis Radix	beta-sitosterol	ADRA1A

Morindae Officinalis Radix	beta-sitosterol	ADRB2
Morindae Officinalis Radix	beta-sitosterol	BAX
Morindae Officinalis Radix	beta-sitosterol	BCL2
Morindae Officinalis Radix	beta-sitosterol	CASP3
Morindae Officinalis Radix	beta-sitosterol	CASP8
Morindae Officinalis Radix	beta-sitosterol	CASP9
Morindae Officinalis Radix	beta-sitosterol	CHRM1
Morindae Officinalis Radix	beta-sitosterol	CHRM2
Morindae Officinalis Radix	beta-sitosterol	CHRNA7
Morindae Officinalis Radix	beta-sitosterol	DRD1
Morindae Officinalis Radix	beta-sitosterol	HTR2A
Morindae Officinalis Radix	beta-sitosterol	JUN
Morindae Officinalis Radix	beta-sitosterol	MAP2
Morindae Officinalis Radix	beta-sitosterol	OPRM1
Morindae Officinalis Radix	beta-sitosterol	PDE3A
Morindae Officinalis Radix	beta-sitosterol	PGR
Morindae Officinalis Radix	beta-sitosterol	PIK3CG
Morindae Officinalis Radix	beta-sitosterol	PON1
Morindae Officinalis Radix	beta-sitosterol	PRKACA
Morindae Officinalis Radix	beta-sitosterol	PRKCA
Morindae Officinalis Radix	beta-sitosterol	PTGS1
Morindae Officinalis Radix	beta-sitosterol	PTGS2
Morindae Officinalis Radix	beta-sitosterol	SLC6A4
Morindae Officinalis Radix	beta-sitosterol	TGFB1
Morindae Officinalis Radix	Diop	ADRB2
Morindae Officinalis Radix	isoprincepin	ACHE
Morindae Officinalis Radix	Ohioensin-A	PTGS1
Morindae Officinalis Radix	Ohioensin-A	PTGS2
Morindae Officinalis Radix	Physcion	ADAM17
Morindae Officinalis Radix	Physcion	BCHE
Morindae Officinalis Radix	Physcion	BCL2
Morindae Officinalis Radix	Physcion	CHRNA7
Morindae Officinalis Radix	Physcion	EGFR
Morindae Officinalis Radix	Physcion	EIF2AK2
Morindae Officinalis Radix	Physcion	ELANE
Morindae Officinalis Radix	Physcion	ESR1
Morindae Officinalis Radix	Physcion	ESR2
Morindae Officinalis Radix	Physcion	FLT1
Morindae Officinalis Radix	Physcion	FLT4
Morindae Officinalis Radix	Physcion	HDAC1
Morindae Officinalis Radix	Physcion	HDAC4
Morindae Officinalis Radix	Physcion	HDAC6
Morindae Officinalis Radix	Physcion	HDAC9
Morindae Officinalis Radix	Physcion	KCNMA1

Morindae Officinalis Radix	Physcion	KDR
Morindae Officinalis Radix	Physcion	LRRK2
Morindae Officinalis Radix	Physcion	MAP2K1
Morindae Officinalis Radix	Physcion	MAPK8
Morindae Officinalis Radix	Physcion	MME
Morindae Officinalis Radix	Physcion	MMP1
Morindae Officinalis Radix	Physcion	MMP10
Morindae Officinalis Radix	Physcion	MMP12
Morindae Officinalis Radix	Physcion	MMP2
Morindae Officinalis Radix	Physcion	MMP3
Morindae Officinalis Radix	Physcion	MMP9
Morindae Officinalis Radix	Physcion	NOX4
Morindae Officinalis Radix	Physcion	NQO1
Morindae Officinalis Radix	Physcion	PDE5A
Morindae Officinalis Radix	Physcion	PDGFRA
Morindae Officinalis Radix	Physcion	PDGFRB
Morindae Officinalis Radix	Physcion	PIK3CD
Morindae Officinalis Radix	Physcion	PLA2G7
Morindae Officinalis Radix	Physcion	TERT
Morindae Officinalis Radix	sitosterol	NR3C2
Morindae Officinalis Radix	sitosterol	PGR
Morindae Officinalis Radix	Vitamin C	GSK3B
Panax ginseng C.A. Meyer	(+)-Maalioxide	ACHE
Panax ginseng C.A. Meyer	(+)-Maalioxide	SHH
Panax ginseng C.A. Meyer	13-Tetradecenyl acetate	ACHE
Panax ginseng C.A. Meyer	13-Tetradecenyl acetate	ALOX5AP
Panax ginseng C.A. Meyer	13-Tetradecenyl acetate	APP
Panax ginseng C.A. Meyer	13-Tetradecenyl acetate	ASAH1
Panax ginseng C.A. Meyer	13-Tetradecenyl acetate	BCL2
Panax ginseng C.A. Meyer	13-Tetradecenyl acetate	F2
Panax ginseng C.A. Meyer	13-Tetradecenyl acetate	F2R
Panax ginseng C.A. Meyer	13-Tetradecenyl acetate	FAAH
Panax ginseng C.A. Meyer	13-Tetradecenyl acetate	GRIN1
Panax ginseng C.A. Meyer	13-Tetradecenyl acetate	HSD11B1
Panax ginseng C.A. Meyer	13-Tetradecenyl acetate	MAPT
Panax ginseng C.A. Meyer	13-Tetradecenyl acetate	PREP
Panax ginseng C.A. Meyer	13-Tetradecenyl acetate	PTPN1
Panax ginseng C.A. Meyer	13-Tetradecenyl acetate	ROCK1
Panax ginseng C.A. Meyer	13-Tetradecenyl acetate	ROCK2
Panax ginseng C.A. Meyer	13-Tetradecenyl acetate	SNCA
Panax ginseng C.A. Meyer	13-Tetradecenyl acetate	SRC
Panax ginseng C.A. Meyer	1-heptadecanol	ACHE
Panax ginseng C.A. Meyer	1-heptadecanol	AR
Panax ginseng C.A. Meyer	1-heptadecanol	CA1

Panax ginseng C.A. Meyer	1-heptadecanol	CA4
Panax ginseng C.A. Meyer	1-heptadecanol	CHRM1
Panax ginseng C.A. Meyer	1-heptadecanol	CHRM2
Panax ginseng C.A. Meyer	1-heptadecanol	CNR1
Panax ginseng C.A. Meyer	1-heptadecanol	CNR2
Panax ginseng C.A. Meyer	1-heptadecanol	DNM1
Panax ginseng C.A. Meyer	1-heptadecanol	ESR1
Panax ginseng C.A. Meyer	1-heptadecanol	ESR2
Panax ginseng C.A. Meyer	1-heptadecanol	FAAH
Panax ginseng C.A. Meyer	1-heptadecanol	HSD11B1
Panax ginseng C.A. Meyer	1-heptadecanol	ITGAL
Panax ginseng C.A. Meyer	1-heptadecanol	NR1H4
Panax ginseng C.A. Meyer	1-heptadecanol	RBP4
Panax ginseng C.A. Meyer	1-heptadecanol	SHH
Panax ginseng C.A. Meyer	1-tetradecanol	AR
Panax ginseng C.A. Meyer	1-tetradecanol	CA1
Panax ginseng C.A. Meyer	1-tetradecanol	CA4
Panax ginseng C.A. Meyer	1-tetradecanol	CNR1
Panax ginseng C.A. Meyer	1-tetradecanol	CNR2
Panax ginseng C.A. Meyer	1-tetradecanol	ESR1
Panax ginseng C.A. Meyer	1-tetradecanol	ESR2
Panax ginseng C.A. Meyer	1-tetradecanol	GRM2
Panax ginseng C.A. Meyer	1-tetradecanol	HSD11B1
Panax ginseng C.A. Meyer	1-tetradecanol	NR1H4
Panax ginseng C.A. Meyer	1-tetradecanol	NR3C1
Panax ginseng C.A. Meyer	1-tetradecanol	PRKCA
Panax ginseng C.A. Meyer	1-tetradecanol	RBP4
Panax ginseng C.A. Meyer	1-tetradecanol	SHH
Panax ginseng C.A. Meyer	1-tetradecanol	SLC6A2
Panax ginseng C.A. Meyer	1-tetradecanol	TNF
Panax ginseng C.A. Meyer	2,6-dimethyl-3,7-octadiene-2,6-diol	ABCC9
Panax ginseng C.A. Meyer	2,6-dimethyl-3,7-octadiene-2,6-diol	AOC3
Panax ginseng C.A. Meyer	2,6-dimethyl-3,7-octadiene-2,6-diol	APP
Panax ginseng C.A. Meyer	2,6-dimethyl-3,7-octadiene-2,6-diol	CA4
Panax ginseng C.A. Meyer	2,6-dimethyl-3,7-octadiene-2,6-diol	CDK5R1
Panax ginseng C.A. Meyer	2,6-dimethyl-3,7-octadiene-2,6-diol	CHRM1
Panax ginseng C.A. Meyer	2,6-dimethyl-3,7-octadiene-2,6-diol	CYP11B2

Panax ginseng C.A. Meyer	2,6-dimethyl-3,7-octadiene-2,6-diol	CYP2C19
Panax ginseng C.A. Meyer	2,6-dimethyl-3,7-octadiene-2,6-diol	CYP3A4
Panax ginseng C.A. Meyer	2,6-dimethyl-3,7-octadiene-2,6-diol	DAO
Panax ginseng C.A. Meyer	2,6-dimethyl-3,7-octadiene-2,6-diol	FAAH
Panax ginseng C.A. Meyer	2,6-dimethyl-3,7-octadiene-2,6-diol	GSR
Panax ginseng C.A. Meyer	2,6-dimethyl-3,7-octadiene-2,6-diol	JAK2
Panax ginseng C.A. Meyer	2,6-dimethyl-3,7-octadiene-2,6-diol	LRRK2
Panax ginseng C.A. Meyer	2,6-dimethyl-3,7-octadiene-2,6-diol	MIF
Panax ginseng C.A. Meyer	2,6-dimethyl-3,7-octadiene-2,6-diol	MPO
Panax ginseng C.A. Meyer	2,6-dimethyl-3,7-octadiene-2,6-diol	NR3C1
Panax ginseng C.A. Meyer	2,6-dimethyl-3,7-octadiene-2,6-diol	PARP1
Panax ginseng C.A. Meyer	2,6-dimethyl-3,7-octadiene-2,6-diol	PDE10A
Panax ginseng C.A. Meyer	2,6-dimethyl-3,7-octadiene-2,6-diol	PGR
Panax ginseng C.A. Meyer	2,6-dimethyl-3,7-octadiene-2,6-diol	PIK3CB
Panax ginseng C.A. Meyer	2,6-dimethyl-3,7-octadiene-2,6-diol	PIK3CD
Panax ginseng C.A. Meyer	2,6-dimethyl-3,7-octadiene-2,6-diol	PTGS1
Panax ginseng C.A. Meyer	2,6-dimethyl-3,7-octadiene-2,6-diol	PTGS2
Panax ginseng C.A. Meyer	2,6-dimethyl-3,7-octadiene-2,6-diol	SLC6A3
Panax ginseng C.A. Meyer	20(R)-Ginsenoside Rg3	BCL2L1
Panax ginseng C.A. Meyer	20(R)-Ginsenoside Rg3	CDK1
Panax ginseng C.A. Meyer	20(R)-Ginsenoside Rg3	FGF1
Panax ginseng C.A. Meyer	20(R)-Ginsenoside Rg3	FGF2
Panax ginseng C.A. Meyer	20(R)-Ginsenoside Rg3	HPSE
Panax ginseng C.A. Meyer	20(R)-Ginsenoside Rg3	HSD11B1
Panax ginseng C.A. Meyer	20(R)-Ginsenoside Rg3	HSP90AA1
Panax ginseng C.A. Meyer	20(R)-Ginsenoside Rg3	IL2

Panax ginseng C.A. Meyer	20(R)-Ginsenoside Rg3	LGALS3
Panax ginseng C.A. Meyer	20(R)-Ginsenoside Rg3	PSEN2
Panax ginseng C.A. Meyer	20(R)-Ginsenoside Rg3	STAT3
Panax ginseng C.A. Meyer	20(R)-Ginsenoside Rg3	VEGFA
Panax ginseng C.A. Meyer	20-(R)-Ginsenoside-Rg2	ADORA1
Panax ginseng C.A. Meyer	20-(R)-Ginsenoside-Rg2	ADORA2A
Panax ginseng C.A. Meyer	20-(R)-Ginsenoside-Rg2	EGFR
Panax ginseng C.A. Meyer	20-(R)-Ginsenoside-Rg2	F2
Panax ginseng C.A. Meyer	20-(R)-Ginsenoside-Rg2	FGF1
Panax ginseng C.A. Meyer	20-(R)-Ginsenoside-Rg2	FGF2
Panax ginseng C.A. Meyer	20-(R)-Ginsenoside-Rg2	HPSE
Panax ginseng C.A. Meyer	20-(R)-Ginsenoside-Rg2	HSD11B1
Panax ginseng C.A. Meyer	20-(R)-Ginsenoside-Rg2	HSP90AA1
Panax ginseng C.A. Meyer	20-(R)-Ginsenoside-Rg2	IL2
Panax ginseng C.A. Meyer	20-(R)-Ginsenoside-Rg2	ITGAL
Panax ginseng C.A. Meyer	20-(R)-Ginsenoside-Rg2	MAP2K1
Panax ginseng C.A. Meyer	20-(R)-Ginsenoside-Rg2	MMP2
Panax ginseng C.A. Meyer	20-(R)-Ginsenoside-Rg2	MTOR
Panax ginseng C.A. Meyer	20-(R)-Ginsenoside-Rg2	PDE5A
Panax ginseng C.A. Meyer	20-(R)-Ginsenoside-Rg2	PIK3CA
Panax ginseng C.A. Meyer	20-(R)-Ginsenoside-Rg2	PIK3CB
Panax ginseng C.A. Meyer	20-(R)-Ginsenoside-Rg2	PIK3CD
Panax ginseng C.A. Meyer	20-(R)-Ginsenoside-Rg2	PIK3CG
Panax ginseng C.A. Meyer	20-(R)-Ginsenoside-Rg2	REN
Panax ginseng C.A. Meyer	20-(R)-Ginsenoside-Rg2	STAT3
Panax ginseng C.A. Meyer	20-(R)-Ginsenoside-Rg2	TOP1
Panax ginseng C.A. Meyer	20-(R)-Ginsenoside-Rg2	TYMS
Panax ginseng C.A. Meyer	20-(R)-Ginsenoside-Rg2	VEGFA
Panax ginseng C.A. Meyer	20(s)-protopanaxadiol	ACHE
Panax ginseng C.A. Meyer	20(s)-protopanaxadiol	ADORA1
Panax ginseng C.A. Meyer	20(s)-protopanaxadiol	ADORA2A
Panax ginseng C.A. Meyer	20(s)-protopanaxadiol	ALK
Panax ginseng C.A. Meyer	20(s)-protopanaxadiol	AR
Panax ginseng C.A. Meyer	20(s)-protopanaxadiol	BCHE
Panax ginseng C.A. Meyer	20(s)-protopanaxadiol	CCR1
Panax ginseng C.A. Meyer	20(s)-protopanaxadiol	CDK4
Panax ginseng C.A. Meyer	20(s)-protopanaxadiol	CHRM2
Panax ginseng C.A. Meyer	20(s)-protopanaxadiol	CYP2C19
Panax ginseng C.A. Meyer	20(s)-protopanaxadiol	CYP2C9
Panax ginseng C.A. Meyer	20(s)-protopanaxadiol	CYP3A4
Panax ginseng C.A. Meyer	20(s)-protopanaxadiol	ESR1
Panax ginseng C.A. Meyer	20(s)-protopanaxadiol	ESR2
Panax ginseng C.A. Meyer	20(s)-protopanaxadiol	HMGCR
Panax ginseng C.A. Meyer	20(s)-protopanaxadiol	HSD11B1

Panax ginseng C.A. Meyer	20(s)-protopanaxadiol	MAPK14
Panax ginseng C.A. Meyer	20(s)-protopanaxadiol	MAPK8
Panax ginseng C.A. Meyer	20(s)-protopanaxadiol	MDM2
Panax ginseng C.A. Meyer	20(s)-protopanaxadiol	NR1H4
Panax ginseng C.A. Meyer	20(s)-protopanaxadiol	PDE10A
Panax ginseng C.A. Meyer	20(s)-protopanaxadiol	PDE2A
Panax ginseng C.A. Meyer	20(s)-protopanaxadiol	PRKCB
Panax ginseng C.A. Meyer	20(s)-protopanaxadiol	PRKCD
Panax ginseng C.A. Meyer	20(s)-protopanaxadiol	PRKCE
Panax ginseng C.A. Meyer	20(s)-protopanaxadiol	PRKCG
Panax ginseng C.A. Meyer	20(s)-protopanaxadiol	PTPN1
Panax ginseng C.A. Meyer	20(s)-protopanaxadiol	SLC6A2
Panax ginseng C.A. Meyer	20(s)-protopanaxadiol	SLC6A4
Panax ginseng C.A. Meyer	20(s)-protopanaxadiol	VDR
Panax ginseng C.A. Meyer	2-Formylpyrrole	ACHE
Panax ginseng C.A. Meyer	2-Formylpyrrole	BCHE
Panax ginseng C.A. Meyer	2-Formylpyrrole	IDO1
Panax ginseng C.A. Meyer	2-Formylpyrrole	PTPN1
Panax ginseng C.A. Meyer	2-Formylpyrrole	PTPRC
Panax ginseng C.A. Meyer	2-Heptadecanone	AR
Panax ginseng C.A. Meyer	2-Heptadecanone	CA1
Panax ginseng C.A. Meyer	2-Heptadecanone	CA4
Panax ginseng C.A. Meyer	2-Heptadecanone	CNR1
Panax ginseng C.A. Meyer	2-Heptadecanone	CNR2
Panax ginseng C.A. Meyer	2-Heptadecanone	PDE3A
Panax ginseng C.A. Meyer	2-Heptadecanone	PLAU
Panax ginseng C.A. Meyer	2-Heptadecanone	ROCK1
Panax ginseng C.A. Meyer	2-Heptadecanone	ROCK2
Panax ginseng C.A. Meyer	2-Heptadecanone	TGM2
Panax ginseng C.A. Meyer	3,5-dimethyl-4-methoxybenzoic acid	CA1
Panax ginseng C.A. Meyer	3,5-dimethyl-4-methoxybenzoic acid	CA4
Panax ginseng C.A. Meyer	3,5-dimethyl-4-methoxybenzoic acid	CHRNA4
Panax ginseng C.A. Meyer	3,5-dimethyl-4-methoxybenzoic acid	ERN1
Panax ginseng C.A. Meyer	3,5-dimethyl-4-methoxybenzoic acid	ESR2
Panax ginseng C.A. Meyer	3,5-dimethyl-4-methoxybenzoic acid	MAOB
Panax ginseng C.A. Meyer	3,5-dimethyl-4-methoxybenzoic acid	PTGS2
Panax ginseng C.A. Meyer	9-HEXADECENOIC ACID	ACHE

Panax ginseng C.A. Meyer	9-HEXADECENOIC ACID	ACP1
Panax ginseng C.A. Meyer	9-HEXADECENOIC ACID	ALOX12
Panax ginseng C.A. Meyer	9-HEXADECENOIC ACID	ALOX5
Panax ginseng C.A. Meyer	9-HEXADECENOIC ACID	AR
Panax ginseng C.A. Meyer	9-HEXADECENOIC ACID	BACE1
Panax ginseng C.A. Meyer	9-HEXADECENOIC ACID	BCHE
Panax ginseng C.A. Meyer	9-HEXADECENOIC ACID	CHRM2
Panax ginseng C.A. Meyer	9-HEXADECENOIC ACID	CNR1
Panax ginseng C.A. Meyer	9-HEXADECENOIC ACID	CYP2C19
Panax ginseng C.A. Meyer	9-HEXADECENOIC ACID	ESR1
Panax ginseng C.A. Meyer	9-HEXADECENOIC ACID	ESR2
Panax ginseng C.A. Meyer	9-HEXADECENOIC ACID	FAAH
Panax ginseng C.A. Meyer	9-HEXADECENOIC ACID	FABP3
Panax ginseng C.A. Meyer	9-HEXADECENOIC ACID	G6PD
Panax ginseng C.A. Meyer	9-HEXADECENOIC ACID	HMGCR
Panax ginseng C.A. Meyer	9-HEXADECENOIC ACID	HSD11B1
Panax ginseng C.A. Meyer	9-HEXADECENOIC ACID	IDO1
Panax ginseng C.A. Meyer	9-HEXADECENOIC ACID	ITGAL
Panax ginseng C.A. Meyer	9-HEXADECENOIC ACID	MAPK3
Panax ginseng C.A. Meyer	9-HEXADECENOIC ACID	NOS2
Panax ginseng C.A. Meyer	9-HEXADECENOIC ACID	NR1H4
Panax ginseng C.A. Meyer	9-HEXADECENOIC ACID	NR3C1
Panax ginseng C.A. Meyer	9-HEXADECENOIC ACID	PGR
Panax ginseng C.A. Meyer	9-HEXADECENOIC ACID	PLA2G1B
Panax ginseng C.A. Meyer	9-HEXADECENOIC ACID	PPARA
Panax ginseng C.A. Meyer	9-HEXADECENOIC ACID	PPARD
Panax ginseng C.A. Meyer	9-HEXADECENOIC ACID	PPARG
Panax ginseng C.A. Meyer	9-HEXADECENOIC ACID	PREP
Panax ginseng C.A. Meyer	9-HEXADECENOIC ACID	PRKCH
Panax ginseng C.A. Meyer	9-HEXADECENOIC ACID	PTGIR
Panax ginseng C.A. Meyer	9-HEXADECENOIC ACID	PTGS1
Panax ginseng C.A. Meyer	9-HEXADECENOIC ACID	PTGS2
Panax ginseng C.A. Meyer	9-HEXADECENOIC ACID	PTPN1
Panax ginseng C.A. Meyer	9-HEXADECENOIC ACID	PTPN11
Panax ginseng C.A. Meyer	9-HEXADECENOIC ACID	PTPN6
Panax ginseng C.A. Meyer	9-HEXADECENOIC ACID	SIGMAR1
Panax ginseng C.A. Meyer	9-HEXADECENOIC ACID	SLC6A2
Panax ginseng C.A. Meyer	9-HEXADECENOIC ACID	SLC6A3
Panax ginseng C.A. Meyer	9-HEXADECENOIC ACID	SLC6A4
Panax ginseng C.A. Meyer	9-HEXADECENOIC ACID	TERT
Panax ginseng C.A. Meyer	9-HEXADECENOIC ACID	TOP1
Panax ginseng C.A. Meyer	adenine	ADORA2A
Panax ginseng C.A. Meyer	Alpha-Cadinol	ACHE
Panax ginseng C.A. Meyer	Alpha-Cadinol	ACP1

Panax ginseng C.A. Meyer	Alpha-Cadinol	AR
Panax ginseng C.A. Meyer	Alpha-Cadinol	BCHE
Panax ginseng C.A. Meyer	Alpha-Cadinol	CHRM2
Panax ginseng C.A. Meyer	Alpha-Cadinol	CYP2C19
Panax ginseng C.A. Meyer	Alpha-Cadinol	ESR1
Panax ginseng C.A. Meyer	Alpha-Cadinol	ESR2
Panax ginseng C.A. Meyer	Alpha-Cadinol	FABP3
Panax ginseng C.A. Meyer	Alpha-Cadinol	G6PD
Panax ginseng C.A. Meyer	Alpha-Cadinol	HMGCR
Panax ginseng C.A. Meyer	Alpha-Cadinol	HSD11B1
Panax ginseng C.A. Meyer	Alpha-Cadinol	PLA2G1B
Panax ginseng C.A. Meyer	Alpha-Cadinol	PPARA
Panax ginseng C.A. Meyer	Alpha-Cadinol	PPARD
Panax ginseng C.A. Meyer	Alpha-Cadinol	PREP
Panax ginseng C.A. Meyer	Alpha-Cadinol	PTPN1
Panax ginseng C.A. Meyer	Alpha-Cadinol	PTPN11
Panax ginseng C.A. Meyer	Alpha-Cadinol	PTPN6
Panax ginseng C.A. Meyer	Alpha-Cadinol	SLC6A2
Panax ginseng C.A. Meyer	Alpha-Cadinol	SLC6A4
Panax ginseng C.A. Meyer	Alpha-Cadinol	SREBF2
Panax ginseng C.A. Meyer	Alpha-Cadinol	TERT
Panax ginseng C.A. Meyer	alpha-Guttiferin	ABCB1
Panax ginseng C.A. Meyer	alpha-Guttiferin	CXCR2
Panax ginseng C.A. Meyer	alpha-Guttiferin	PRKCB
Panax ginseng C.A. Meyer	alpha-Guttiferin	PRKCD
Panax ginseng C.A. Meyer	alpha-Guttiferin	PRKCE
Panax ginseng C.A. Meyer	alpha-santalol	ACHE
Panax ginseng C.A. Meyer	alpha-santalol	AR
Panax ginseng C.A. Meyer	alpha-santalol	CHRM2
Panax ginseng C.A. Meyer	alpha-santalol	CYP2C19
Panax ginseng C.A. Meyer	alpha-santalol	ESR1
Panax ginseng C.A. Meyer	alpha-santalol	IDO1
Panax ginseng C.A. Meyer	alpha-santalol	PSEN2
Panax ginseng C.A. Meyer	alpha-santalol	SLC6A2
Panax ginseng C.A. Meyer	alpha-santalol	SLC6A4
Panax ginseng C.A. Meyer	anethole	AR
Panax ginseng C.A. Meyer	anethole	DYRK1A
Panax ginseng C.A. Meyer	anethole	IDO1
Panax ginseng C.A. Meyer	anethole	MAOA
Panax ginseng C.A. Meyer	anethole	MAOB
Panax ginseng C.A. Meyer	anethole	NQO1
Panax ginseng C.A. Meyer	anethole	PTGS1
Panax ginseng C.A. Meyer	anethole	PTGS2
Panax ginseng C.A. Meyer	anisaldehyde	ACHE

Panax ginseng C.A. Meyer	anisaldehyde	CA1
Panax ginseng C.A. Meyer	anisaldehyde	CA4
Panax ginseng C.A. Meyer	anisaldehyde	CNR1
Panax ginseng C.A. Meyer	anisaldehyde	CNR2
Panax ginseng C.A. Meyer	anisaldehyde	ERN1
Panax ginseng C.A. Meyer	anisaldehyde	GSK3B
Panax ginseng C.A. Meyer	anisaldehyde	TGM2
Panax ginseng C.A. Meyer	aposcopolamine	ACHE
Panax ginseng C.A. Meyer	aposcopolamine	ADORA1
Panax ginseng C.A. Meyer	aposcopolamine	ADORA2A
Panax ginseng C.A. Meyer	aposcopolamine	ALOX5
Panax ginseng C.A. Meyer	aposcopolamine	APP
Panax ginseng C.A. Meyer	aposcopolamine	AR
Panax ginseng C.A. Meyer	aposcopolamine	ATM
Panax ginseng C.A. Meyer	aposcopolamine	BDKRB2
Panax ginseng C.A. Meyer	aposcopolamine	CA1
Panax ginseng C.A. Meyer	aposcopolamine	CA4
Panax ginseng C.A. Meyer	aposcopolamine	CDK1
Panax ginseng C.A. Meyer	aposcopolamine	CHRM1
Panax ginseng C.A. Meyer	aposcopolamine	CHRM2
Panax ginseng C.A. Meyer	aposcopolamine	CYP11B2
Panax ginseng C.A. Meyer	aposcopolamine	DRD1
Panax ginseng C.A. Meyer	aposcopolamine	ESR2
Panax ginseng C.A. Meyer	aposcopolamine	GRM1
Panax ginseng C.A. Meyer	aposcopolamine	GSK3B
Panax ginseng C.A. Meyer	aposcopolamine	HIF1A
Panax ginseng C.A. Meyer	aposcopolamine	HSD11B1
Panax ginseng C.A. Meyer	aposcopolamine	HTR2C
Panax ginseng C.A. Meyer	aposcopolamine	JAK2
Panax ginseng C.A. Meyer	aposcopolamine	KCNMA1
Panax ginseng C.A. Meyer	aposcopolamine	MAPK14
Panax ginseng C.A. Meyer	aposcopolamine	MIF
Panax ginseng C.A. Meyer	aposcopolamine	MPO
Panax ginseng C.A. Meyer	aposcopolamine	NTRK1
Panax ginseng C.A. Meyer	aposcopolamine	NTRK2
Panax ginseng C.A. Meyer	aposcopolamine	NTRK3
Panax ginseng C.A. Meyer	aposcopolamine	P2RX7
Panax ginseng C.A. Meyer	aposcopolamine	PDE10A
Panax ginseng C.A. Meyer	aposcopolamine	PIK3CA
Panax ginseng C.A. Meyer	aposcopolamine	PIK3CB
Panax ginseng C.A. Meyer	aposcopolamine	PIK3CD
Panax ginseng C.A. Meyer	aposcopolamine	PIK3CG
Panax ginseng C.A. Meyer	aposcopolamine	PLA2G7
Panax ginseng C.A. Meyer	aposcopolamine	PSEN1

Panax ginseng C.A. Meyer	aposcopolamine	PSEN2
Panax ginseng C.A. Meyer	aposcopolamine	SCN9A
Panax ginseng C.A. Meyer	aposcopolamine	SIGMAR1
Panax ginseng C.A. Meyer	aposcopolamine	SLC1A3
Panax ginseng C.A. Meyer	aposcopolamine	SLC6A3
Panax ginseng C.A. Meyer	aposcopolamine	SLC6A4
Panax ginseng C.A. Meyer	aposcopolamine	TERT
Panax ginseng C.A. Meyer	aposcopolamine	TGFBR1
Panax ginseng C.A. Meyer	arachidonate	ACE
Panax ginseng C.A. Meyer	arachidonate	ADRA2B
Panax ginseng C.A. Meyer	arachidonate	AGTR1
Panax ginseng C.A. Meyer	arachidonate	AKR1B1
Panax ginseng C.A. Meyer	arachidonate	ALOX12
Panax ginseng C.A. Meyer	arachidonate	ALOX5
Panax ginseng C.A. Meyer	arachidonate	ALOX5AP
Panax ginseng C.A. Meyer	arachidonate	CMA1
Panax ginseng C.A. Meyer	arachidonate	CNR1
Panax ginseng C.A. Meyer	arachidonate	CNR2
Panax ginseng C.A. Meyer	arachidonate	ESR1
Panax ginseng C.A. Meyer	arachidonate	ESR2
Panax ginseng C.A. Meyer	arachidonate	FAAH
Panax ginseng C.A. Meyer	arachidonate	FABP3
Panax ginseng C.A. Meyer	arachidonate	HMGCR
Panax ginseng C.A. Meyer	arachidonate	HSD11B1
Panax ginseng C.A. Meyer	arachidonate	ITGAL
Panax ginseng C.A. Meyer	arachidonate	KEAP1
Panax ginseng C.A. Meyer	arachidonate	MAPK1
Panax ginseng C.A. Meyer	arachidonate	MAPK14
Panax ginseng C.A. Meyer	arachidonate	MDM2
Panax ginseng C.A. Meyer	arachidonate	MMP12
Panax ginseng C.A. Meyer	arachidonate	MMP2
Panax ginseng C.A. Meyer	arachidonate	MMP3
Panax ginseng C.A. Meyer	arachidonate	MMP9
Panax ginseng C.A. Meyer	arachidonate	NOS2
Panax ginseng C.A. Meyer	arachidonate	NR3C1
Panax ginseng C.A. Meyer	arachidonate	NR3C2
Panax ginseng C.A. Meyer	arachidonate	PDE4A
Panax ginseng C.A. Meyer	arachidonate	PGR
Panax ginseng C.A. Meyer	arachidonate	PLA2G4A
Panax ginseng C.A. Meyer	arachidonate	PPARA
Panax ginseng C.A. Meyer	arachidonate	PPARD
Panax ginseng C.A. Meyer	arachidonate	PPARG
Panax ginseng C.A. Meyer	arachidonate	PRKCH
Panax ginseng C.A. Meyer	arachidonate	PSEN2

Panax ginseng C.A. Meyer	arachidonate	PTGIR
Panax ginseng C.A. Meyer	arachidonate	PTGS1
Panax ginseng C.A. Meyer	arachidonate	PTGS2
Panax ginseng C.A. Meyer	arachidonate	PTPN1
Panax ginseng C.A. Meyer	arachidonate	PTPN11
Panax ginseng C.A. Meyer	arachidonate	PTPN6
Panax ginseng C.A. Meyer	arachidonate	RARA
Panax ginseng C.A. Meyer	arachidonate	RBP4
Panax ginseng C.A. Meyer	arachidonate	RXRA
Panax ginseng C.A. Meyer	arachidonate	TERT
Panax ginseng C.A. Meyer	arachidonate	TOP1
Panax ginseng C.A. Meyer	arachidonate	TP53
Panax ginseng C.A. Meyer	beta-santalol	ACHE
Panax ginseng C.A. Meyer	beta-santalol	AR
Panax ginseng C.A. Meyer	beta-santalol	CHRM1
Panax ginseng C.A. Meyer	beta-santalol	CHRM2
Panax ginseng C.A. Meyer	beta-santalol	CYP2C19
Panax ginseng C.A. Meyer	beta-santalol	ESR1
Panax ginseng C.A. Meyer	beta-santalol	IDO1
Panax ginseng C.A. Meyer	beta-santalol	JAK2
Panax ginseng C.A. Meyer	beta-santalol	PIK3CA
Panax ginseng C.A. Meyer	beta-santalol	PIK3CG
Panax ginseng C.A. Meyer	beta-santalol	PSEN2
Panax ginseng C.A. Meyer	beta-santalol	SLC18A3
Panax ginseng C.A. Meyer	beta-santalol	SLC6A2
Panax ginseng C.A. Meyer	beta-santalol	SLC6A4
Panax ginseng C.A. Meyer	Betulafolienetriol	ACHE
Panax ginseng C.A. Meyer	Betulafolienetriol	ADORA1
Panax ginseng C.A. Meyer	Betulafolienetriol	ADORA2A
Panax ginseng C.A. Meyer	Betulafolienetriol	ALK
Panax ginseng C.A. Meyer	Betulafolienetriol	AR
Panax ginseng C.A. Meyer	Betulafolienetriol	BCHE
Panax ginseng C.A. Meyer	Betulafolienetriol	CAPN2
Panax ginseng C.A. Meyer	Betulafolienetriol	CHRM2
Panax ginseng C.A. Meyer	Betulafolienetriol	CYP2C19
Panax ginseng C.A. Meyer	Betulafolienetriol	CYP2C9
Panax ginseng C.A. Meyer	Betulafolienetriol	CYP3A4
Panax ginseng C.A. Meyer	Betulafolienetriol	ESR1
Panax ginseng C.A. Meyer	Betulafolienetriol	F2R
Panax ginseng C.A. Meyer	Betulafolienetriol	HMGCR
Panax ginseng C.A. Meyer	Betulafolienetriol	HSD11B1
Panax ginseng C.A. Meyer	Betulafolienetriol	INSR
Panax ginseng C.A. Meyer	Betulafolienetriol	KDR
Panax ginseng C.A. Meyer	Betulafolienetriol	MAPK14

Panax ginseng C.A. Meyer	Betulafolienetriol	MAPK8
Panax ginseng C.A. Meyer	Betulafolienetriol	MDM2
Panax ginseng C.A. Meyer	Betulafolienetriol	PDE10A
Panax ginseng C.A. Meyer	Betulafolienetriol	PDE2A
Panax ginseng C.A. Meyer	Betulafolienetriol	PIK3CA
Panax ginseng C.A. Meyer	Betulafolienetriol	PIK3CB
Panax ginseng C.A. Meyer	Betulafolienetriol	PIK3CD
Panax ginseng C.A. Meyer	Betulafolienetriol	PIK3CG
Panax ginseng C.A. Meyer	Betulafolienetriol	PTGS1
Panax ginseng C.A. Meyer	Betulafolienetriol	PTGS2
Panax ginseng C.A. Meyer	Betulafolienetriol	PTPN1
Panax ginseng C.A. Meyer	Betulafolienetriol	SLC6A2
Panax ginseng C.A. Meyer	Betulafolienetriol	SLC6A4
Panax ginseng C.A. Meyer	Betulafolienetriol	VDR
Panax ginseng C.A. Meyer	Biotin	ACE2
Panax ginseng C.A. Meyer	Biotin	ADORA1
Panax ginseng C.A. Meyer	Biotin	AKR1B1
Panax ginseng C.A. Meyer	Biotin	APEX1
Panax ginseng C.A. Meyer	Biotin	CA1
Panax ginseng C.A. Meyer	Biotin	CAPN1
Panax ginseng C.A. Meyer	Biotin	CHRM1
Panax ginseng C.A. Meyer	Biotin	CNR1
Panax ginseng C.A. Meyer	Biotin	CREBBP
Panax ginseng C.A. Meyer	Biotin	CTSA
Panax ginseng C.A. Meyer	Biotin	CXCL8
Panax ginseng C.A. Meyer	Biotin	CXCR2
Panax ginseng C.A. Meyer	Biotin	DAO
Panax ginseng C.A. Meyer	Biotin	DYRK1A
Panax ginseng C.A. Meyer	Biotin	ECE1
Panax ginseng C.A. Meyer	Biotin	EDNRA
Panax ginseng C.A. Meyer	Biotin	EGFR
Panax ginseng C.A. Meyer	Biotin	FOLH1
Panax ginseng C.A. Meyer	Biotin	GRK2
Panax ginseng C.A. Meyer	Biotin	HSP90AA1
Panax ginseng C.A. Meyer	Biotin	HSPA1A
Panax ginseng C.A. Meyer	Biotin	IDE
Panax ginseng C.A. Meyer	Biotin	ITGAL
Panax ginseng C.A. Meyer	Biotin	ITGAV
Panax ginseng C.A. Meyer	Biotin	ITGB1
Panax ginseng C.A. Meyer	Biotin	MAOB
Panax ginseng C.A. Meyer	Biotin	MAPK10
Panax ginseng C.A. Meyer	Biotin	MIF
Panax ginseng C.A. Meyer	Biotin	MMP12
Panax ginseng C.A. Meyer	Biotin	PARP1

Panax ginseng C.A. Meyer	Biotin	PDE4A
Panax ginseng C.A. Meyer	Biotin	PDE5A
Panax ginseng C.A. Meyer	Biotin	PLA2G2A
Panax ginseng C.A. Meyer	Biotin	PPARA
Panax ginseng C.A. Meyer	Biotin	PPARG
Panax ginseng C.A. Meyer	Biotin	PTGS1
Panax ginseng C.A. Meyer	Biotin	PTGS2
Panax ginseng C.A. Meyer	Biotin	PTPRC
Panax ginseng C.A. Meyer	Biotin	SLC6A4
Panax ginseng C.A. Meyer	Biotin	TTR
Panax ginseng C.A. Meyer	calarene	ACE2
Panax ginseng C.A. Meyer	calarene	ADORA1
Panax ginseng C.A. Meyer	calarene	AKR1B1
Panax ginseng C.A. Meyer	calarene	APEX1
Panax ginseng C.A. Meyer	calarene	CA1
Panax ginseng C.A. Meyer	calarene	CAPN1
Panax ginseng C.A. Meyer	calarene	CHRM1
Panax ginseng C.A. Meyer	calarene	CNR1
Panax ginseng C.A. Meyer	calarene	CREBBP
Panax ginseng C.A. Meyer	calarene	CTSA
Panax ginseng C.A. Meyer	calarene	CXCL8
Panax ginseng C.A. Meyer	calarene	CXCR2
Panax ginseng C.A. Meyer	calarene	DAO
Panax ginseng C.A. Meyer	calarene	DYRK1A
Panax ginseng C.A. Meyer	calarene	ECE1
Panax ginseng C.A. Meyer	calarene	EDNRA
Panax ginseng C.A. Meyer	calarene	EGFR
Panax ginseng C.A. Meyer	calarene	FOLH1
Panax ginseng C.A. Meyer	calarene	GRK2
Panax ginseng C.A. Meyer	calarene	HSP90AA1
Panax ginseng C.A. Meyer	calarene	HSPA1A
Panax ginseng C.A. Meyer	calarene	IDE
Panax ginseng C.A. Meyer	calarene	ITGAL
Panax ginseng C.A. Meyer	calarene	ITGAV
Panax ginseng C.A. Meyer	calarene	ITGB1
Panax ginseng C.A. Meyer	calarene	MAOB
Panax ginseng C.A. Meyer	calarene	MAPK10
Panax ginseng C.A. Meyer	calarene	MIF
Panax ginseng C.A. Meyer	calarene	MMP12
Panax ginseng C.A. Meyer	calarene	PARP1
Panax ginseng C.A. Meyer	calarene	PDE4A
Panax ginseng C.A. Meyer	calarene	PDE5A
Panax ginseng C.A. Meyer	calarene	PLA2G2A
Panax ginseng C.A. Meyer	calarene	PPARA

Panax ginseng C.A. Meyer	calarene	PPARG
Panax ginseng C.A. Meyer	calarene	PTGS1
Panax ginseng C.A. Meyer	calarene	PTGS2
Panax ginseng C.A. Meyer	calarene	PTPRC
Panax ginseng C.A. Meyer	calarene	SLC6A4
Panax ginseng C.A. Meyer	calarene	TTR
Panax ginseng C.A. Meyer	chikusetsusaponin III	ABCC1
Panax ginseng C.A. Meyer	chikusetsusaponin III	ACHE
Panax ginseng C.A. Meyer	chikusetsusaponin III	ADORA1
Panax ginseng C.A. Meyer	chikusetsusaponin III	ADRA1A
Panax ginseng C.A. Meyer	chikusetsusaponin III	APP
Panax ginseng C.A. Meyer	chikusetsusaponin III	CAPN1
Panax ginseng C.A. Meyer	chikusetsusaponin III	CCR1
Panax ginseng C.A. Meyer	chikusetsusaponin III	CCR3
Panax ginseng C.A. Meyer	chikusetsusaponin III	CCR5
Panax ginseng C.A. Meyer	chikusetsusaponin III	CHRM1
Panax ginseng C.A. Meyer	chikusetsusaponin III	CHRM2
Panax ginseng C.A. Meyer	chikusetsusaponin III	CHRNA4
Panax ginseng C.A. Meyer	chikusetsusaponin III	CREBBP
Panax ginseng C.A. Meyer	chikusetsusaponin III	CRHR1
Panax ginseng C.A. Meyer	chikusetsusaponin III	CTSB
Panax ginseng C.A. Meyer	chikusetsusaponin III	CYP11B2
Panax ginseng C.A. Meyer	chikusetsusaponin III	CYP27A1
Panax ginseng C.A. Meyer	chikusetsusaponin III	DPP4
Panax ginseng C.A. Meyer	chikusetsusaponin III	DRD2
Panax ginseng C.A. Meyer	chikusetsusaponin III	DRD3
Panax ginseng C.A. Meyer	chikusetsusaponin III	EGFR
Panax ginseng C.A. Meyer	chikusetsusaponin III	EP300
Panax ginseng C.A. Meyer	chikusetsusaponin III	F10
Panax ginseng C.A. Meyer	chikusetsusaponin III	F2
Panax ginseng C.A. Meyer	chikusetsusaponin III	F7
Panax ginseng C.A. Meyer	chikusetsusaponin III	GRM1
Panax ginseng C.A. Meyer	chikusetsusaponin III	HSD11B1
Panax ginseng C.A. Meyer	chikusetsusaponin III	HTR2A
Panax ginseng C.A. Meyer	chikusetsusaponin III	ITGA2B
Panax ginseng C.A. Meyer	chikusetsusaponin III	MAPK14
Panax ginseng C.A. Meyer	chikusetsusaponin III	MAPK8
Panax ginseng C.A. Meyer	chikusetsusaponin III	MTOR
Panax ginseng C.A. Meyer	chikusetsusaponin III	NTRK1
Panax ginseng C.A. Meyer	chikusetsusaponin III	OPRM1
Panax ginseng C.A. Meyer	chikusetsusaponin III	P2RX7
Panax ginseng C.A. Meyer	chikusetsusaponin III	PDE10A
Panax ginseng C.A. Meyer	chikusetsusaponin III	PDE5A
Panax ginseng C.A. Meyer	chikusetsusaponin III	PIK3CA

Panax ginseng C.A. Meyer	chikusetsusaponin III	PLA2G2A
Panax ginseng C.A. Meyer	chikusetsusaponin III	PPIA
Panax ginseng C.A. Meyer	chikusetsusaponin III	SIGMAR1
Panax ginseng C.A. Meyer	chikusetsusaponin III	SLC6A2
Panax ginseng C.A. Meyer	chikusetsusaponin III	SLC6A3
Panax ginseng C.A. Meyer	chikusetsusaponin III	SLC6A4
Panax ginseng C.A. Meyer	chikusetsusaponin III	SYK
Panax ginseng C.A. Meyer	chikusetsusaponin III	TACR1
Panax ginseng C.A. Meyer	chikusetsusaponin III	TGFBR1
Panax ginseng C.A. Meyer	chloropanaxydiol	ABL1
Panax ginseng C.A. Meyer	chloropanaxydiol	ADORA1
Panax ginseng C.A. Meyer	chloropanaxydiol	ADORA2A
Panax ginseng C.A. Meyer	chloropanaxydiol	AKT1
Panax ginseng C.A. Meyer	chloropanaxydiol	ALK
Panax ginseng C.A. Meyer	chloropanaxydiol	ALOX5
Panax ginseng C.A. Meyer	chloropanaxydiol	APP
Panax ginseng C.A. Meyer	chloropanaxydiol	CASP3
Panax ginseng C.A. Meyer	chloropanaxydiol	CBFB
Panax ginseng C.A. Meyer	chloropanaxydiol	CCNA2
Panax ginseng C.A. Meyer	chloropanaxydiol	CDK1
Panax ginseng C.A. Meyer	chloropanaxydiol	CDK2
Panax ginseng C.A. Meyer	chloropanaxydiol	CDK5R1
Panax ginseng C.A. Meyer	chloropanaxydiol	CHRM1
Panax ginseng C.A. Meyer	chloropanaxydiol	CNR1
Panax ginseng C.A. Meyer	chloropanaxydiol	CNR2
Panax ginseng C.A. Meyer	chloropanaxydiol	CRHR1
Panax ginseng C.A. Meyer	chloropanaxydiol	CSF1R
Panax ginseng C.A. Meyer	chloropanaxydiol	CTSB
Panax ginseng C.A. Meyer	chloropanaxydiol	CTSD
Panax ginseng C.A. Meyer	chloropanaxydiol	CTSL
Panax ginseng C.A. Meyer	chloropanaxydiol	CXCR2
Panax ginseng C.A. Meyer	chloropanaxydiol	EGFR
Panax ginseng C.A. Meyer	chloropanaxydiol	FLT4
Panax ginseng C.A. Meyer	chloropanaxydiol	HIF1A
Panax ginseng C.A. Meyer	chloropanaxydiol	HSP90AA1
Panax ginseng C.A. Meyer	chloropanaxydiol	ICAM1
Panax ginseng C.A. Meyer	chloropanaxydiol	JAK2
Panax ginseng C.A. Meyer	chloropanaxydiol	MAP3K14
Panax ginseng C.A. Meyer	chloropanaxydiol	MAP3K5
Panax ginseng C.A. Meyer	chloropanaxydiol	MAPK1
Panax ginseng C.A. Meyer	chloropanaxydiol	MAPK14
Panax ginseng C.A. Meyer	chloropanaxydiol	MAPK8
Panax ginseng C.A. Meyer	chloropanaxydiol	MTOR
Panax ginseng C.A. Meyer	chloropanaxydiol	NTRK1

Panax ginseng C.A. Meyer	chloropanaxydiol	NTRK2
Panax ginseng C.A. Meyer	chloropanaxydiol	NTRK3
Panax ginseng C.A. Meyer	chloropanaxydiol	P2RY12
Panax ginseng C.A. Meyer	chloropanaxydiol	PDE10A
Panax ginseng C.A. Meyer	chloropanaxydiol	PDE5A
Panax ginseng C.A. Meyer	chloropanaxydiol	PDGFRA
Panax ginseng C.A. Meyer	chloropanaxydiol	PIK3CA
Panax ginseng C.A. Meyer	chloropanaxydiol	PRKCA
Panax ginseng C.A. Meyer	chloropanaxydiol	PTK2
Panax ginseng C.A. Meyer	chloropanaxydiol	PTK2B
Panax ginseng C.A. Meyer	chloropanaxydiol	SCN9A
Panax ginseng C.A. Meyer	chloropanaxydiol	SELE
Panax ginseng C.A. Meyer	chloropanaxydiol	SYK
Panax ginseng C.A. Meyer	chloropanaxydiol	TBK1
Panax ginseng C.A. Meyer	citronellal	ACE
Panax ginseng C.A. Meyer	citronellal	ADH1C
Panax ginseng C.A. Meyer	citronellal	AR
Panax ginseng C.A. Meyer	citronellal	CCND1
Panax ginseng C.A. Meyer	citronellal	CHRM1
Panax ginseng C.A. Meyer	citronellal	CTSB
Panax ginseng C.A. Meyer	citronellal	CTSD
Panax ginseng C.A. Meyer	citronellal	CTSL
Panax ginseng C.A. Meyer	citronellal	DRD2
Panax ginseng C.A. Meyer	citronellal	FAAH
Panax ginseng C.A. Meyer	citronellal	FABP3
Panax ginseng C.A. Meyer	citronellal	HDAC1
Panax ginseng C.A. Meyer	citronellal	HDAC6
Panax ginseng C.A. Meyer	citronellal	JAK2
Panax ginseng C.A. Meyer	citronellal	MAOA
Panax ginseng C.A. Meyer	citronellal	MAOB
Panax ginseng C.A. Meyer	citronellal	MMP1
Panax ginseng C.A. Meyer	citronellal	PARP1
Panax ginseng C.A. Meyer	citronellal	PGR
Panax ginseng C.A. Meyer	citronellal	PPARA
Panax ginseng C.A. Meyer	citronellal	PPARD
Panax ginseng C.A. Meyer	citronellal	PPARG
Panax ginseng C.A. Meyer	citronellal	PSEN2
Panax ginseng C.A. Meyer	citronellal	PTGS1
Panax ginseng C.A. Meyer	citronellal	SIGMAR1
Panax ginseng C.A. Meyer	darutoside	ADORA1
Panax ginseng C.A. Meyer	darutoside	BCL2L1
Panax ginseng C.A. Meyer	darutoside	CDK2
Panax ginseng C.A. Meyer	darutoside	DNTT
Panax ginseng C.A. Meyer	darutoside	FGF1

Panax ginseng C.A. Meyer	darutoside	FGF2
Panax ginseng C.A. Meyer	darutoside	GSK3B
Panax ginseng C.A. Meyer	darutoside	HPSE
Panax ginseng C.A. Meyer	darutoside	HSD11B1
Panax ginseng C.A. Meyer	darutoside	IL2
Panax ginseng C.A. Meyer	darutoside	MAP3K14
Panax ginseng C.A. Meyer	darutoside	MTOR
Panax ginseng C.A. Meyer	darutoside	NTRK1
Panax ginseng C.A. Meyer	darutoside	PIK3CA
Panax ginseng C.A. Meyer	darutoside	PTPN1
Panax ginseng C.A. Meyer	darutoside	SELE
Panax ginseng C.A. Meyer	darutoside	SELP
Panax ginseng C.A. Meyer	darutoside	STAT3
Panax ginseng C.A. Meyer	darutoside	TYMS
Panax ginseng C.A. Meyer	darutoside	VEGFA
Panax ginseng C.A. Meyer	Dauricine	ABCB1
Panax ginseng C.A. Meyer	Dauricine	ABL1
Panax ginseng C.A. Meyer	Dauricine	ACHE
Panax ginseng C.A. Meyer	Dauricine	ADRA1A
Panax ginseng C.A. Meyer	Dauricine	ADRA2A
Panax ginseng C.A. Meyer	Dauricine	ADRB2
Panax ginseng C.A. Meyer	Dauricine	ADRB3
Panax ginseng C.A. Meyer	Dauricine	AKT2
Panax ginseng C.A. Meyer	Dauricine	BCHE
Panax ginseng C.A. Meyer	Dauricine	BRAF
Panax ginseng C.A. Meyer	Dauricine	CDK4
Panax ginseng C.A. Meyer	Dauricine	CHRNA4
Panax ginseng C.A. Meyer	Dauricine	CHRNB4
Panax ginseng C.A. Meyer	Dauricine	CSF1R
Panax ginseng C.A. Meyer	Dauricine	DPP4
Panax ginseng C.A. Meyer	Dauricine	DRD1
Panax ginseng C.A. Meyer	Dauricine	DRD2
Panax ginseng C.A. Meyer	Dauricine	DRD3
Panax ginseng C.A. Meyer	Dauricine	ESR1
Panax ginseng C.A. Meyer	Dauricine	ESR2
Panax ginseng C.A. Meyer	Dauricine	F3
Panax ginseng C.A. Meyer	Dauricine	FGFR1
Panax ginseng C.A. Meyer	Dauricine	FGFR3
Panax ginseng C.A. Meyer	Dauricine	HRH3
Panax ginseng C.A. Meyer	Dauricine	HTR1A
Panax ginseng C.A. Meyer	Dauricine	HTR2A
Panax ginseng C.A. Meyer	Dauricine	HTR3A
Panax ginseng C.A. Meyer	Dauricine	IGF1R
Panax ginseng C.A. Meyer	Dauricine	KCNN2

Panax ginseng C.A. Meyer	Dauricine	KCNN3
Panax ginseng C.A. Meyer	Dauricine	KIT
Panax ginseng C.A. Meyer	Dauricine	MAOA
Panax ginseng C.A. Meyer	Dauricine	MAP2
Panax ginseng C.A. Meyer	Dauricine	MAPK10
Panax ginseng C.A. Meyer	Dauricine	MAPK7
Panax ginseng C.A. Meyer	Dauricine	MAPK8
Panax ginseng C.A. Meyer	Dauricine	MTOR
Panax ginseng C.A. Meyer	Dauricine	OPRM1
Panax ginseng C.A. Meyer	Dauricine	PDE1A
Panax ginseng C.A. Meyer	Dauricine	PIK3CA
Panax ginseng C.A. Meyer	Dauricine	PRKCD
Panax ginseng C.A. Meyer	Dauricine	PSEN2
Panax ginseng C.A. Meyer	Dauricine	RAF1
Panax ginseng C.A. Meyer	Dauricine	RET
Panax ginseng C.A. Meyer	Dauricine	SELP
Panax ginseng C.A. Meyer	Dauricine	SIGMAR1
Panax ginseng C.A. Meyer	Dauricine	SLC18A2
Panax ginseng C.A. Meyer	Dauricine	SLC6A2
Panax ginseng C.A. Meyer	Dauricine	SLC6A3
Panax ginseng C.A. Meyer	Dauricine	SLC6A4
Panax ginseng C.A. Meyer	Dauricine	SYK
Panax ginseng C.A. Meyer	Dauricine	TBXA2R
Panax ginseng C.A. Meyer	Dauricine	TEK
Panax ginseng C.A. Meyer	Dauricine	TERT
Panax ginseng C.A. Meyer	Dauricine	TGFBR1
Panax ginseng C.A. Meyer	Deoxyharringtonine	ABCC1
Panax ginseng C.A. Meyer	Deoxyharringtonine	ACHE
Panax ginseng C.A. Meyer	Deoxyharringtonine	ADORA1
Panax ginseng C.A. Meyer	Deoxyharringtonine	ADORA2A
Panax ginseng C.A. Meyer	Deoxyharringtonine	ADRA1A
Panax ginseng C.A. Meyer	Deoxyharringtonine	ADRA2A
Panax ginseng C.A. Meyer	Deoxyharringtonine	ADRA2B
Panax ginseng C.A. Meyer	Deoxyharringtonine	ADRB2
Panax ginseng C.A. Meyer	Deoxyharringtonine	ADRB3
Panax ginseng C.A. Meyer	Deoxyharringtonine	BACE1
Panax ginseng C.A. Meyer	Deoxyharringtonine	BCHE
Panax ginseng C.A. Meyer	Deoxyharringtonine	BIRC2
Panax ginseng C.A. Meyer	Deoxyharringtonine	CASP1
Panax ginseng C.A. Meyer	Deoxyharringtonine	CASP8
Panax ginseng C.A. Meyer	Deoxyharringtonine	CCR3
Panax ginseng C.A. Meyer	Deoxyharringtonine	CDK1
Panax ginseng C.A. Meyer	Deoxyharringtonine	CDK2
Panax ginseng C.A. Meyer	Deoxyharringtonine	CHRM2

Panax ginseng C.A. Meyer	Deoxyharringtonine	CNR2
Panax ginseng C.A. Meyer	Deoxyharringtonine	CTSD
Panax ginseng C.A. Meyer	Deoxyharringtonine	DPP4
Panax ginseng C.A. Meyer	Deoxyharringtonine	DRD1
Panax ginseng C.A. Meyer	Deoxyharringtonine	DRD2
Panax ginseng C.A. Meyer	Deoxyharringtonine	DRD3
Panax ginseng C.A. Meyer	Deoxyharringtonine	EGFR
Panax ginseng C.A. Meyer	Deoxyharringtonine	ELANE
Panax ginseng C.A. Meyer	Deoxyharringtonine	F10
Panax ginseng C.A. Meyer	Deoxyharringtonine	F2
Panax ginseng C.A. Meyer	Deoxyharringtonine	FGFR1
Panax ginseng C.A. Meyer	Deoxyharringtonine	GRIN2B
Panax ginseng C.A. Meyer	Deoxyharringtonine	GSK3B
Panax ginseng C.A. Meyer	Deoxyharringtonine	HRH1
Panax ginseng C.A. Meyer	Deoxyharringtonine	HRH2
Panax ginseng C.A. Meyer	Deoxyharringtonine	HRH3
Panax ginseng C.A. Meyer	Deoxyharringtonine	HSP90AA1
Panax ginseng C.A. Meyer	Deoxyharringtonine	HTR1B
Panax ginseng C.A. Meyer	Deoxyharringtonine	HTR1D
Panax ginseng C.A. Meyer	Deoxyharringtonine	HTR2A
Panax ginseng C.A. Meyer	Deoxyharringtonine	HTR2C
Panax ginseng C.A. Meyer	Deoxyharringtonine	HTR6
Panax ginseng C.A. Meyer	Deoxyharringtonine	IGF1R
Panax ginseng C.A. Meyer	Deoxyharringtonine	JAK2
Panax ginseng C.A. Meyer	Deoxyharringtonine	KDR
Panax ginseng C.A. Meyer	Deoxyharringtonine	KIF11
Panax ginseng C.A. Meyer	Deoxyharringtonine	MAPK14
Panax ginseng C.A. Meyer	Deoxyharringtonine	MDM2
Panax ginseng C.A. Meyer	Deoxyharringtonine	NOS1
Panax ginseng C.A. Meyer	Deoxyharringtonine	NOX4
Panax ginseng C.A. Meyer	Deoxyharringtonine	OPRM1
Panax ginseng C.A. Meyer	Deoxyharringtonine	PARP1
Panax ginseng C.A. Meyer	Deoxyharringtonine	PDE10A
Panax ginseng C.A. Meyer	Deoxyharringtonine	PDGFRA
Panax ginseng C.A. Meyer	Deoxyharringtonine	PIK3CB
Panax ginseng C.A. Meyer	Deoxyharringtonine	PLAU
Panax ginseng C.A. Meyer	Deoxyharringtonine	PRKCA
Panax ginseng C.A. Meyer	Deoxyharringtonine	PRKCB
Panax ginseng C.A. Meyer	Deoxyharringtonine	PRKCD
Panax ginseng C.A. Meyer	Deoxyharringtonine	PRKCE
Panax ginseng C.A. Meyer	Deoxyharringtonine	PRKCG
Panax ginseng C.A. Meyer	Deoxyharringtonine	PSEN2
Panax ginseng C.A. Meyer	Deoxyharringtonine	REN
Panax ginseng C.A. Meyer	Deoxyharringtonine	SCN9A

Panax ginseng C.A. Meyer	Deoxyharringtonine	SLC18A3
Panax ginseng C.A. Meyer	Deoxyharringtonine	SLC6A2
Panax ginseng C.A. Meyer	Deoxyharringtonine	SLC6A4
Panax ginseng C.A. Meyer	Deoxyharringtonine	SRC
Panax ginseng C.A. Meyer	Deoxyharringtonine	SYK
Panax ginseng C.A. Meyer	Deoxyharringtonine	UGCG
Panax ginseng C.A. Meyer	Deoxyharringtonine	XIAP
Panax ginseng C.A. Meyer	d-fenchone	ACHE
Panax ginseng C.A. Meyer	d-fenchone	ADH1C
Panax ginseng C.A. Meyer	d-fenchone	AR
Panax ginseng C.A. Meyer	d-fenchone	CA1
Panax ginseng C.A. Meyer	d-fenchone	CA4
Panax ginseng C.A. Meyer	d-fenchone	G6PD
Panax ginseng C.A. Meyer	d-fenchone	NR1H4
Panax ginseng C.A. Meyer	d-fenchone	VDR
Panax ginseng C.A. Meyer	dibutyl phthalate	ACHE
Panax ginseng C.A. Meyer	dibutyl phthalate	ADAM17
Panax ginseng C.A. Meyer	dibutyl phthalate	ADORA1
Panax ginseng C.A. Meyer	dibutyl phthalate	ADORA2A
Panax ginseng C.A. Meyer	dibutyl phthalate	ADRA2A
Panax ginseng C.A. Meyer	dibutyl phthalate	ADRA2B
Panax ginseng C.A. Meyer	dibutyl phthalate	ADRB3
Panax ginseng C.A. Meyer	dibutyl phthalate	ALDH2
Panax ginseng C.A. Meyer	dibutyl phthalate	CASP3
Panax ginseng C.A. Meyer	dibutyl phthalate	CMA1
Panax ginseng C.A. Meyer	dibutyl phthalate	CRHR1
Panax ginseng C.A. Meyer	dibutyl phthalate	CTSB
Panax ginseng C.A. Meyer	dibutyl phthalate	CTSL
Panax ginseng C.A. Meyer	dibutyl phthalate	CXCR2
Panax ginseng C.A. Meyer	dibutyl phthalate	CYP11B2
Panax ginseng C.A. Meyer	dibutyl phthalate	CYP2C19
Panax ginseng C.A. Meyer	dibutyl phthalate	CYP2C9
Panax ginseng C.A. Meyer	dibutyl phthalate	CYP2D6
Panax ginseng C.A. Meyer	dibutyl phthalate	CYP3A4
Panax ginseng C.A. Meyer	dibutyl phthalate	DRD1
Panax ginseng C.A. Meyer	dibutyl phthalate	DRD3
Panax ginseng C.A. Meyer	dibutyl phthalate	F2
Panax ginseng C.A. Meyer	dibutyl phthalate	FAAH
Panax ginseng C.A. Meyer	dibutyl phthalate	FLT1
Panax ginseng C.A. Meyer	dibutyl phthalate	GALR3
Panax ginseng C.A. Meyer	dibutyl phthalate	GRM2
Panax ginseng C.A. Meyer	dibutyl phthalate	GSK3B
Panax ginseng C.A. Meyer	dibutyl phthalate	HTR2A
Panax ginseng C.A. Meyer	dibutyl phthalate	HTR6

Panax ginseng C.A. Meyer	dibutyl phthalate	MAPK10
Panax ginseng C.A. Meyer	dibutyl phthalate	MAPK14
Panax ginseng C.A. Meyer	dibutyl phthalate	MAPK8
Panax ginseng C.A. Meyer	dibutyl phthalate	MEN1
Panax ginseng C.A. Meyer	dibutyl phthalate	MIF
Panax ginseng C.A. Meyer	dibutyl phthalate	PDE10A
Panax ginseng C.A. Meyer	dibutyl phthalate	PDE2A
Panax ginseng C.A. Meyer	dibutyl phthalate	PGR
Panax ginseng C.A. Meyer	dibutyl phthalate	PLAU
Panax ginseng C.A. Meyer	dibutyl phthalate	PLG
Panax ginseng C.A. Meyer	dibutyl phthalate	PREP
Panax ginseng C.A. Meyer	dibutyl phthalate	PRKCA
Panax ginseng C.A. Meyer	dibutyl phthalate	PTGS1
Panax ginseng C.A. Meyer	dibutyl phthalate	PTGS2
Panax ginseng C.A. Meyer	dibutyl phthalate	RGS4
Panax ginseng C.A. Meyer	dibutyl phthalate	SLC6A2
Panax ginseng C.A. Meyer	dibutyl phthalate	TNFRSF1A
Panax ginseng C.A. Meyer	dibutyl phthalate	TRPC6
Panax ginseng C.A. Meyer	dibutyl phthalate	TSPO
Panax ginseng C.A. Meyer	dibutyl phthalate	VCP
Panax ginseng C.A. Meyer	Dihydroresveratrol	AKR1B1
Panax ginseng C.A. Meyer	Dihydroresveratrol	ALOX12
Panax ginseng C.A. Meyer	Dihydroresveratrol	ALOX5
Panax ginseng C.A. Meyer	Dihydroresveratrol	ALPL
Panax ginseng C.A. Meyer	Dihydroresveratrol	APP
Panax ginseng C.A. Meyer	Dihydroresveratrol	AR
Panax ginseng C.A. Meyer	Dihydroresveratrol	CA4
Panax ginseng C.A. Meyer	Dihydroresveratrol	CDK2
Panax ginseng C.A. Meyer	Dihydroresveratrol	CDK4
Panax ginseng C.A. Meyer	Dihydroresveratrol	CDK5R1
Panax ginseng C.A. Meyer	Dihydroresveratrol	CHRM2
Panax ginseng C.A. Meyer	Dihydroresveratrol	CHRNA7
Panax ginseng C.A. Meyer	Dihydroresveratrol	COMT
Panax ginseng C.A. Meyer	Dihydroresveratrol	CYP2C19
Panax ginseng C.A. Meyer	Dihydroresveratrol	CYP3A4
Panax ginseng C.A. Meyer	Dihydroresveratrol	DAO
Panax ginseng C.A. Meyer	Dihydroresveratrol	DRD2
Panax ginseng C.A. Meyer	Dihydroresveratrol	ERN1
Panax ginseng C.A. Meyer	Dihydroresveratrol	ESR1
Panax ginseng C.A. Meyer	Dihydroresveratrol	ESR2
Panax ginseng C.A. Meyer	Dihydroresveratrol	F3
Panax ginseng C.A. Meyer	Dihydroresveratrol	FGFR1
Panax ginseng C.A. Meyer	Dihydroresveratrol	FYN
Panax ginseng C.A. Meyer	Dihydroresveratrol	GSK3B

Panax ginseng C.A. Meyer	Dihydroresveratrol	HSP90AA1
Panax ginseng C.A. Meyer	Dihydroresveratrol	IGF1R
Panax ginseng C.A. Meyer	Dihydroresveratrol	INSR
Panax ginseng C.A. Meyer	Dihydroresveratrol	KIT
Panax ginseng C.A. Meyer	Dihydroresveratrol	MAP2K1
Panax ginseng C.A. Meyer	Dihydroresveratrol	MAPK14
Panax ginseng C.A. Meyer	Dihydroresveratrol	MMP1
Panax ginseng C.A. Meyer	Dihydroresveratrol	MMP2
Panax ginseng C.A. Meyer	Dihydroresveratrol	MMP9
Panax ginseng C.A. Meyer	Dihydroresveratrol	NR3C1
Panax ginseng C.A. Meyer	Dihydroresveratrol	PARP1
Panax ginseng C.A. Meyer	Dihydroresveratrol	PIK3CA
Panax ginseng C.A. Meyer	Dihydroresveratrol	PIK3CB
Panax ginseng C.A. Meyer	Dihydroresveratrol	PIK3CG
Panax ginseng C.A. Meyer	Dihydroresveratrol	PTGS1
Panax ginseng C.A. Meyer	Dihydroresveratrol	PTGS2
Panax ginseng C.A. Meyer	Dihydroresveratrol	SLC6A2
Panax ginseng C.A. Meyer	Dihydroresveratrol	TGFBR1
Panax ginseng C.A. Meyer	Dihydroresveratrol	TTR
Panax ginseng C.A. Meyer	Dihydroresveratrol	TYR
Panax ginseng C.A. Meyer	Diop	EGFR
Panax ginseng C.A. Meyer	Diop	SRC
Panax ginseng C.A. Meyer	Ditertbutyl phthalate	ABCB1
Panax ginseng C.A. Meyer	Ditertbutyl phthalate	ACHE
Panax ginseng C.A. Meyer	Ditertbutyl phthalate	ADORA1
Panax ginseng C.A. Meyer	Ditertbutyl phthalate	ADORA2A
Panax ginseng C.A. Meyer	Ditertbutyl phthalate	ADRB3
Panax ginseng C.A. Meyer	Ditertbutyl phthalate	BCHE
Panax ginseng C.A. Meyer	Ditertbutyl phthalate	CACNA1C
Panax ginseng C.A. Meyer	Ditertbutyl phthalate	CNR2
Panax ginseng C.A. Meyer	Ditertbutyl phthalate	CTSB
Panax ginseng C.A. Meyer	Ditertbutyl phthalate	CTSL
Panax ginseng C.A. Meyer	Ditertbutyl phthalate	CXCR2
Panax ginseng C.A. Meyer	Ditertbutyl phthalate	CYP11B2
Panax ginseng C.A. Meyer	Ditertbutyl phthalate	CYP2C19
Panax ginseng C.A. Meyer	Ditertbutyl phthalate	CYP2C9
Panax ginseng C.A. Meyer	Ditertbutyl phthalate	CYP3A4
Panax ginseng C.A. Meyer	Ditertbutyl phthalate	DRD1
Panax ginseng C.A. Meyer	Ditertbutyl phthalate	DRD3
Panax ginseng C.A. Meyer	Ditertbutyl phthalate	F2
Panax ginseng C.A. Meyer	Ditertbutyl phthalate	GALR3
Panax ginseng C.A. Meyer	Ditertbutyl phthalate	MEN1
Panax ginseng C.A. Meyer	Ditertbutyl phthalate	MIF
Panax ginseng C.A. Meyer	Ditertbutyl phthalate	PDE10A

Panax ginseng C.A. Meyer	Ditertbutyl phthalate	PDE2A
Panax ginseng C.A. Meyer	Ditertbutyl phthalate	PDE3A
Panax ginseng C.A. Meyer	Ditertbutyl phthalate	PDE4A
Panax ginseng C.A. Meyer	Ditertbutyl phthalate	PDE5A
Panax ginseng C.A. Meyer	Ditertbutyl phthalate	PIK3CD
Panax ginseng C.A. Meyer	Ditertbutyl phthalate	PLA2G2A
Panax ginseng C.A. Meyer	Ditertbutyl phthalate	PLAU
Panax ginseng C.A. Meyer	Ditertbutyl phthalate	PREP
Panax ginseng C.A. Meyer	Ditertbutyl phthalate	PTGS2
Panax ginseng C.A. Meyer	Ditertbutyl phthalate	RGS4
Panax ginseng C.A. Meyer	Ditertbutyl phthalate	TSPO
Panax ginseng C.A. Meyer	DL-Pantothenic acid	AKR1B1
Panax ginseng C.A. Meyer	DL-Pantothenic acid	CA1
Panax ginseng C.A. Meyer	DL-Pantothenic acid	ECE1
Panax ginseng C.A. Meyer	DL-Pantothenic acid	FOLH1
Panax ginseng C.A. Meyer	DL-Pantothenic acid	SLC13A5
Panax ginseng C.A. Meyer	eicosane	AR
Panax ginseng C.A. Meyer	eicosane	CA1
Panax ginseng C.A. Meyer	eicosane	CA4
Panax ginseng C.A. Meyer	eicosane	CNR1
Panax ginseng C.A. Meyer	eicosane	CNR2
Panax ginseng C.A. Meyer	eicosane	CYP11B2
Panax ginseng C.A. Meyer	eicosane	CYP3A4
Panax ginseng C.A. Meyer	eicosane	ESR1
Panax ginseng C.A. Meyer	eicosane	ESR2
Panax ginseng C.A. Meyer	eicosane	HMGCR
Panax ginseng C.A. Meyer	eicosane	HSD11B1
Panax ginseng C.A. Meyer	eicosane	JAK2
Panax ginseng C.A. Meyer	eicosane	NR1H2
Panax ginseng C.A. Meyer	eicosane	NR1H4
Panax ginseng C.A. Meyer	eicosane	NR3C1
Panax ginseng C.A. Meyer	eicosane	PGR
Panax ginseng C.A. Meyer	eicosane	PRKCA
Panax ginseng C.A. Meyer	eicosane	PRKCD
Panax ginseng C.A. Meyer	eicosane	PSEN2
Panax ginseng C.A. Meyer	eicosane	PTGS2
Panax ginseng C.A. Meyer	eicosane	RBP4
Panax ginseng C.A. Meyer	eicosane	SHH
Panax ginseng C.A. Meyer	Elemicin	ACHE
Panax ginseng C.A. Meyer	Elemicin	ADORA1
Panax ginseng C.A. Meyer	Elemicin	ADORA2A
Panax ginseng C.A. Meyer	Elemicin	BCHE
Panax ginseng C.A. Meyer	Elemicin	BDKRB2
Panax ginseng C.A. Meyer	Elemicin	CASP3

Panax ginseng C.A. Meyer	Elemicin	CDK1
Panax ginseng C.A. Meyer	Elemicin	CDK2
Panax ginseng C.A. Meyer	Elemicin	CHRM1
Panax ginseng C.A. Meyer	Elemicin	CHRM2
Panax ginseng C.A. Meyer	Elemicin	CREBBP
Panax ginseng C.A. Meyer	Elemicin	CTSF
Panax ginseng C.A. Meyer	Elemicin	CYP11B2
Panax ginseng C.A. Meyer	Elemicin	EP300
Panax ginseng C.A. Meyer	Elemicin	GALR3
Panax ginseng C.A. Meyer	Elemicin	GRM2
Panax ginseng C.A. Meyer	Elemicin	HDAC1
Panax ginseng C.A. Meyer	Elemicin	HMGCR
Panax ginseng C.A. Meyer	Elemicin	HMOX1
Panax ginseng C.A. Meyer	Elemicin	HSD11B1
Panax ginseng C.A. Meyer	Elemicin	HTR2A
Panax ginseng C.A. Meyer	Elemicin	IDO1
Panax ginseng C.A. Meyer	Elemicin	JAK2
Panax ginseng C.A. Meyer	Elemicin	KDR
Panax ginseng C.A. Meyer	Elemicin	MAPK10
Panax ginseng C.A. Meyer	Elemicin	MIF
Panax ginseng C.A. Meyer	Elemicin	NOS1
Panax ginseng C.A. Meyer	Elemicin	NOS3
Panax ginseng C.A. Meyer	Elemicin	NQO1
Panax ginseng C.A. Meyer	Elemicin	P2RX7
Panax ginseng C.A. Meyer	Elemicin	PDE10A
Panax ginseng C.A. Meyer	Elemicin	PIK3CA
Panax ginseng C.A. Meyer	Elemicin	PIK3CB
Panax ginseng C.A. Meyer	Elemicin	PTGS1
Panax ginseng C.A. Meyer	Elemicin	QPCT
Panax ginseng C.A. Meyer	Elemicin	RGS4
Panax ginseng C.A. Meyer	Elemicin	SRC
Panax ginseng C.A. Meyer	Elemicin	STAT3
Panax ginseng C.A. Meyer	Elemicin	TGM2
Panax ginseng C.A. Meyer	Enhydrin	ABCB1
Panax ginseng C.A. Meyer	Enhydrin	ABCC9
Panax ginseng C.A. Meyer	Enhydrin	ADAM17
Panax ginseng C.A. Meyer	Enhydrin	ADORA1
Panax ginseng C.A. Meyer	Enhydrin	ADORA2A
Panax ginseng C.A. Meyer	Enhydrin	ALPL
Panax ginseng C.A. Meyer	Enhydrin	AR
Panax ginseng C.A. Meyer	Enhydrin	CAPN1
Panax ginseng C.A. Meyer	Enhydrin	CAPN2
Panax ginseng C.A. Meyer	Enhydrin	CASP1
Panax ginseng C.A. Meyer	Enhydrin	CASP3

Panax ginseng C.A. Meyer	Enhydrin	CASP8
Panax ginseng C.A. Meyer	Enhydrin	CCNA2
Panax ginseng C.A. Meyer	Enhydrin	CCND1
Panax ginseng C.A. Meyer	Enhydrin	CDK2
Panax ginseng C.A. Meyer	Enhydrin	CDK5R1
Panax ginseng C.A. Meyer	Enhydrin	CXCR2
Panax ginseng C.A. Meyer	Enhydrin	DYRK1A
Panax ginseng C.A. Meyer	Enhydrin	ELANE
Panax ginseng C.A. Meyer	Enhydrin	F2
Panax ginseng C.A. Meyer	Enhydrin	F2RL1
Panax ginseng C.A. Meyer	Enhydrin	GLI1
Panax ginseng C.A. Meyer	Enhydrin	GRK2
Panax ginseng C.A. Meyer	Enhydrin	HSD11B1
Panax ginseng C.A. Meyer	Enhydrin	HTR2A
Panax ginseng C.A. Meyer	Enhydrin	IDO1
Panax ginseng C.A. Meyer	Enhydrin	JAK2
Panax ginseng C.A. Meyer	Enhydrin	JUN
Panax ginseng C.A. Meyer	Enhydrin	LRRK2
Panax ginseng C.A. Meyer	Enhydrin	MAPK1
Panax ginseng C.A. Meyer	Enhydrin	MAPK14
Panax ginseng C.A. Meyer	Enhydrin	MAPK8
Panax ginseng C.A. Meyer	Enhydrin	NOS2
Panax ginseng C.A. Meyer	Enhydrin	PDE10A
Panax ginseng C.A. Meyer	Enhydrin	PIK3CA
Panax ginseng C.A. Meyer	Enhydrin	PRKCA
Panax ginseng C.A. Meyer	Enhydrin	PRKCB
Panax ginseng C.A. Meyer	Enhydrin	PRKCD
Panax ginseng C.A. Meyer	Enhydrin	PRKCE
Panax ginseng C.A. Meyer	Enhydrin	PRKCG
Panax ginseng C.A. Meyer	Enhydrin	PRKCH
Panax ginseng C.A. Meyer	Enhydrin	PTGS1
Panax ginseng C.A. Meyer	Enhydrin	PTGS2
Panax ginseng C.A. Meyer	Enhydrin	PTPN1
Panax ginseng C.A. Meyer	Enhydrin	SCN9A
Panax ginseng C.A. Meyer	Enhydrin	SRC
Panax ginseng C.A. Meyer	Enhydrin	TERT
Panax ginseng C.A. Meyer	Enhydrin	TRPV4
Panax ginseng C.A. Meyer	Fumarine	ADRA1A
Panax ginseng C.A. Meyer	Fumarine	APP
Panax ginseng C.A. Meyer	Fumarine	CDK2
Panax ginseng C.A. Meyer	Fumarine	DRD1
Panax ginseng C.A. Meyer	Fumarine	DRD2
Panax ginseng C.A. Meyer	Fumarine	DRD3
Panax ginseng C.A. Meyer	Fumarine	ERN1

Panax ginseng C.A. Meyer	Fumarine	F3
Panax ginseng C.A. Meyer	Fumarine	HTR1A
Panax ginseng C.A. Meyer	Fumarine	JAK2
Panax ginseng C.A. Meyer	Fumarine	LRRK2
Panax ginseng C.A. Meyer	Fumarine	MAPK8
Panax ginseng C.A. Meyer	Fumarine	NEK1
Panax ginseng C.A. Meyer	Fumarine	PIK3CA
Panax ginseng C.A. Meyer	Fumarine	PIK3CB
Panax ginseng C.A. Meyer	Fumarine	PIK3CD
Panax ginseng C.A. Meyer	Fumarine	PTGS2
Panax ginseng C.A. Meyer	Fumarine	SIGMAR1
Panax ginseng C.A. Meyer	Fumarine	SLC6A3
Panax ginseng C.A. Meyer	Fumarine	TYMS
Panax ginseng C.A. Meyer	Ginsenol	ALOX5
Panax ginseng C.A. Meyer	Ginsenol	AR
Panax ginseng C.A. Meyer	Ginsenol	C5AR1
Panax ginseng C.A. Meyer	Ginsenol	CA1
Panax ginseng C.A. Meyer	Ginsenol	CA4
Panax ginseng C.A. Meyer	Ginsenol	CHRM1
Panax ginseng C.A. Meyer	Ginsenol	CNR1
Panax ginseng C.A. Meyer	Ginsenol	CNR2
Panax ginseng C.A. Meyer	Ginsenol	CYP11B2
Panax ginseng C.A. Meyer	Ginsenol	DRD2
Panax ginseng C.A. Meyer	Ginsenol	ESR1
Panax ginseng C.A. Meyer	Ginsenol	ESR2
Panax ginseng C.A. Meyer	Ginsenol	G6PD
Panax ginseng C.A. Meyer	Ginsenol	HIF1A
Panax ginseng C.A. Meyer	Ginsenol	HRH3
Panax ginseng C.A. Meyer	Ginsenol	HSD11B1
Panax ginseng C.A. Meyer	Ginsenol	HTR2A
Panax ginseng C.A. Meyer	Ginsenol	IDO1
Panax ginseng C.A. Meyer	Ginsenol	IGF1R
Panax ginseng C.A. Meyer	Ginsenol	KIF11
Panax ginseng C.A. Meyer	Ginsenol	MAPK8
Panax ginseng C.A. Meyer	Ginsenol	MDM2
Panax ginseng C.A. Meyer	Ginsenol	NR1H4
Panax ginseng C.A. Meyer	Ginsenol	NR3C1
Panax ginseng C.A. Meyer	Ginsenol	NR3C2
Panax ginseng C.A. Meyer	Ginsenol	NR4A1
Panax ginseng C.A. Meyer	Ginsenol	OXTR
Panax ginseng C.A. Meyer	Ginsenol	P2RX7
Panax ginseng C.A. Meyer	Ginsenol	PARP1
Panax ginseng C.A. Meyer	Ginsenol	PGR
Panax ginseng C.A. Meyer	Ginsenol	PIK3CA

Panax ginseng C.A. Meyer	Ginsenol	PIK3CB
Panax ginseng C.A. Meyer	Ginsenol	PSEN2
Panax ginseng C.A. Meyer	Ginsenol	PTGS2
Panax ginseng C.A. Meyer	Ginsenol	PTK2B
Panax ginseng C.A. Meyer	Ginsenol	SHH
Panax ginseng C.A. Meyer	Ginsenol	SIGMAR1
Panax ginseng C.A. Meyer	Ginsenol	SLC18A3
Panax ginseng C.A. Meyer	Ginsenol	SLC6A3
Panax ginseng C.A. Meyer	Ginsenol	TACR1
Panax ginseng C.A. Meyer	Ginsenol	VDR
Panax ginseng C.A. Meyer	Girinimbin	ABCC1
Panax ginseng C.A. Meyer	Girinimbin	ADORA1
Panax ginseng C.A. Meyer	Girinimbin	ADORA2A
Panax ginseng C.A. Meyer	Girinimbin	ADRA2A
Panax ginseng C.A. Meyer	Girinimbin	ADRA2B
Panax ginseng C.A. Meyer	Girinimbin	ADRB3
Panax ginseng C.A. Meyer	Girinimbin	AKT1
Panax ginseng C.A. Meyer	Girinimbin	ALK
Panax ginseng C.A. Meyer	Girinimbin	APP
Panax ginseng C.A. Meyer	Girinimbin	AR
Panax ginseng C.A. Meyer	Girinimbin	BCHE
Panax ginseng C.A. Meyer	Girinimbin	CDK1
Panax ginseng C.A. Meyer	Girinimbin	CNR1
Panax ginseng C.A. Meyer	Girinimbin	CRHR1
Panax ginseng C.A. Meyer	Girinimbin	CTSB
Panax ginseng C.A. Meyer	Girinimbin	DYRK1A
Panax ginseng C.A. Meyer	Girinimbin	EGFR
Panax ginseng C.A. Meyer	Girinimbin	ELANE
Panax ginseng C.A. Meyer	Girinimbin	FAAH
Panax ginseng C.A. Meyer	Girinimbin	FGFR1
Panax ginseng C.A. Meyer	Girinimbin	FLT1
Panax ginseng C.A. Meyer	Girinimbin	GRIN1
Panax ginseng C.A. Meyer	Girinimbin	GRM2
Panax ginseng C.A. Meyer	Girinimbin	HRH1
Panax ginseng C.A. Meyer	Girinimbin	HRH2
Panax ginseng C.A. Meyer	Girinimbin	HRH3
Panax ginseng C.A. Meyer	Girinimbin	HSD11B1
Panax ginseng C.A. Meyer	Girinimbin	HTR1A
Panax ginseng C.A. Meyer	Girinimbin	HTR2A
Panax ginseng C.A. Meyer	Girinimbin	HTR2C
Panax ginseng C.A. Meyer	Girinimbin	HTR6
Panax ginseng C.A. Meyer	Girinimbin	IDH1
Panax ginseng C.A. Meyer	Girinimbin	KDR
Panax ginseng C.A. Meyer	Girinimbin	MAPK14

Panax ginseng C.A. Meyer	Girinimbin	MDM2
Panax ginseng C.A. Meyer	Girinimbin	NR1H2
Panax ginseng C.A. Meyer	Girinimbin	NR1H4
Panax ginseng C.A. Meyer	Girinimbin	NR3C1
Panax ginseng C.A. Meyer	Girinimbin	OXTR
Panax ginseng C.A. Meyer	Girinimbin	P2RX7
Panax ginseng C.A. Meyer	Girinimbin	PDE10A
Panax ginseng C.A. Meyer	Girinimbin	PDE5A
Panax ginseng C.A. Meyer	Girinimbin	PDGFRB
Panax ginseng C.A. Meyer	Girinimbin	PSEN2
Panax ginseng C.A. Meyer	Girinimbin	QPCT
Panax ginseng C.A. Meyer	Girinimbin	RET
Panax ginseng C.A. Meyer	Girinimbin	SLC6A3
Panax ginseng C.A. Meyer	Girinimbin	SLC6A4
Panax ginseng C.A. Meyer	Girinimbin	TACR1
Panax ginseng C.A. Meyer	Girinimbin	TSPO
Panax ginseng C.A. Meyer	Girinimbin	TYMS
Panax ginseng C.A. Meyer	Girinimbin	VCP
Panax ginseng C.A. Meyer	Hexadecanoic Acid	ABCB1
Panax ginseng C.A. Meyer	Hexadecanoic Acid	ABCC1
Panax ginseng C.A. Meyer	Hexadecanoic Acid	ALOX12
Panax ginseng C.A. Meyer	Hexadecanoic Acid	AR
Panax ginseng C.A. Meyer	Hexadecanoic Acid	CA1
Panax ginseng C.A. Meyer	Hexadecanoic Acid	CHRNA7
Panax ginseng C.A. Meyer	Hexadecanoic Acid	FAAH
Panax ginseng C.A. Meyer	Hexadecanoic Acid	FABP3
Panax ginseng C.A. Meyer	Hexadecanoic Acid	G6PD
Panax ginseng C.A. Meyer	Hexadecanoic Acid	HSD11B1
Panax ginseng C.A. Meyer	Hexadecanoic Acid	ITGAL
Panax ginseng C.A. Meyer	Hexadecanoic Acid	MAPK1
Panax ginseng C.A. Meyer	Hexadecanoic Acid	NR1H4
Panax ginseng C.A. Meyer	Hexadecanoic Acid	PLA2G4A
Panax ginseng C.A. Meyer	Hexadecanoic Acid	PLG
Panax ginseng C.A. Meyer	Hexadecanoic Acid	PPARA
Panax ginseng C.A. Meyer	Hexadecanoic Acid	PPARD
Panax ginseng C.A. Meyer	Hexadecanoic Acid	PPARG
Panax ginseng C.A. Meyer	Hexadecanoic Acid	PTPN1
Panax ginseng C.A. Meyer	Hexadecanoic Acid	PTPRC
Panax ginseng C.A. Meyer	Hexadecanoic Acid	RARA
Panax ginseng C.A. Meyer	Hexadecanoic Acid	RXRA
Panax ginseng C.A. Meyer	Hexadecanoic Acid	TERT
Panax ginseng C.A. Meyer	Hexadecanoic Acid	VDR
Panax ginseng C.A. Meyer	Malvic Acid	ACHE
Panax ginseng C.A. Meyer	Malvic Acid	ACP1

Panax ginseng C.A. Meyer	Malvic Acid	AKR1B1
Panax ginseng C.A. Meyer	Malvic Acid	ALOX5
Panax ginseng C.A. Meyer	Malvic Acid	AR
Panax ginseng C.A. Meyer	Malvic Acid	BCHE
Panax ginseng C.A. Meyer	Malvic Acid	CNR1
Panax ginseng C.A. Meyer	Malvic Acid	CYP2C19
Panax ginseng C.A. Meyer	Malvic Acid	CYP3A4
Panax ginseng C.A. Meyer	Malvic Acid	ESR1
Panax ginseng C.A. Meyer	Malvic Acid	ESR2
Panax ginseng C.A. Meyer	Malvic Acid	FAAH
Panax ginseng C.A. Meyer	Malvic Acid	FABP3
Panax ginseng C.A. Meyer	Malvic Acid	G6PD
Panax ginseng C.A. Meyer	Malvic Acid	HMGCR
Panax ginseng C.A. Meyer	Malvic Acid	HSD11B1
Panax ginseng C.A. Meyer	Malvic Acid	MAPK3
Panax ginseng C.A. Meyer	Malvic Acid	MDM2
Panax ginseng C.A. Meyer	Malvic Acid	MME
Panax ginseng C.A. Meyer	Malvic Acid	NOS2
Panax ginseng C.A. Meyer	Malvic Acid	NR1H4
Panax ginseng C.A. Meyer	Malvic Acid	NR3C1
Panax ginseng C.A. Meyer	Malvic Acid	NR3C2
Panax ginseng C.A. Meyer	Malvic Acid	PDE4A
Panax ginseng C.A. Meyer	Malvic Acid	PGR
Panax ginseng C.A. Meyer	Malvic Acid	PLA2G1B
Panax ginseng C.A. Meyer	Malvic Acid	PPARA
Panax ginseng C.A. Meyer	Malvic Acid	PPARD
Panax ginseng C.A. Meyer	Malvic Acid	PPARG
Panax ginseng C.A. Meyer	Malvic Acid	PREP
Panax ginseng C.A. Meyer	Malvic Acid	PRKCH
Panax ginseng C.A. Meyer	Malvic Acid	PSEN1
Panax ginseng C.A. Meyer	Malvic Acid	PSEN2
Panax ginseng C.A. Meyer	Malvic Acid	PTGIR
Panax ginseng C.A. Meyer	Malvic Acid	PTGS1
Panax ginseng C.A. Meyer	Malvic Acid	PTGS2
Panax ginseng C.A. Meyer	Malvic Acid	PTPN1
Panax ginseng C.A. Meyer	Malvic Acid	PTPN11
Panax ginseng C.A. Meyer	Malvic Acid	PTPN6
Panax ginseng C.A. Meyer	Malvic Acid	SIGMAR1
Panax ginseng C.A. Meyer	Malvic Acid	SLC6A2
Panax ginseng C.A. Meyer	Malvic Acid	SLC6A4
Panax ginseng C.A. Meyer	Malvic Acid	TERT
Panax ginseng C.A. Meyer	Malvic Acid	TOP1
Panax ginseng C.A. Meyer	Menthyl Acetate	ACHE
Panax ginseng C.A. Meyer	Menthyl Acetate	ADRA1A

Panax ginseng C.A. Meyer	Menthyl Acetate	ADRA2A
Panax ginseng C.A. Meyer	Menthyl Acetate	ADRA2B
Panax ginseng C.A. Meyer	Menthyl Acetate	CA1
Panax ginseng C.A. Meyer	Menthyl Acetate	CHRNA4
Panax ginseng C.A. Meyer	Menthyl Acetate	CTSB
Panax ginseng C.A. Meyer	Menthyl Acetate	CTSL
Panax ginseng C.A. Meyer	Menthyl Acetate	CYP11B2
Panax ginseng C.A. Meyer	Menthyl Acetate	ELANE
Panax ginseng C.A. Meyer	Menthyl Acetate	GSK3B
Panax ginseng C.A. Meyer	Menthyl Acetate	HMGCR
Panax ginseng C.A. Meyer	Menthyl Acetate	HSD11B1
Panax ginseng C.A. Meyer	Menthyl Acetate	HTR1A
Panax ginseng C.A. Meyer	Menthyl Acetate	MPO
Panax ginseng C.A. Meyer	Menthyl Acetate	P2RX7
Panax ginseng C.A. Meyer	Menthyl Acetate	PDE10A
Panax ginseng C.A. Meyer	Menthyl Acetate	PLA2G6
Panax ginseng C.A. Meyer	Menthyl Acetate	PTGS1
Panax ginseng C.A. Meyer	Menthyl Acetate	PTPN1
Panax ginseng C.A. Meyer	Menthyl Acetate	QPCT
Panax ginseng C.A. Meyer	Menthyl Acetate	SLC6A2
Panax ginseng C.A. Meyer	Menthyl Acetate	VDR
Panax ginseng C.A. Meyer	Methyl Palmitate	ALOX5
Panax ginseng C.A. Meyer	Methyl Palmitate	CA1
Panax ginseng C.A. Meyer	Methyl Palmitate	F2R
Panax ginseng C.A. Meyer	Methyl Palmitate	FABP3
Panax ginseng C.A. Meyer	Methyl Palmitate	G6PD
Panax ginseng C.A. Meyer	Methyl Palmitate	HMGCR
Panax ginseng C.A. Meyer	Methyl Palmitate	HSD11B1
Panax ginseng C.A. Meyer	Methyl Palmitate	NR1H4
Panax ginseng C.A. Meyer	Methyl Palmitate	PPARA
Panax ginseng C.A. Meyer	Methyl Palmitate	PPARD
Panax ginseng C.A. Meyer	Methyl Palmitate	PTPN1
Panax ginseng C.A. Meyer	Methyl Palmitate	SLC1A1
Panax ginseng C.A. Meyer	Methyl Palmitate	VDR
Panax ginseng C.A. Meyer	Octanal	ADH1C
Panax ginseng C.A. Meyer	Octanal	AR
Panax ginseng C.A. Meyer	Octanal	CA1
Panax ginseng C.A. Meyer	Octanal	CA4
Panax ginseng C.A. Meyer	Octanal	CHRM1
Panax ginseng C.A. Meyer	Octanal	CHRM2
Panax ginseng C.A. Meyer	Octanal	HSPA1A
Panax ginseng C.A. Meyer	Octanal	MAOA
Panax ginseng C.A. Meyer	Octanal	MAOB
Panax ginseng C.A. Meyer	Octanal	NR4A1

Panax ginseng C.A. Meyer	Octanal	PGR
Panax ginseng C.A. Meyer	Octanal	PTK2B
Panax ginseng C.A. Meyer	Palmitoleic Acid	ACHE
Panax ginseng C.A. Meyer	Palmitoleic Acid	ACP1
Panax ginseng C.A. Meyer	Palmitoleic Acid	ALOX12
Panax ginseng C.A. Meyer	Palmitoleic Acid	ALOX5
Panax ginseng C.A. Meyer	Palmitoleic Acid	AR
Panax ginseng C.A. Meyer	Palmitoleic Acid	BACE1
Panax ginseng C.A. Meyer	Palmitoleic Acid	BCHE
Panax ginseng C.A. Meyer	Palmitoleic Acid	BCL2
Panax ginseng C.A. Meyer	Palmitoleic Acid	CHRM2
Panax ginseng C.A. Meyer	Palmitoleic Acid	CMA1
Panax ginseng C.A. Meyer	Palmitoleic Acid	CNR1
Panax ginseng C.A. Meyer	Palmitoleic Acid	CYP2C19
Panax ginseng C.A. Meyer	Palmitoleic Acid	EDNRA
Panax ginseng C.A. Meyer	Palmitoleic Acid	ESR1
Panax ginseng C.A. Meyer	Palmitoleic Acid	ESR2
Panax ginseng C.A. Meyer	Palmitoleic Acid	FAAH
Panax ginseng C.A. Meyer	Palmitoleic Acid	FABP3
Panax ginseng C.A. Meyer	Palmitoleic Acid	G6PD
Panax ginseng C.A. Meyer	Palmitoleic Acid	HMGCR
Panax ginseng C.A. Meyer	Palmitoleic Acid	HSD11B1
Panax ginseng C.A. Meyer	Palmitoleic Acid	MAPK3
Panax ginseng C.A. Meyer	Palmitoleic Acid	MDM2
Panax ginseng C.A. Meyer	Palmitoleic Acid	NOS2
Panax ginseng C.A. Meyer	Palmitoleic Acid	NR1H4
Panax ginseng C.A. Meyer	Palmitoleic Acid	NR3C1
Panax ginseng C.A. Meyer	Palmitoleic Acid	NR3C2
Panax ginseng C.A. Meyer	Palmitoleic Acid	PGR
Panax ginseng C.A. Meyer	Palmitoleic Acid	PLA2G1B
Panax ginseng C.A. Meyer	Palmitoleic Acid	PPARA
Panax ginseng C.A. Meyer	Palmitoleic Acid	PPARD
Panax ginseng C.A. Meyer	Palmitoleic Acid	PPARG
Panax ginseng C.A. Meyer	Palmitoleic Acid	PREP
Panax ginseng C.A. Meyer	Palmitoleic Acid	PRKCH
Panax ginseng C.A. Meyer	Palmitoleic Acid	PSEN2
Panax ginseng C.A. Meyer	Palmitoleic Acid	PTGIR
Panax ginseng C.A. Meyer	Palmitoleic Acid	PTGS1
Panax ginseng C.A. Meyer	Palmitoleic Acid	PTGS2
Panax ginseng C.A. Meyer	Palmitoleic Acid	PTPN1
Panax ginseng C.A. Meyer	Palmitoleic Acid	PTPN11
Panax ginseng C.A. Meyer	Palmitoleic Acid	PTPN6
Panax ginseng C.A. Meyer	Palmitoleic Acid	SIGMAR1
Panax ginseng C.A. Meyer	Palmitoleic Acid	SLC6A2

Panax ginseng C.A. Meyer	Palmitoleic Acid	SLC6A3
Panax ginseng C.A. Meyer	Palmitoleic Acid	SLC6A4
Panax ginseng C.A. Meyer	Palmitoleic Acid	TERT
Panax ginseng C.A. Meyer	Palmitoleic Acid	TOP1
Panax ginseng C.A. Meyer	Panasinsanol A	AR
Panax ginseng C.A. Meyer	Panasinsanol A	CA1
Panax ginseng C.A. Meyer	Panasinsanol A	CA4
Panax ginseng C.A. Meyer	Panasinsanol A	ESR1
Panax ginseng C.A. Meyer	Panasinsanol A	ESR2
Panax ginseng C.A. Meyer	Panasinsanol A	HSD11B1
Panax ginseng C.A. Meyer	Panasinsanol A	NR1H4
Panax ginseng C.A. Meyer	Panasinsanol A	SHH
Panax ginseng C.A. Meyer	Panasinsanol B	AR
Panax ginseng C.A. Meyer	Panasinsanol B	CA1
Panax ginseng C.A. Meyer	Panasinsanol B	CA4
Panax ginseng C.A. Meyer	Panasinsanol B	ESR1
Panax ginseng C.A. Meyer	Panasinsanol B	ESR2
Panax ginseng C.A. Meyer	Panasinsanol B	HSD11B1
Panax ginseng C.A. Meyer	Panasinsanol B	NR1H4
Panax ginseng C.A. Meyer	Panasinsanol B	SHH
Panax ginseng C.A. Meyer	Panaxacol	ABCB1
Panax ginseng C.A. Meyer	Panaxacol	ABL1
Panax ginseng C.A. Meyer	Panaxacol	ADA
Panax ginseng C.A. Meyer	Panaxacol	ADAM10
Panax ginseng C.A. Meyer	Panaxacol	ADAM17
Panax ginseng C.A. Meyer	Panaxacol	ADORA1
Panax ginseng C.A. Meyer	Panaxacol	ADORA2A
Panax ginseng C.A. Meyer	Panaxacol	ALOX5
Panax ginseng C.A. Meyer	Panaxacol	AR
Panax ginseng C.A. Meyer	Panaxacol	CA1
Panax ginseng C.A. Meyer	Panaxacol	CASP1
Panax ginseng C.A. Meyer	Panaxacol	CASP8
Panax ginseng C.A. Meyer	Panaxacol	CCNA2
Panax ginseng C.A. Meyer	Panaxacol	CCR1
Panax ginseng C.A. Meyer	Panaxacol	CCR3
Panax ginseng C.A. Meyer	Panaxacol	CHRM1
Panax ginseng C.A. Meyer	Panaxacol	CNR2
Panax ginseng C.A. Meyer	Panaxacol	CSF1R
Panax ginseng C.A. Meyer	Panaxacol	CTSB
Panax ginseng C.A. Meyer	Panaxacol	CTSL
Panax ginseng C.A. Meyer	Panaxacol	DPP4
Panax ginseng C.A. Meyer	Panaxacol	DRD2
Panax ginseng C.A. Meyer	Panaxacol	ECE1
Panax ginseng C.A. Meyer	Panaxacol	EDNRA

Panax ginseng C.A. Meyer	Panaxacol	EGFR
Panax ginseng C.A. Meyer	Panaxacol	F10
Panax ginseng C.A. Meyer	Panaxacol	F2R
Panax ginseng C.A. Meyer	Panaxacol	F9
Panax ginseng C.A. Meyer	Panaxacol	FAAH
Panax ginseng C.A. Meyer	Panaxacol	HMGCR
Panax ginseng C.A. Meyer	Panaxacol	HRH1
Panax ginseng C.A. Meyer	Panaxacol	HTR1A
Panax ginseng C.A. Meyer	Panaxacol	JAK2
Panax ginseng C.A. Meyer	Panaxacol	KDR
Panax ginseng C.A. Meyer	Panaxacol	MAP3K14
Panax ginseng C.A. Meyer	Panaxacol	MAPK1
Panax ginseng C.A. Meyer	Panaxacol	MAPK14
Panax ginseng C.A. Meyer	Panaxacol	MAPK3
Panax ginseng C.A. Meyer	Panaxacol	MAPK8
Panax ginseng C.A. Meyer	Panaxacol	MDM2
Panax ginseng C.A. Meyer	Panaxacol	MME
Panax ginseng C.A. Meyer	Panaxacol	MTOR
Panax ginseng C.A. Meyer	Panaxacol	PDE10A
Panax ginseng C.A. Meyer	Panaxacol	PDE2A
Panax ginseng C.A. Meyer	Panaxacol	PDE5A
Panax ginseng C.A. Meyer	Panaxacol	PIK3CA
Panax ginseng C.A. Meyer	Panaxacol	PIK3CB
Panax ginseng C.A. Meyer	Panaxacol	PIK3CD
Panax ginseng C.A. Meyer	Panaxacol	PIK3CG
Panax ginseng C.A. Meyer	Panaxacol	PRKCA
Panax ginseng C.A. Meyer	Panaxacol	PRKCD
Panax ginseng C.A. Meyer	Panaxacol	PSEN2
Panax ginseng C.A. Meyer	Panaxacol	PTGS2
Panax ginseng C.A. Meyer	Panaxacol	ROCK1
Panax ginseng C.A. Meyer	Panaxacol	ROCK2
Panax ginseng C.A. Meyer	Panaxacol	SCN9A
Panax ginseng C.A. Meyer	Panaxacol	SELP
Panax ginseng C.A. Meyer	Panaxacol	SYK
Panax ginseng C.A. Meyer	Panaxacol	TYMP
Panax ginseng C.A. Meyer	Panaxatriol	ACKR3
Panax ginseng C.A. Meyer	Panaxatriol	ADORA1
Panax ginseng C.A. Meyer	Panaxatriol	ADORA2A
Panax ginseng C.A. Meyer	Panaxatriol	ALK
Panax ginseng C.A. Meyer	Panaxatriol	APP
Panax ginseng C.A. Meyer	Panaxatriol	BACE1
Panax ginseng C.A. Meyer	Panaxatriol	CAPN2
Panax ginseng C.A. Meyer	Panaxatriol	CCR1
Panax ginseng C.A. Meyer	Panaxatriol	CDK2

Panax ginseng C.A. Meyer	Panaxatriol	CHRM1
Panax ginseng C.A. Meyer	Panaxatriol	CHRM2
Panax ginseng C.A. Meyer	Panaxatriol	CNR1
Panax ginseng C.A. Meyer	Panaxatriol	CNR2
Panax ginseng C.A. Meyer	Panaxatriol	CRHR1
Panax ginseng C.A. Meyer	Panaxatriol	DRD2
Panax ginseng C.A. Meyer	Panaxatriol	EGFR
Panax ginseng C.A. Meyer	Panaxatriol	F10
Panax ginseng C.A. Meyer	Panaxatriol	FAAH
Panax ginseng C.A. Meyer	Panaxatriol	GRIA2
Panax ginseng C.A. Meyer	Panaxatriol	GRIN2A
Panax ginseng C.A. Meyer	Panaxatriol	GRM1
Panax ginseng C.A. Meyer	Panaxatriol	GSK3B
Panax ginseng C.A. Meyer	Panaxatriol	ITGAL
Panax ginseng C.A. Meyer	Panaxatriol	JAK2
Panax ginseng C.A. Meyer	Panaxatriol	KDR
Panax ginseng C.A. Meyer	Panaxatriol	KIT
Panax ginseng C.A. Meyer	Panaxatriol	MAPK1
Panax ginseng C.A. Meyer	Panaxatriol	MAPK14
Panax ginseng C.A. Meyer	Panaxatriol	MAPK8
Panax ginseng C.A. Meyer	Panaxatriol	MDM2
Panax ginseng C.A. Meyer	Panaxatriol	MTOR
Panax ginseng C.A. Meyer	Panaxatriol	NOS2
Panax ginseng C.A. Meyer	Panaxatriol	NR3C1
Panax ginseng C.A. Meyer	Panaxatriol	NR3C2
Panax ginseng C.A. Meyer	Panaxatriol	NTRK1
Panax ginseng C.A. Meyer	Panaxatriol	PDE10A
Panax ginseng C.A. Meyer	Panaxatriol	PDE2A
Panax ginseng C.A. Meyer	Panaxatriol	PDE3A
Panax ginseng C.A. Meyer	Panaxatriol	PDGFRB
Panax ginseng C.A. Meyer	Panaxatriol	PGR
Panax ginseng C.A. Meyer	Panaxatriol	PIK3CA
Panax ginseng C.A. Meyer	Panaxatriol	PIK3CB
Panax ginseng C.A. Meyer	Panaxatriol	PIK3CD
Panax ginseng C.A. Meyer	Panaxatriol	PIK3CG
Panax ginseng C.A. Meyer	Panaxatriol	PRKCB
Panax ginseng C.A. Meyer	Panaxatriol	PTGS1
Panax ginseng C.A. Meyer	Panaxatriol	PTGS2
Panax ginseng C.A. Meyer	Panaxatriol	ROCK1
Panax ginseng C.A. Meyer	Panaxatriol	ROCK2
Panax ginseng C.A. Meyer	Panaxatriol	S1PR1
Panax ginseng C.A. Meyer	Panaxatriol	SHH
Panax ginseng C.A. Meyer	Panaxatriol	SLC6A2
Panax ginseng C.A. Meyer	Panaxatriol	SLC6A3

Panax ginseng C.A. Meyer	Panaxatriol	SYK
Panax ginseng C.A. Meyer	Panaxatriol	TACR1
Panax ginseng C.A. Meyer	Pancratistatin	ABCB1
Panax ginseng C.A. Meyer	Pancratistatin	ABL1
Panax ginseng C.A. Meyer	Pancratistatin	ACHE
Panax ginseng C.A. Meyer	Pancratistatin	ADORA1
Panax ginseng C.A. Meyer	Pancratistatin	ADORA2A
Panax ginseng C.A. Meyer	Pancratistatin	ADRA1A
Panax ginseng C.A. Meyer	Pancratistatin	ALOX5
Panax ginseng C.A. Meyer	Pancratistatin	ALPL
Panax ginseng C.A. Meyer	Pancratistatin	AR
Panax ginseng C.A. Meyer	Pancratistatin	CA1
Panax ginseng C.A. Meyer	Pancratistatin	CASP1
Panax ginseng C.A. Meyer	Pancratistatin	CASP3
Panax ginseng C.A. Meyer	Pancratistatin	CASP8
Panax ginseng C.A. Meyer	Pancratistatin	CCR1
Panax ginseng C.A. Meyer	Pancratistatin	CDK2
Panax ginseng C.A. Meyer	Pancratistatin	CHUK
Panax ginseng C.A. Meyer	Pancratistatin	CNR1
Panax ginseng C.A. Meyer	Pancratistatin	CNR2
Panax ginseng C.A. Meyer	Pancratistatin	CTSB
Panax ginseng C.A. Meyer	Pancratistatin	CTSL
Panax ginseng C.A. Meyer	Pancratistatin	EGFR
Panax ginseng C.A. Meyer	Pancratistatin	ESR2
Panax ginseng C.A. Meyer	Pancratistatin	GRIA2
Panax ginseng C.A. Meyer	Pancratistatin	GRIN2A
Panax ginseng C.A. Meyer	Pancratistatin	GRM1
Panax ginseng C.A. Meyer	Pancratistatin	GSK3B
Panax ginseng C.A. Meyer	Pancratistatin	HRH3
Panax ginseng C.A. Meyer	Pancratistatin	ITGAL
Panax ginseng C.A. Meyer	Pancratistatin	JAK2
Panax ginseng C.A. Meyer	Pancratistatin	MAP3K14
Panax ginseng C.A. Meyer	Pancratistatin	MAP3K5
Panax ginseng C.A. Meyer	Pancratistatin	MAPK14
Panax ginseng C.A. Meyer	Pancratistatin	MDM2
Panax ginseng C.A. Meyer	Pancratistatin	MTOR
Panax ginseng C.A. Meyer	Pancratistatin	NAMPT
Panax ginseng C.A. Meyer	Pancratistatin	NR3C2
Panax ginseng C.A. Meyer	Pancratistatin	NTRK1
Panax ginseng C.A. Meyer	Pancratistatin	OPRM1
Panax ginseng C.A. Meyer	Pancratistatin	PDE10A
Panax ginseng C.A. Meyer	Pancratistatin	PDE2A
Panax ginseng C.A. Meyer	Pancratistatin	PGK1
Panax ginseng C.A. Meyer	Pancratistatin	PGR

Panax ginseng C.A. Meyer	Pancratistatin	PLA2G7
Panax ginseng C.A. Meyer	Pancratistatin	PPARA
Panax ginseng C.A. Meyer	Pancratistatin	PPARD
Panax ginseng C.A. Meyer	Pancratistatin	PRKCA
Panax ginseng C.A. Meyer	Pancratistatin	PRKCD
Panax ginseng C.A. Meyer	Pancratistatin	S1PR1
Panax ginseng C.A. Meyer	Pancratistatin	SCN9A
Panax ginseng C.A. Meyer	Pancratistatin	SYK
Panax ginseng C.A. Meyer	Pancratistatin	TBK1
Panax ginseng C.A. Meyer	Pancratistatin	TGFBR1
Panax ginseng C.A. Meyer	Pandamine	OPRM1
Panax ginseng C.A. Meyer	Pandamine	REN
Panax ginseng C.A. Meyer	Pandamine	SLC6A3
Panax ginseng C.A. Meyer	Pandamine	XIAP
Panax ginseng C.A. Meyer	Patchouli Alcohol	AR
Panax ginseng C.A. Meyer	Patchouli Alcohol	CA1
Panax ginseng C.A. Meyer	Patchouli Alcohol	CA4
Panax ginseng C.A. Meyer	Patchouli Alcohol	ESR1
Panax ginseng C.A. Meyer	Patchouli Alcohol	ESR2
Panax ginseng C.A. Meyer	Patchouli Alcohol	G6PD
Panax ginseng C.A. Meyer	Patchouli Alcohol	HSD11B1
Panax ginseng C.A. Meyer	Patchouli Alcohol	NR1H4
Panax ginseng C.A. Meyer	Patchouli Alcohol	SHH
Panax ginseng C.A. Meyer	Pentadecanoic Acid	ALOX12
Panax ginseng C.A. Meyer	Pentadecanoic Acid	AR
Panax ginseng C.A. Meyer	Pentadecanoic Acid	CA1
Panax ginseng C.A. Meyer	Pentadecanoic Acid	CHRNA7
Panax ginseng C.A. Meyer	Pentadecanoic Acid	FABP3
Panax ginseng C.A. Meyer	Pentadecanoic Acid	G6PD
Panax ginseng C.A. Meyer	Pentadecanoic Acid	HMGCR
Panax ginseng C.A. Meyer	Pentadecanoic Acid	HSD11B1
Panax ginseng C.A. Meyer	Pentadecanoic Acid	MAPK1
Panax ginseng C.A. Meyer	Pentadecanoic Acid	NR1H4
Panax ginseng C.A. Meyer	Pentadecanoic Acid	PLA2G4A
Panax ginseng C.A. Meyer	Pentadecanoic Acid	PLG
Panax ginseng C.A. Meyer	Pentadecanoic Acid	PPARA
Panax ginseng C.A. Meyer	Pentadecanoic Acid	PPARD
Panax ginseng C.A. Meyer	Pentadecanoic Acid	PPARG
Panax ginseng C.A. Meyer	Pentadecanoic Acid	PTPN1
Panax ginseng C.A. Meyer	Pentadecanoic Acid	RARA
Panax ginseng C.A. Meyer	Pentadecanoic Acid	RBP4
Panax ginseng C.A. Meyer	Pentadecanoic Acid	RXRA
Panax ginseng C.A. Meyer	Pentadecanoic Acid	TBXA2R
Panax ginseng C.A. Meyer	Pentadecanoic Acid	VDR

Panax ginseng C.A. Meyer	Protopanaxadiol	ACHE
Panax ginseng C.A. Meyer	Protopanaxadiol	AR
Panax ginseng C.A. Meyer	Protopanaxadiol	BCHE
Panax ginseng C.A. Meyer	Protopanaxadiol	CCR1
Panax ginseng C.A. Meyer	Protopanaxadiol	CHRM2
Panax ginseng C.A. Meyer	Protopanaxadiol	CXCR3
Panax ginseng C.A. Meyer	Protopanaxadiol	CYP2C19
Panax ginseng C.A. Meyer	Protopanaxadiol	ESR1
Panax ginseng C.A. Meyer	Protopanaxadiol	ESR2
Panax ginseng C.A. Meyer	Protopanaxadiol	F10
Panax ginseng C.A. Meyer	Protopanaxadiol	HMGCR
Panax ginseng C.A. Meyer	Protopanaxadiol	HSD11B1
Panax ginseng C.A. Meyer	Protopanaxadiol	MAPK14
Panax ginseng C.A. Meyer	Protopanaxadiol	MDM2
Panax ginseng C.A. Meyer	Protopanaxadiol	P2RY12
Panax ginseng C.A. Meyer	Protopanaxadiol	PDE10A
Panax ginseng C.A. Meyer	Protopanaxadiol	PRKCB
Panax ginseng C.A. Meyer	Protopanaxadiol	PRKCD
Panax ginseng C.A. Meyer	Protopanaxadiol	PRKCE
Panax ginseng C.A. Meyer	Protopanaxadiol	PRKCG
Panax ginseng C.A. Meyer	Protopanaxadiol	PTPN1
Panax ginseng C.A. Meyer	Protopanaxadiol	SLC6A2
Panax ginseng C.A. Meyer	Protopanaxadiol	SLC6A4
Panax ginseng C.A. Meyer	Protopanaxadiol	VDR
Panax ginseng C.A. Meyer	Protopanaxatriol	ACHE
Panax ginseng C.A. Meyer	Protopanaxatriol	ADORA1
Panax ginseng C.A. Meyer	Protopanaxatriol	ADORA2A
Panax ginseng C.A. Meyer	Protopanaxatriol	ALK
Panax ginseng C.A. Meyer	Protopanaxatriol	ALPL
Panax ginseng C.A. Meyer	Protopanaxatriol	AR
Panax ginseng C.A. Meyer	Protopanaxatriol	BCHE
Panax ginseng C.A. Meyer	Protopanaxatriol	CAPN2
Panax ginseng C.A. Meyer	Protopanaxatriol	CCR1
Panax ginseng C.A. Meyer	Protopanaxatriol	CHRM2
Panax ginseng C.A. Meyer	Protopanaxatriol	CRHR1
Panax ginseng C.A. Meyer	Protopanaxatriol	CTSD
Panax ginseng C.A. Meyer	Protopanaxatriol	CYP2C19
Panax ginseng C.A. Meyer	Protopanaxatriol	CYP2C9
Panax ginseng C.A. Meyer	Protopanaxatriol	CYP2D6
Panax ginseng C.A. Meyer	Protopanaxatriol	CYP3A4
Panax ginseng C.A. Meyer	Protopanaxatriol	DRD1
Panax ginseng C.A. Meyer	Protopanaxatriol	DRD2
Panax ginseng C.A. Meyer	Protopanaxatriol	DRD3
Panax ginseng C.A. Meyer	Protopanaxatriol	ESR1

Panax ginseng C.A. Meyer	Protopanaxatriol	ESR2
Panax ginseng C.A. Meyer	Protopanaxatriol	F2R
Panax ginseng C.A. Meyer	Protopanaxatriol	FGFR1
Panax ginseng C.A. Meyer	Protopanaxatriol	HMGCR
Panax ginseng C.A. Meyer	Protopanaxatriol	HSD11B1
Panax ginseng C.A. Meyer	Protopanaxatriol	HSP90AA1
Panax ginseng C.A. Meyer	Protopanaxatriol	HTR1A
Panax ginseng C.A. Meyer	Protopanaxatriol	INSR
Panax ginseng C.A. Meyer	Protopanaxatriol	ITGAL
Panax ginseng C.A. Meyer	Protopanaxatriol	JAK2
Panax ginseng C.A. Meyer	Protopanaxatriol	KDR
Panax ginseng C.A. Meyer	Protopanaxatriol	KIT
Panax ginseng C.A. Meyer	Protopanaxatriol	MAPK14
Panax ginseng C.A. Meyer	Protopanaxatriol	MAPK8
Panax ginseng C.A. Meyer	Protopanaxatriol	MDM2
Panax ginseng C.A. Meyer	Protopanaxatriol	MTOR
Panax ginseng C.A. Meyer	Protopanaxatriol	OPRM1
Panax ginseng C.A. Meyer	Protopanaxatriol	PDE10A
Panax ginseng C.A. Meyer	Protopanaxatriol	PDE2A
Panax ginseng C.A. Meyer	Protopanaxatriol	PDE5A
Panax ginseng C.A. Meyer	Protopanaxatriol	PIK3CA
Panax ginseng C.A. Meyer	Protopanaxatriol	PIK3CB
Panax ginseng C.A. Meyer	Protopanaxatriol	PIK3CD
Panax ginseng C.A. Meyer	Protopanaxatriol	PIK3CG
Panax ginseng C.A. Meyer	Protopanaxatriol	PSEN2
Panax ginseng C.A. Meyer	Protopanaxatriol	PTGS1
Panax ginseng C.A. Meyer	Protopanaxatriol	PTGS2
Panax ginseng C.A. Meyer	Protopanaxatriol	PTPN1
Panax ginseng C.A. Meyer	Protopanaxatriol	REN
Panax ginseng C.A. Meyer	Protopanaxatriol	SCN9A
Panax ginseng C.A. Meyer	Protopanaxatriol	SLC6A2
Panax ginseng C.A. Meyer	Protopanaxatriol	SLC6A4
Panax ginseng C.A. Meyer	Protopanaxatriol	STAT3
Panax ginseng C.A. Meyer	Protopanaxatriol	VDR
Panax ginseng C.A. Meyer	Protopine	ADRA1A
Panax ginseng C.A. Meyer	Protopine	APP
Panax ginseng C.A. Meyer	Protopine	CDK2
Panax ginseng C.A. Meyer	Protopine	DRD1
Panax ginseng C.A. Meyer	Protopine	DRD2
Panax ginseng C.A. Meyer	Protopine	DRD3
Panax ginseng C.A. Meyer	Protopine	ERN1
Panax ginseng C.A. Meyer	Protopine	F3
Panax ginseng C.A. Meyer	Protopine	HTR1A
Panax ginseng C.A. Meyer	Protopine	JAK2

Panax ginseng C.A. Meyer	Protopine	LRRK2
Panax ginseng C.A. Meyer	Protopine	MAPK8
Panax ginseng C.A. Meyer	Protopine	NEK1
Panax ginseng C.A. Meyer	Protopine	PIK3CA
Panax ginseng C.A. Meyer	Protopine	PIK3CB
Panax ginseng C.A. Meyer	Protopine	PIK3CD
Panax ginseng C.A. Meyer	Protopine	PTGS2
Panax ginseng C.A. Meyer	Protopine	SIGMAR1
Panax ginseng C.A. Meyer	Protopine	SLC6A3
Panax ginseng C.A. Meyer	Protopine	TYMS
Panax ginseng C.A. Meyer	Putrescine	CA4
Panax ginseng C.A. Meyer	Pyrrole-2-Aldehyde	ACHE
Panax ginseng C.A. Meyer	Pyrrole-2-Aldehyde	BCHE
Panax ginseng C.A. Meyer	Pyrrole-2-Aldehyde	IDO1
Panax ginseng C.A. Meyer	Pyrrole-2-Aldehyde	PTPN1
Panax ginseng C.A. Meyer	Pyrrole-2-Aldehyde	PTPRC
Panax ginseng C.A. Meyer	Ramalic Acid	ACP1
Panax ginseng C.A. Meyer	Ramalic Acid	CCNA2
Panax ginseng C.A. Meyer	Ramalic Acid	CDK1
Panax ginseng C.A. Meyer	Ramalic Acid	CDK2
Panax ginseng C.A. Meyer	Ramalic Acid	CDK4
Panax ginseng C.A. Meyer	Ramalic Acid	CMA1
Panax ginseng C.A. Meyer	Ramalic Acid	CTNNB1
Panax ginseng C.A. Meyer	Ramalic Acid	CXCR2
Panax ginseng C.A. Meyer	Ramalic Acid	GSK3B
Panax ginseng C.A. Meyer	Ramalic Acid	HMGCR
Panax ginseng C.A. Meyer	Ramalic Acid	IGFBP3
Panax ginseng C.A. Meyer	Ramalic Acid	ITGB1
Panax ginseng C.A. Meyer	Ramalic Acid	JAK2
Panax ginseng C.A. Meyer	Ramalic Acid	LPAR1
Panax ginseng C.A. Meyer	Ramalic Acid	MAPK1
Panax ginseng C.A. Meyer	Ramalic Acid	MAPK10
Panax ginseng C.A. Meyer	Ramalic Acid	MAPK14
Panax ginseng C.A. Meyer	Ramalic Acid	MAPK8
Panax ginseng C.A. Meyer	Ramalic Acid	MMP1
Panax ginseng C.A. Meyer	Ramalic Acid	MMP2
Panax ginseng C.A. Meyer	Ramalic Acid	MMP9
Panax ginseng C.A. Meyer	Ramalic Acid	PDE3A
Panax ginseng C.A. Meyer	Ramalic Acid	PIK3CG
Panax ginseng C.A. Meyer	Ramalic Acid	PTGIR
Panax ginseng C.A. Meyer	Ramalic Acid	PTPN1
Panax ginseng C.A. Meyer	Rhamnose	CDK1
Panax ginseng C.A. Meyer	Rhamnose	FGF1
Panax ginseng C.A. Meyer	Rhamnose	FGF2

Panax ginseng C.A. Meyer	Rhamnose	FOLH1
Panax ginseng C.A. Meyer	Rhamnose	GBA
Panax ginseng C.A. Meyer	Rhamnose	HPSE
Panax ginseng C.A. Meyer	Rhamnose	HSP90AA1
Panax ginseng C.A. Meyer	Rhamnose	LGALS3
Panax ginseng C.A. Meyer	Rhamnose	PSEN2
Panax ginseng C.A. Meyer	Rhamnose	VEGFA
Panax ginseng C.A. Meyer	Spermidine	CA1
Panax ginseng C.A. Meyer	Spermidine	CA4
Panax ginseng C.A. Meyer	Spermidine	CASP2
Panax ginseng C.A. Meyer	Spermidine	CHRNA4
Panax ginseng C.A. Meyer	Spermine	CA1
Panax ginseng C.A. Meyer	Spermine	CA4
Panax ginseng C.A. Meyer	Spermine	CASP2
Panax ginseng C.A. Meyer	Spermine	CHRNA4
Panax ginseng C.A. Meyer	Tauremisin	ABCB1
Panax ginseng C.A. Meyer	Tauremisin	ABCC9
Panax ginseng C.A. Meyer	Tauremisin	AKT1
Panax ginseng C.A. Meyer	Tauremisin	ALOX5
Panax ginseng C.A. Meyer	Tauremisin	AOC3
Panax ginseng C.A. Meyer	Tauremisin	AR
Panax ginseng C.A. Meyer	Tauremisin	BCL2L1
Panax ginseng C.A. Meyer	Tauremisin	CASP1
Panax ginseng C.A. Meyer	Tauremisin	CDK5R1
Panax ginseng C.A. Meyer	Tauremisin	CREBBP
Panax ginseng C.A. Meyer	Tauremisin	CXCR2
Panax ginseng C.A. Meyer	Tauremisin	CYP11B2
Panax ginseng C.A. Meyer	Tauremisin	CYP2C19
Panax ginseng C.A. Meyer	Tauremisin	CYP2C9
Panax ginseng C.A. Meyer	Tauremisin	F2
Panax ginseng C.A. Meyer	Tauremisin	F2RL1
Panax ginseng C.A. Meyer	Tauremisin	FLT1
Panax ginseng C.A. Meyer	Tauremisin	GSK3B
Panax ginseng C.A. Meyer	Tauremisin	HMGCR
Panax ginseng C.A. Meyer	Tauremisin	HMOX1
Panax ginseng C.A. Meyer	Tauremisin	HSD11B1
Panax ginseng C.A. Meyer	Tauremisin	JAK2
Panax ginseng C.A. Meyer	Tauremisin	KIT
Panax ginseng C.A. Meyer	Tauremisin	LRRK2
Panax ginseng C.A. Meyer	Tauremisin	MAP2K1
Panax ginseng C.A. Meyer	Tauremisin	MAPK1
Panax ginseng C.A. Meyer	Tauremisin	NOS2
Panax ginseng C.A. Meyer	Tauremisin	NR3C1
Panax ginseng C.A. Meyer	Tauremisin	NR3C2

Panax ginseng C.A. Meyer	Tauremisin	PARP1
Panax ginseng C.A. Meyer	Tauremisin	PDCD4
Panax ginseng C.A. Meyer	Tauremisin	PDE10A
Panax ginseng C.A. Meyer	Tauremisin	PGR
Panax ginseng C.A. Meyer	Tauremisin	PLA2G2A
Panax ginseng C.A. Meyer	Tauremisin	PPARG
Panax ginseng C.A. Meyer	Tauremisin	PREP
Panax ginseng C.A. Meyer	Tauremisin	PRKCA
Panax ginseng C.A. Meyer	Tauremisin	PRKCD
Panax ginseng C.A. Meyer	Tauremisin	PTGS2
Panax ginseng C.A. Meyer	Tauremisin	PTPN1
Panax ginseng C.A. Meyer	Tauremisin	PTPN11
Panax ginseng C.A. Meyer	Tauremisin	SLC6A3
Panax ginseng C.A. Meyer	Tauremisin	TBXA2R
Panax ginseng C.A. Meyer	Tauremisin	TERT
Panax ginseng C.A. Meyer	Tauremisin	TYMS
Panax ginseng C.A. Meyer	Tauremisin	VDR
Panax ginseng C.A. Meyer	Tridecanoic Acid	ADRA2B
Panax ginseng C.A. Meyer	Tridecanoic Acid	AKR1B1
Panax ginseng C.A. Meyer	Tridecanoic Acid	AR
Panax ginseng C.A. Meyer	Tridecanoic Acid	CA1
Panax ginseng C.A. Meyer	Tridecanoic Acid	CHRNA7
Panax ginseng C.A. Meyer	Tridecanoic Acid	CMA1
Panax ginseng C.A. Meyer	Tridecanoic Acid	CYP1A2
Panax ginseng C.A. Meyer	Tridecanoic Acid	EDNRA
Panax ginseng C.A. Meyer	Tridecanoic Acid	FABP3
Panax ginseng C.A. Meyer	Tridecanoic Acid	FOLH1
Panax ginseng C.A. Meyer	Tridecanoic Acid	G6PD
Panax ginseng C.A. Meyer	Tridecanoic Acid	HMGCR
Panax ginseng C.A. Meyer	Tridecanoic Acid	HSD11B1
Panax ginseng C.A. Meyer	Tridecanoic Acid	MAPK1
Panax ginseng C.A. Meyer	Tridecanoic Acid	MAPK14
Panax ginseng C.A. Meyer	Tridecanoic Acid	MMP12
Panax ginseng C.A. Meyer	Tridecanoic Acid	MMP2
Panax ginseng C.A. Meyer	Tridecanoic Acid	MMP9
Panax ginseng C.A. Meyer	Tridecanoic Acid	NR1H4
Panax ginseng C.A. Meyer	Tridecanoic Acid	NR3C2
Panax ginseng C.A. Meyer	Tridecanoic Acid	PGR
Panax ginseng C.A. Meyer	Tridecanoic Acid	PLA2G4A
Panax ginseng C.A. Meyer	Tridecanoic Acid	PLG
Panax ginseng C.A. Meyer	Tridecanoic Acid	PPARA
Panax ginseng C.A. Meyer	Tridecanoic Acid	PPARD
Panax ginseng C.A. Meyer	Tridecanoic Acid	PPARG
Panax ginseng C.A. Meyer	Tridecanoic Acid	PTPN1

Panax ginseng C.A. Meyer	Tridecanoic Acid	PTPRC
Panax ginseng C.A. Meyer	Tridecanoic Acid	RARA
Panax ginseng C.A. Meyer	Tridecanoic Acid	RBP4
Panax ginseng C.A. Meyer	Tridecanoic Acid	RXRA
Panax ginseng C.A. Meyer	Tridecanoic Acid	TBXA2R
Panax ginseng C.A. Meyer	Tridecanoic Acid	VDR
Panax ginseng C.A. Meyer	Trifolirhizin	ADORA1
Panax ginseng C.A. Meyer	Trifolirhizin	ALDH2
Panax ginseng C.A. Meyer	Trifolirhizin	IL2
Panax ginseng C.A. Meyer	Trifolirhizin	PTGS1
Panax ginseng C.A. Meyer	Trifolirhizin	PTGS2
Panax ginseng C.A. Meyer	Trifolirhizin	TYR
Panax ginseng C.A. Meyer	Vitamin B5	AKR1B1
Panax ginseng C.A. Meyer	Vitamin B5	CA1
Panax ginseng C.A. Meyer	Vitamin B5	DNMT3B
Panax ginseng C.A. Meyer	Vitamin B5	ECE1
Panax ginseng C.A. Meyer	Vitamin B5	FOLH1
Panax ginseng C.A. Meyer	Vitamin B5	SLC13A5
Panax ginseng C.A. Meyer	widdrol	ACHE
Panax ginseng C.A. Meyer	widdrol	AR
Panax ginseng C.A. Meyer	widdrol	BCHE
Panax ginseng C.A. Meyer	widdrol	CHRM2
Panax ginseng C.A. Meyer	widdrol	CYP2C19
Panax ginseng C.A. Meyer	widdrol	ESR1
Panax ginseng C.A. Meyer	widdrol	ESR2
Panax ginseng C.A. Meyer	widdrol	G6PD
Panax ginseng C.A. Meyer	widdrol	HMGCR
Panax ginseng C.A. Meyer	widdrol	HSD11B1
Panax ginseng C.A. Meyer	widdrol	IDO1
Panax ginseng C.A. Meyer	widdrol	PPARA
Panax ginseng C.A. Meyer	widdrol	PPARD
Panax ginseng C.A. Meyer	widdrol	PTPN1
Panax ginseng C.A. Meyer	widdrol	PTPN6
Panax ginseng C.A. Meyer	widdrol	SLC6A2
Panax ginseng C.A. Meyer	widdrol	SLC6A4
Panax ginseng C.A. Meyer	widdrol	SREBF2
Panax ginseng C.A. Meyer	widdrol	VDR
Panax ginseng C.A. Meyer	α-cadinol	ACHE
Panax ginseng C.A. Meyer	α-cadinol	ACP1
Panax ginseng C.A. Meyer	α-cadinol	AR
Panax ginseng C.A. Meyer	α-cadinol	BCHE
Panax ginseng C.A. Meyer	α-cadinol	CHRM2
Panax ginseng C.A. Meyer	α-cadinol	CYP2C19
Panax ginseng C.A. Meyer	α-cadinol	ESR1

Panax ginseng C.A. Meyer	α-cadinol	ESR2
Panax ginseng C.A. Meyer	α-cadinol	FABP3
Panax ginseng C.A. Meyer	α-cadinol	G6PD
Panax ginseng C.A. Meyer	α-cadinol	HMGCR
Panax ginseng C.A. Meyer	α-cadinol	HSD11B1
Panax ginseng C.A. Meyer	α-cadinol	PLA2G1B
Panax ginseng C.A. Meyer	α-cadinol	PPARA
Panax ginseng C.A. Meyer	α-cadinol	PPARD
Panax ginseng C.A. Meyer	α-cadinol	PREP
Panax ginseng C.A. Meyer	α-cadinol	PTPN1
Panax ginseng C.A. Meyer	α-cadinol	PTPN11
Panax ginseng C.A. Meyer	α-cadinol	PTPN6
Panax ginseng C.A. Meyer	α-cadinol	SLC6A2
Panax ginseng C.A. Meyer	α-cadinol	SLC6A4
Panax ginseng C.A. Meyer	α-cadinol	SREBF2
Panax ginseng C.A. Meyer	α-cadinol	TERT
Panax ginseng C.A. Meyer	α-cedrol	AR
Panax ginseng C.A. Meyer	α-cedrol	CA1
Panax ginseng C.A. Meyer	α-cedrol	CA4
Panax ginseng C.A. Meyer	α-cedrol	ESR1
Panax ginseng C.A. Meyer	α-cedrol	ESR2
Panax ginseng C.A. Meyer	α-cedrol	G6PD
Panax ginseng C.A. Meyer	α-cedrol	HSD11B1
Panax ginseng C.A. Meyer	α-cedrol	IDO1
Panax ginseng C.A. Meyer	α-cedrol	NR1H4
Panax ginseng C.A. Meyer	α-cedrol	SHH
Rhizoma coptidis	(R)-Canadine	ADRA1A
Rhizoma coptidis	(R)-Canadine	ADRB2
Rhizoma coptidis	(R)-Canadine	CALB2
Rhizoma coptidis	(R)-Canadine	CHRM1
Rhizoma coptidis	(R)-Canadine	CHRM2
Rhizoma coptidis	(R)-Canadine	DRD1
Rhizoma coptidis	(R)-Canadine	F10
Rhizoma coptidis	(R)-Canadine	HTR2A
Rhizoma coptidis	(R)-Canadine	HTR2C
Rhizoma coptidis	(R)-Canadine	HTR3A
Rhizoma coptidis	(R)-Canadine	KCNMA1
Rhizoma coptidis	(R)-Canadine	OPRM1
Rhizoma coptidis	(R)-Canadine	PDE10A
Rhizoma coptidis	(R)-Canadine	PRKACA
Rhizoma coptidis	(R)-Canadine	PTGS1
Rhizoma coptidis	(R)-Canadine	PTGS2
Rhizoma coptidis	(R)-Canadine	RXRA
Rhizoma coptidis	(R)-Canadine	SLC6A2

Rhizoma coptidis	(R)-Canadine	SLC6A3
Rhizoma coptidis	(R)-Canadine	SLC6A4
Rhizoma coptidis	berberine	ADRB2
Rhizoma coptidis	berberine	AR
Rhizoma coptidis	berberine	CALB2
Rhizoma coptidis	berberine	ESR1
Rhizoma coptidis	berberine	F10
Rhizoma coptidis	berberine	NOS2
Rhizoma coptidis	berberine	NOS3
Rhizoma coptidis	berberine	PDE10A
Rhizoma coptidis	berberine	PRKACA
Rhizoma coptidis	berberine	PTGS1
Rhizoma coptidis	berberine	PTGS2
Rhizoma coptidis	berberine	RXRA
Rhizoma coptidis	berberrubine	AR
Rhizoma coptidis	berberrubine	CALB2
Rhizoma coptidis	berberrubine	ESR1
Rhizoma coptidis	berberrubine	NOS2
Rhizoma coptidis	berberrubine	NOS3
Rhizoma coptidis	berberrubine	PRKACA
Rhizoma coptidis	berberrubine	PTGS1
Rhizoma coptidis	berberrubine	PTGS2
Rhizoma coptidis	berberrubine	RXRA
Rhizoma coptidis	Berlambine	ADRB2
Rhizoma coptidis	Berlambine	AR
Rhizoma coptidis	Berlambine	CALB2
Rhizoma coptidis	Berlambine	F10
Rhizoma coptidis	Berlambine	F7
Rhizoma coptidis	Berlambine	KCNMA1
Rhizoma coptidis	Berlambine	NOS2
Rhizoma coptidis	Berlambine	NOS3
Rhizoma coptidis	Berlambine	PRKACA
Rhizoma coptidis	Berlambine	PTGS1
Rhizoma coptidis	Berlambine	PTGS2
Rhizoma coptidis	Berlambine	RXRA
Rhizoma coptidis	Chlorogenic Acid	ABCB1
Rhizoma coptidis	Chlorogenic Acid	AKR1B1
Rhizoma coptidis	Chlorogenic Acid	APP
Rhizoma coptidis	Chlorogenic Acid	BACE1
Rhizoma coptidis	Chlorogenic Acid	CA1
Rhizoma coptidis	Chlorogenic Acid	EDNRA
Rhizoma coptidis	Chlorogenic Acid	ELANE
Rhizoma coptidis	Chlorogenic Acid	MMP12
Rhizoma coptidis	Chlorogenic Acid	MMP2

Rhizoma coptidis	Chlorogenic Acid	PDE5A
Rhizoma coptidis	Chlorogenic Acid	PRKCA
Rhizoma coptidis	Chlorogenic Acid	PRKCD
Rhizoma coptidis	Columbamine	ACHE
Rhizoma coptidis	Columbamine	ACVRL1
Rhizoma coptidis	Columbamine	ADRA2B
Rhizoma coptidis	Columbamine	AKR1B1
Rhizoma coptidis	Columbamine	ALDH2
Rhizoma coptidis	Columbamine	ALK
Rhizoma coptidis	Columbamine	BCHE
Rhizoma coptidis	Columbamine	BMP4
Rhizoma coptidis	Columbamine	BMPR2
Rhizoma coptidis	Columbamine	BRAF
Rhizoma coptidis	Columbamine	CASP3
Rhizoma coptidis	Columbamine	CDC42
Rhizoma coptidis	Columbamine	CDK2
Rhizoma coptidis	Columbamine	CDK2
Rhizoma coptidis	Columbamine	CDK4
Rhizoma coptidis	Columbamine	CETP
Rhizoma coptidis	Columbamine	CHRM1
Rhizoma coptidis	Columbamine	CHRNA7
Rhizoma coptidis	Columbamine	CYP2D6
Rhizoma coptidis	Columbamine	DYRK1A
Rhizoma coptidis	Columbamine	F3
Rhizoma coptidis	Columbamine	HSD11B1
Rhizoma coptidis	Columbamine	HSP90AA1
Rhizoma coptidis	Columbamine	HTR3A
Rhizoma coptidis	Columbamine	MAP2K1
Rhizoma coptidis	Columbamine	MAPK8
Rhizoma coptidis	Columbamine	MIF
Rhizoma coptidis	Columbamine	MMP1
Rhizoma coptidis	Columbamine	MMP2
Rhizoma coptidis	Columbamine	MMP3
Rhizoma coptidis	Columbamine	MMP9
Rhizoma coptidis	Columbamine	MTOR
Rhizoma coptidis	Columbamine	NR3C2
Rhizoma coptidis	Columbamine	P2RX7
Rhizoma coptidis	Columbamine	PGR
Rhizoma coptidis	Columbamine	PIK3CB
Rhizoma coptidis	Columbamine	PIK3CD
Rhizoma coptidis	Columbamine	PRKCE
Rhizoma coptidis	Columbamine	PSEN2
Rhizoma coptidis	Columbamine	PTPN1
Rhizoma coptidis	Columbamine	RAC1

Rhizoma coptidis	Columbamine	RET
Rhizoma coptidis	Columbamine	SIGMAR1
Rhizoma coptidis	Columbamine	TERT
Rhizoma coptidis	Columbamine	TGFBR2
Rhizoma coptidis	Columbamine	TYMS
Rhizoma coptidis	coptisine	AR
Rhizoma coptidis	coptisine	ESR1
Rhizoma coptidis	coptisine	NOS2
Rhizoma coptidis	coptisine	NOS3
Rhizoma coptidis	coptisine	PTGS1
Rhizoma coptidis	coptisine	PTGS2
Rhizoma coptidis	Corchoroside A_qt	NR3C2
Rhizoma coptidis	epiberberine	AR
Rhizoma coptidis	epiberberine	ESR1
Rhizoma coptidis	epiberberine	NOS2
Rhizoma coptidis	epiberberine	NOS3
Rhizoma coptidis	epiberberine	PDE10A
Rhizoma coptidis	epiberberine	PRKACA
Rhizoma coptidis	epiberberine	PTGS2
Rhizoma coptidis	epiberberine	RXRA
Rhizoma coptidis	Ferulic Acid	ABCB1
Rhizoma coptidis	Ferulic Acid	ADORA1
Rhizoma coptidis	Ferulic Acid	ADORA2A
Rhizoma coptidis	Ferulic Acid	AHR
Rhizoma coptidis	Ferulic Acid	AKR1B1
Rhizoma coptidis	Ferulic Acid	ALOX5
Rhizoma coptidis	Ferulic Acid	APP
Rhizoma coptidis	Ferulic Acid	BACE1
Rhizoma coptidis	Ferulic Acid	CA1
Rhizoma coptidis	Ferulic Acid	CA4
Rhizoma coptidis	Ferulic Acid	CCND1
Rhizoma coptidis	Ferulic Acid	CYP1A2
Rhizoma coptidis	Ferulic Acid	EGFR
Rhizoma coptidis	Ferulic Acid	ESR2
Rhizoma coptidis	Ferulic Acid	F3
Rhizoma coptidis	Ferulic Acid	FYN
Rhizoma coptidis	Ferulic Acid	GLO1
Rhizoma coptidis	Ferulic Acid	HSD11B1
Rhizoma coptidis	Ferulic Acid	MAOB
Rhizoma coptidis	Ferulic Acid	MMP1
Rhizoma coptidis	Ferulic Acid	MMP2
Rhizoma coptidis	Ferulic Acid	MMP9
Rhizoma coptidis	Ferulic Acid	NFE2L2
Rhizoma coptidis	Ferulic Acid	NOS2

Rhizoma coptidis	Ferulic Acid	PARP1
Rhizoma coptidis	Ferulic Acid	PRKCE
Rhizoma coptidis	Ferulic Acid	PTGS1
Rhizoma coptidis	Ferulic Acid	PTGS2
Rhizoma coptidis	Ferulic Acid	PTPN1
Rhizoma coptidis	Ferulic Acid	RELA
Rhizoma coptidis	Ferulic Acid	STAT3
Rhizoma coptidis	Ferulic Acid	TLR4
Rhizoma coptidis	Ferulic Acid	TTR
Rhizoma coptidis	Ferulic Acid	TUBB3
Rhizoma coptidis	Jatrorrhizine	ACHE
Rhizoma coptidis	Jatrorrhizine	ADRA2B
Rhizoma coptidis	Jatrorrhizine	AKR1B1
Rhizoma coptidis	Jatrorrhizine	ALK
Rhizoma coptidis	Jatrorrhizine	BCHE
Rhizoma coptidis	Jatrorrhizine	BRAF
Rhizoma coptidis	Jatrorrhizine	CDC42
Rhizoma coptidis	Jatrorrhizine	CDK2
Rhizoma coptidis	Jatrorrhizine	CDK2
Rhizoma coptidis	Jatrorrhizine	CDK4
Rhizoma coptidis	Jatrorrhizine	CHRM1
Rhizoma coptidis	Jatrorrhizine	CHRNA7
Rhizoma coptidis	Jatrorrhizine	CSF1R
Rhizoma coptidis	Jatrorrhizine	CSNK1D
Rhizoma coptidis	Jatrorrhizine	CYP2D6
Rhizoma coptidis	Jatrorrhizine	DYRK1A
Rhizoma coptidis	Jatrorrhizine	EPHB4
Rhizoma coptidis	Jatrorrhizine	F3
Rhizoma coptidis	Jatrorrhizine	FGFR1
Rhizoma coptidis	Jatrorrhizine	GRIA1
Rhizoma coptidis	Jatrorrhizine	HTR3A
Rhizoma coptidis	Jatrorrhizine	KIT
Rhizoma coptidis	Jatrorrhizine	MAP2K1
Rhizoma coptidis	Jatrorrhizine	MAPK1
Rhizoma coptidis	Jatrorrhizine	MAPK8
Rhizoma coptidis	Jatrorrhizine	MIF
Rhizoma coptidis	Jatrorrhizine	MMP1
Rhizoma coptidis	Jatrorrhizine	MMP2
Rhizoma coptidis	Jatrorrhizine	MMP3
Rhizoma coptidis	Jatrorrhizine	MMP9
Rhizoma coptidis	Jatrorrhizine	MTOR
Rhizoma coptidis	Jatrorrhizine	OGT
Rhizoma coptidis	Jatrorrhizine	P2RX7
Rhizoma coptidis	Jatrorrhizine	PIK3CB

Rhizoma coptidis	Jatrorrhizine	PIK3CD
Rhizoma coptidis	Jatrorrhizine	PIK3CG
Rhizoma coptidis	Jatrorrhizine	PSEN2
Rhizoma coptidis	Jatrorrhizine	PTPN1
Rhizoma coptidis	Jatrorrhizine	RAC1
Rhizoma coptidis	Jatrorrhizine	RAF1
Rhizoma coptidis	Jatrorrhizine	RET
Rhizoma coptidis	Jatrorrhizine	ROCK2
Rhizoma coptidis	Jatrorrhizine	SIGMAR1
Rhizoma coptidis	Jatrorrhizine	SLC1A3
Rhizoma coptidis	Jatrorrhizine	TBXA2R
Rhizoma coptidis	Jatrorrhizine	TNF
Rhizoma coptidis	Javanicin	EGFR
Rhizoma coptidis	Javanicin	GAPDH
Rhizoma coptidis	Javanicin	HSD11B1
Rhizoma coptidis	Javanicin	IDO1
Rhizoma coptidis	Javanicin	MAPK8
Rhizoma coptidis	Javanicin	MMP1
Rhizoma coptidis	Javanicin	MMP3
Rhizoma coptidis	Javanicin	PDE5A
Rhizoma coptidis	Limonin	AKT1
Rhizoma coptidis	Limonin	AR
Rhizoma coptidis	Limonin	BACE1
Rhizoma coptidis	Limonin	C5AR1
Rhizoma coptidis	Limonin	CASP1
Rhizoma coptidis	Limonin	CASP3
Rhizoma coptidis	Limonin	CCR1
Rhizoma coptidis	Limonin	CNR2
Rhizoma coptidis	Limonin	CTSL
Rhizoma coptidis	Limonin	DRD2
Rhizoma coptidis	Limonin	DYRK1A
Rhizoma coptidis	Limonin	F10
Rhizoma coptidis	Limonin	F2
Rhizoma coptidis	Limonin	GSK3B
Rhizoma coptidis	Limonin	HDAC1
Rhizoma coptidis	Limonin	HDAC4
Rhizoma coptidis	Limonin	HMGCR
Rhizoma coptidis	Limonin	HSD11B1
Rhizoma coptidis	Limonin	KDR
Rhizoma coptidis	Limonin	KIT
Rhizoma coptidis	Limonin	LRRK2
Rhizoma coptidis	Limonin	MAP2K1
Rhizoma coptidis	Limonin	MAPK1
Rhizoma coptidis	Limonin	MAPK14

Rhizoma coptidis	Limonin	MDM2
Rhizoma coptidis	Limonin	MMP1
Rhizoma coptidis	Limonin	MMP9
Rhizoma coptidis	Limonin	MTOR
Rhizoma coptidis	Limonin	NR3C1
Rhizoma coptidis	Limonin	OPRM1
Rhizoma coptidis	Limonin	OXTR
Rhizoma coptidis	Limonin	P2RX7
Rhizoma coptidis	Limonin	PDGFRB
Rhizoma coptidis	Limonin	PGR
Rhizoma coptidis	Limonin	PIK3CA
Rhizoma coptidis	Limonin	PIK3CB
Rhizoma coptidis	Limonin	PIK3CD
Rhizoma coptidis	Limonin	PREP
Rhizoma coptidis	Limonin	PSEN2
Rhizoma coptidis	Magnoflorine	ADORA2A
Rhizoma coptidis	Magnoflorine	ADRA1A
Rhizoma coptidis	Magnoflorine	ADRA2A
Rhizoma coptidis	Magnoflorine	ADRA2B
Rhizoma coptidis	Magnoflorine	ADRB2
Rhizoma coptidis	Magnoflorine	AKR1B1
Rhizoma coptidis	Magnoflorine	ALOX12
Rhizoma coptidis	Magnoflorine	AR
Rhizoma coptidis	Magnoflorine	CASP3
Rhizoma coptidis	Magnoflorine	CDK1
Rhizoma coptidis	Magnoflorine	CDK2
Rhizoma coptidis	Magnoflorine	CDK5R1
Rhizoma coptidis	Magnoflorine	CSF1R
Rhizoma coptidis	Magnoflorine	DRD1
Rhizoma coptidis	Magnoflorine	DRD2
Rhizoma coptidis	Magnoflorine	DRD3
Rhizoma coptidis	Magnoflorine	DYRK1A
Rhizoma coptidis	Magnoflorine	EGFR
Rhizoma coptidis	Magnoflorine	EIF2AK2
Rhizoma coptidis	Magnoflorine	ERN1
Rhizoma coptidis	Magnoflorine	F3
Rhizoma coptidis	Magnoflorine	FGFR1
Rhizoma coptidis	Magnoflorine	GSK3B
Rhizoma coptidis	Magnoflorine	HRH2
Rhizoma coptidis	Magnoflorine	HTR1A
Rhizoma coptidis	Magnoflorine	HTR1B
Rhizoma coptidis	Magnoflorine	HTR1D
Rhizoma coptidis	Magnoflorine	HTR2A
Rhizoma coptidis	Magnoflorine	HTR2C

Rhizoma coptidis	Magnoflorine	HTR6
Rhizoma coptidis	Magnoflorine	KDR
Rhizoma coptidis	Magnoflorine	LIPG
Rhizoma coptidis	Magnoflorine	MAP2K1
Rhizoma coptidis	Magnoflorine	MAPK1
Rhizoma coptidis	Magnoflorine	MAPK14
Rhizoma coptidis	Magnoflorine	MAPK3
Rhizoma coptidis	Magnoflorine	MIF
Rhizoma coptidis	Magnoflorine	MMP1
Rhizoma coptidis	Magnoflorine	MMP2
Rhizoma coptidis	Magnoflorine	MMP3
Rhizoma coptidis	Magnoflorine	MMP9
Rhizoma coptidis	Magnoflorine	NR3C2
Rhizoma coptidis	Magnoflorine	OPRM1
Rhizoma coptidis	Magnoflorine	PGR
Rhizoma coptidis	Magnoflorine	PPARG
Rhizoma coptidis	Magnoflorine	PREP
Rhizoma coptidis	Magnoflorine	PRKCG
Rhizoma coptidis	Magnoflorine	RBP4
Rhizoma coptidis	Magnoflorine	RPS6KB1
Rhizoma coptidis	Magnoflorine	SIGMAR1
Rhizoma coptidis	Magnoflorine	SLC6A3
Rhizoma coptidis	Magnoflorine	SLC6A4
Rhizoma coptidis	Magnoflorine	SRC
Rhizoma coptidis	Magnoflorine	STAT3
Rhizoma coptidis	Magnoflorine	TH
Rhizoma coptidis	Magnoflorine	TRPC6
Rhizoma coptidis	Magnoflorine	TSPO
Rhizoma coptidis	Magnograndiolide	GRIA2
Rhizoma coptidis	Obaculactone	ABL1
Rhizoma coptidis	Obaculactone	ADORA1
Rhizoma coptidis	Obaculactone	ADORA2A
Rhizoma coptidis	Obaculactone	AKT1
Rhizoma coptidis	Obaculactone	ALOX5
Rhizoma coptidis	Obaculactone	AOC3
Rhizoma coptidis	Obaculactone	BDKRB2
Rhizoma coptidis	Obaculactone	CDK5R1
Rhizoma coptidis	Obaculactone	CHRNA7
Rhizoma coptidis	Obaculactone	CMA1
Rhizoma coptidis	Obaculactone	CXCR2
Rhizoma coptidis	Obaculactone	DRD1
Rhizoma coptidis	Obaculactone	DYRK1A
Rhizoma coptidis	Obaculactone	EPHB4
Rhizoma coptidis	Obaculactone	FPR2

Rhizoma coptidis	Obaculactone	GRK2
Rhizoma coptidis	Obaculactone	GRM2
Rhizoma coptidis	Obaculactone	GSK3B
Rhizoma coptidis	Obaculactone	HSD11B1
Rhizoma coptidis	Obaculactone	IDO1
Rhizoma coptidis	Obaculactone	JAK2
Rhizoma coptidis	Obaculactone	KDR
Rhizoma coptidis	Obaculactone	MAPK14
Rhizoma coptidis	Obaculactone	MECP2
Rhizoma coptidis	Obaculactone	MMP1
Rhizoma coptidis	Obaculactone	NOS1
Rhizoma coptidis	Obaculactone	NOS2
Rhizoma coptidis	Obaculactone	NTRK1
Rhizoma coptidis	Obaculactone	P2RX7
Rhizoma coptidis	Obaculactone	PARP1
Rhizoma coptidis	Obaculactone	PDE10A
Rhizoma coptidis	Obaculactone	PDE3A
Rhizoma coptidis	Obaculactone	PDGFRB
Rhizoma coptidis	Obaculactone	PGK1
Rhizoma coptidis	Obaculactone	PGR
Rhizoma coptidis	Obaculactone	PIK3CA
Rhizoma coptidis	Obaculactone	PIK3CB
Rhizoma coptidis	Obaculactone	PIK3CD
Rhizoma coptidis	Obaculactone	PIK3CG
Rhizoma coptidis	Obaculactone	PSEN2
Rhizoma coptidis	Obaculactone	PTGS1
Rhizoma coptidis	Obaculactone	PTGS2
Rhizoma coptidis	Obaculactone	QPCT
Rhizoma coptidis	Obaculactone	ROCK1
Rhizoma coptidis	Obaculactone	ROCK2
Rhizoma coptidis	Obaculactone	SCN9A
Rhizoma coptidis	Obaculactone	SRC
Rhizoma coptidis	Obaculactone	SYK
Rhizoma coptidis	Obaculactone	TSPO
Rhizoma coptidis	Obacunoic Acid	ACE
Rhizoma coptidis	Obacunoic Acid	ACE2
Rhizoma coptidis	Obacunoic Acid	ADAM17
Rhizoma coptidis	Obacunoic Acid	ADH5
Rhizoma coptidis	Obacunoic Acid	AGTR1
Rhizoma coptidis	Obacunoic Acid	AKR1B1
Rhizoma coptidis	Obacunoic Acid	BACE1
Rhizoma coptidis	Obacunoic Acid	CASP3
Rhizoma coptidis	Obacunoic Acid	CASP8
Rhizoma coptidis	Obacunoic Acid	CMA1

Rhizoma coptidis	Obacunoic Acid	CREBBP
Rhizoma coptidis	Obacunoic Acid	CTSA
Rhizoma coptidis	Obacunoic Acid	CTSB
Rhizoma coptidis	Obacunoic Acid	CTSL
Rhizoma coptidis	Obacunoic Acid	DPP4
Rhizoma coptidis	Obacunoic Acid	ECE1
Rhizoma coptidis	Obacunoic Acid	EDNRA
Rhizoma coptidis	Obacunoic Acid	EDNRB
Rhizoma coptidis	Obacunoic Acid	ELANE
Rhizoma coptidis	Obacunoic Acid	ESR1
Rhizoma coptidis	Obacunoic Acid	ESR2
Rhizoma coptidis	Obacunoic Acid	FGFR1
Rhizoma coptidis	Obacunoic Acid	FLT1
Rhizoma coptidis	Obacunoic Acid	FOLH1
Rhizoma coptidis	Obacunoic Acid	HMGCR
Rhizoma coptidis	Obacunoic Acid	KDR
Rhizoma coptidis	Obacunoic Acid	KIF11
Rhizoma coptidis	Obacunoic Acid	MAPK8
Rhizoma coptidis	Obacunoic Acid	MDM2
Rhizoma coptidis	Obacunoic Acid	MME
Rhizoma coptidis	Obacunoic Acid	MMP1
Rhizoma coptidis	Obacunoic Acid	MMP2
Rhizoma coptidis	Obacunoic Acid	MMP3
Rhizoma coptidis	Obacunoic Acid	MMP9
Rhizoma coptidis	Obacunoic Acid	OPRM1
Rhizoma coptidis	Obacunoic Acid	PDE5A
Rhizoma coptidis	Obacunoic Acid	PDGFRB
Rhizoma coptidis	Obacunoic Acid	PPARA
Rhizoma coptidis	Obacunoic Acid	PPARD
Rhizoma coptidis	Obacunoic Acid	PPARG
Rhizoma coptidis	Obacunoic Acid	ROCK1
Rhizoma coptidis	Obacunoic Acid	SRC
Rhizoma coptidis	Obacunoic Acid	TBXA2R
Rhizoma coptidis	Obacunoic Acid	TKT
Rhizoma coptidis	Obacunoic Acid	TOP1
Rhizoma coptidis	Obacunoic Acid	TYMS
Rhizoma coptidis	Obamegine	ABCB1
Rhizoma coptidis	Obamegine	ABL1
Rhizoma coptidis	Obamegine	ACHE
Rhizoma coptidis	Obamegine	ACKR3
Rhizoma coptidis	Obamegine	ADRA1A
Rhizoma coptidis	Obamegine	ADRA2A
Rhizoma coptidis	Obamegine	ADRB2
Rhizoma coptidis	Obamegine	ADRB3

Rhizoma coptidis	Obamegine	AKT1
Rhizoma coptidis	Obamegine	BCHE
Rhizoma coptidis	Obamegine	BIRC2
Rhizoma coptidis	Obamegine	BIRC3
Rhizoma coptidis	Obamegine	CDK1
Rhizoma coptidis	Obamegine	CDK2
Rhizoma coptidis	Obamegine	CHRM1
Rhizoma coptidis	Obamegine	CHRM2
Rhizoma coptidis	Obamegine	CREBBP
Rhizoma coptidis	Obamegine	CSF1R
Rhizoma coptidis	Obamegine	DPP4
Rhizoma coptidis	Obamegine	DRD1
Rhizoma coptidis	Obamegine	DRD2
Rhizoma coptidis	Obamegine	DRD3
Rhizoma coptidis	Obamegine	EP300
Rhizoma coptidis	Obamegine	F3
Rhizoma coptidis	Obamegine	FLT1
Rhizoma coptidis	Obamegine	HDAC1
Rhizoma coptidis	Obamegine	HTR1A
Rhizoma coptidis	Obamegine	HTR2A
Rhizoma coptidis	Obamegine	JAK2
Rhizoma coptidis	Obamegine	KDR
Rhizoma coptidis	Obamegine	LRRK2
Rhizoma coptidis	Obamegine	MAP2
Rhizoma coptidis	Obamegine	MAPK10
Rhizoma coptidis	Obamegine	MAPK14
Rhizoma coptidis	Obamegine	MAPK8
Rhizoma coptidis	Obamegine	MMP1
Rhizoma coptidis	Obamegine	MMP12
Rhizoma coptidis	Obamegine	MMP3
Rhizoma coptidis	Obamegine	MTOR
Rhizoma coptidis	Obamegine	OPRM1
Rhizoma coptidis	Obamegine	PDE1A
Rhizoma coptidis	Obamegine	PDE5A
Rhizoma coptidis	Obamegine	PRKCA
Rhizoma coptidis	Obamegine	PRKCB
Rhizoma coptidis	Obamegine	PRKCD
Rhizoma coptidis	Obamegine	PRKCG
Rhizoma coptidis	Obamegine	PRKCZ
Rhizoma coptidis	Obamegine	ROCK1
Rhizoma coptidis	Obamegine	ROCK2
Rhizoma coptidis	Obamegine	SIGMAR1
Rhizoma coptidis	Obamegine	SLC6A3
Rhizoma coptidis	Obamegine	SLC6A4

Rhizoma coptidis	Obamegine	SRC
Rhizoma coptidis	Obamegine	SYK
Rhizoma coptidis	Obamegine	TBXA2R
Rhizoma coptidis	Obamegine	XIAP
Rhizoma coptidis	palmatine	ADRB2
Rhizoma coptidis	palmatine	AR
Rhizoma coptidis	palmatine	CALB2
Rhizoma coptidis	palmatine	CDK2
Rhizoma coptidis	palmatine	ESR1
Rhizoma coptidis	palmatine	ESR2
Rhizoma coptidis	palmatine	F7
Rhizoma coptidis	palmatine	NOS2
Rhizoma coptidis	palmatine	NOS3
Rhizoma coptidis	palmatine	PRKACA
Rhizoma coptidis	palmatine	PTGS1
Rhizoma coptidis	palmatine	PTGS2
Rhizoma coptidis	palmatine	RXRA
Rhizoma coptidis	quercetin	ACHE
Rhizoma coptidis	quercetin	ADRB2
Rhizoma coptidis	quercetin	AHR
Rhizoma coptidis	quercetin	AKR1B1
Rhizoma coptidis	quercetin	AKT1
Rhizoma coptidis	quercetin	ALOX5
Rhizoma coptidis	quercetin	AR
Rhizoma coptidis	quercetin	BAX
Rhizoma coptidis	quercetin	BCL2
Rhizoma coptidis	quercetin	BCL2L1
Rhizoma coptidis	quercetin	BIRC5
Rhizoma coptidis	quercetin	CASP3
Rhizoma coptidis	quercetin	CASP8
Rhizoma coptidis	quercetin	CASP9
Rhizoma coptidis	quercetin	CAV1
Rhizoma coptidis	quercetin	CCL2
Rhizoma coptidis	quercetin	CCND1
Rhizoma coptidis	quercetin	CD40LG
Rhizoma coptidis	quercetin	CDK1
Rhizoma coptidis	quercetin	CDKN1A
Rhizoma coptidis	quercetin	CHUK
Rhizoma coptidis	quercetin	COL1A1
Rhizoma coptidis	quercetin	COL3A1
Rhizoma coptidis	quercetin	CRP
Rhizoma coptidis	quercetin	CTSD
Rhizoma coptidis	quercetin	CXCL10
Rhizoma coptidis	quercetin	CXCL11

Rhizoma coptidis	quercetin	CXCL8
Rhizoma coptidis	quercetin	CYP1A2
Rhizoma coptidis	quercetin	CYP3A4
Rhizoma coptidis	quercetin	DPP4
Rhizoma coptidis	quercetin	EGF
Rhizoma coptidis	quercetin	EGFR
Rhizoma coptidis	quercetin	F10
Rhizoma coptidis	quercetin	F2
Rhizoma coptidis	quercetin	F3
Rhizoma coptidis	quercetin	F7
Rhizoma coptidis	quercetin	FOS
Rhizoma coptidis	quercetin	GJA1
Rhizoma coptidis	quercetin	HIF1A
Rhizoma coptidis	quercetin	HK2
Rhizoma coptidis	quercetin	HMOX1
Rhizoma coptidis	quercetin	HSF1
Rhizoma coptidis	quercetin	HSPA5
Rhizoma coptidis	quercetin	HSPB1
Rhizoma coptidis	quercetin	ICAM1
Rhizoma coptidis	quercetin	IFNG
Rhizoma coptidis	quercetin	IGF2
Rhizoma coptidis	quercetin	IGFBP3
Rhizoma coptidis	quercetin	IL10
Rhizoma coptidis	quercetin	IL1A
Rhizoma coptidis	quercetin	IL1B
Rhizoma coptidis	quercetin	IL2
Rhizoma coptidis	quercetin	IL6
Rhizoma coptidis	quercetin	INSR
Rhizoma coptidis	quercetin	IRF1
Rhizoma coptidis	quercetin	JUN
Rhizoma coptidis	quercetin	MAOB
Rhizoma coptidis	quercetin	MAPK1
Rhizoma coptidis	quercetin	MMP1
Rhizoma coptidis	quercetin	MMP2
Rhizoma coptidis	quercetin	MMP3
Rhizoma coptidis	quercetin	MMP9
Rhizoma coptidis	quercetin	MPO
Rhizoma coptidis	quercetin	MYC
Rhizoma coptidis	quercetin	NCF1
Rhizoma coptidis	quercetin	NFE2L2
Rhizoma coptidis	quercetin	NFKBIA
Rhizoma coptidis	quercetin	NOS3
Rhizoma coptidis	quercetin	NPEPPS
Rhizoma coptidis	quercetin	NQO1

Rhizoma coptidis	quercetin	PARP1
Rhizoma coptidis	quercetin	PIK3CG
Rhizoma coptidis	quercetin	PLAT
Rhizoma coptidis	quercetin	PLAU
Rhizoma coptidis	quercetin	PON1
Rhizoma coptidis	quercetin	PPARA
Rhizoma coptidis	quercetin	PPARD
Rhizoma coptidis	quercetin	PPARG
Rhizoma coptidis	quercetin	PRKACA
Rhizoma coptidis	quercetin	PRKCA
Rhizoma coptidis	quercetin	PRKCB
Rhizoma coptidis	quercetin	PTEN
Rhizoma coptidis	quercetin	PTGS1
Rhizoma coptidis	quercetin	PTGS2
Rhizoma coptidis	quercetin	RAF1
Rhizoma coptidis	quercetin	RASA1
Rhizoma coptidis	quercetin	RB1
Rhizoma coptidis	quercetin	RELA
Rhizoma coptidis	quercetin	RUNX2
Rhizoma coptidis	quercetin	RXRA
Rhizoma coptidis	quercetin	SELE
Rhizoma coptidis	quercetin	SERPINE1
Rhizoma coptidis	quercetin	SOD1
Rhizoma coptidis	quercetin	SPP1
Rhizoma coptidis	quercetin	STAT1
Rhizoma coptidis	quercetin	TGFB1
Rhizoma coptidis	quercetin	THBD
Rhizoma coptidis	quercetin	TNF
Rhizoma coptidis	quercetin	TOP1
Rhizoma coptidis	quercetin	TP53
Rhizoma coptidis	quercetin	VCAM1
Rhizoma coptidis	quercetin	VEGFA
Rhizoma coptidis	quercetin	XDH
Rhizoma coptidis	Worenine	AR
Rhizoma coptidis	Worenine	ESR1
Rhizoma coptidis	Worenine	NOS2
Rhizoma coptidis	Worenine	PTGS1
Rhizoma coptidis	Worenine	PTGS2
Semen Cuscutae	beta-sitosterol	ADRA1A
Semen Cuscutae	beta-sitosterol	ADRB2
Semen Cuscutae	beta-sitosterol	BAX
Semen Cuscutae	beta-sitosterol	BCL2
Semen Cuscutae	beta-sitosterol	CASP3
Semen Cuscutae	beta-sitosterol	CASP8

Semen Cuscutae	beta-sitosterol	CASP9
Semen Cuscutae	beta-sitosterol	CHRM1
Semen Cuscutae	beta-sitosterol	CHRM2
Semen Cuscutae	beta-sitosterol	CHRNA7
Semen Cuscutae	beta-sitosterol	DRD1
Semen Cuscutae	beta-sitosterol	HTR2A
Semen Cuscutae	beta-sitosterol	JUN
Semen Cuscutae	beta-sitosterol	MAP2
Semen Cuscutae	beta-sitosterol	OPRM1
Semen Cuscutae	beta-sitosterol	PDE3A
Semen Cuscutae	beta-sitosterol	PGR
Semen Cuscutae	beta-sitosterol	PIK3CG
Semen Cuscutae	beta-sitosterol	PON1
Semen Cuscutae	beta-sitosterol	PRKACA
Semen Cuscutae	beta-sitosterol	PRKCA
Semen Cuscutae	beta-sitosterol	PTGS1
Semen Cuscutae	beta-sitosterol	PTGS2
Semen Cuscutae	beta-sitosterol	SLC6A4
Semen Cuscutae	beta-sitosterol	TGFB1
Semen Cuscutae	campest-5-en-3beta-ol	PGR
Semen Cuscutae	CLR	NR3C2
Semen Cuscutae	CLR	PGR
Semen Cuscutae	Isofucosterol	ADH1C
Semen Cuscutae	Isofucosterol	NR3C2
Semen Cuscutae	Isofucosterol	PGR
Semen Cuscutae	isorhamnetin	ACHE
Semen Cuscutae	isorhamnetin	AKR1B1
Semen Cuscutae	isorhamnetin	AR
Semen Cuscutae	isorhamnetin	CALB2
Semen Cuscutae	isorhamnetin	CCNA2
Semen Cuscutae	isorhamnetin	CDK2
Semen Cuscutae	isorhamnetin	DPP4
Semen Cuscutae	isorhamnetin	ESR1
Semen Cuscutae	isorhamnetin	ESR2
Semen Cuscutae	isorhamnetin	F2
Semen Cuscutae	isorhamnetin	F7
Semen Cuscutae	isorhamnetin	GRIA2
Semen Cuscutae	isorhamnetin	GSK3B
Semen Cuscutae	isorhamnetin	MAOB
Semen Cuscutae	isorhamnetin	MAPK14
Semen Cuscutae	isorhamnetin	NCF1
Semen Cuscutae	isorhamnetin	NOS2
Semen Cuscutae	isorhamnetin	NOS3
Semen Cuscutae	isorhamnetin	OLR1

Semen Cuscutae	isorhamnetin	PIK3CG
Semen Cuscutae	isorhamnetin	PPARD
Semen Cuscutae	isorhamnetin	PPARG
Semen Cuscutae	isorhamnetin	PRKACA
Semen Cuscutae	isorhamnetin	PTGS1
Semen Cuscutae	isorhamnetin	PTGS2
Semen Cuscutae	isorhamnetin	PTPN1
Semen Cuscutae	isorhamnetin	RELA
Semen Cuscutae	isorhamnetin	XDH
Semen Cuscutae	kaempferol	ACHE
Semen Cuscutae	kaempferol	AHR
Semen Cuscutae	kaempferol	AKT1
Semen Cuscutae	kaempferol	ALOX5
Semen Cuscutae	kaempferol	AR
Semen Cuscutae	kaempferol	BAX
Semen Cuscutae	kaempferol	BCL2
Semen Cuscutae	kaempferol	CALB2
Semen Cuscutae	kaempferol	CASP3
Semen Cuscutae	kaempferol	CDK1
Semen Cuscutae	kaempferol	CHRM1
Semen Cuscutae	kaempferol	CHRM2
Semen Cuscutae	kaempferol	CYP1A2
Semen Cuscutae	kaempferol	CYP3A4
Semen Cuscutae	kaempferol	DPP4
Semen Cuscutae	kaempferol	F2
Semen Cuscutae	kaempferol	F7
Semen Cuscutae	kaempferol	HMOX1
Semen Cuscutae	kaempferol	ICAM1
Semen Cuscutae	kaempferol	INSR
Semen Cuscutae	kaempferol	JUN
Semen Cuscutae	kaempferol	MAPK8
Semen Cuscutae	kaempferol	MMP1
Semen Cuscutae	kaempferol	NOS2
Semen Cuscutae	kaempferol	NOS3
Semen Cuscutae	kaempferol	PGR
Semen Cuscutae	kaempferol	PIK3CG
Semen Cuscutae	kaempferol	PPARG
Semen Cuscutae	kaempferol	PRKACA
Semen Cuscutae	kaempferol	PTGS1
Semen Cuscutae	kaempferol	PTGS2
Semen Cuscutae	kaempferol	RELA
Semen Cuscutae	kaempferol	SELE
Semen Cuscutae	kaempferol	SLC6A2
Semen Cuscutae	kaempferol	SLPI

Semen Cuscutae	kaempferol	STAT1
Semen Cuscutae	kaempferol	TNF
Semen Cuscutae	kaempferol	VCAM1
Semen Cuscutae	kaempferol	XDH
Semen Cuscutae	matrine	CASP3
Semen Cuscutae	matrine	CD44
Semen Cuscutae	matrine	GRM2
Semen Cuscutae	matrine	HPSE
Semen Cuscutae	matrine	ICAM1
Semen Cuscutae	matrine	IL6
Semen Cuscutae	matrine	MMP2
Semen Cuscutae	matrine	MYC
Semen Cuscutae	matrine	RELA
Semen Cuscutae	matrine	TNF
Semen Cuscutae	NSC63551	GRM2
Semen Cuscutae	NSC63551	PGR
Semen Cuscutae	quercetin	ACHE
Semen Cuscutae	quercetin	ADRB2
Semen Cuscutae	quercetin	AHR
Semen Cuscutae	quercetin	AKR1B1
Semen Cuscutae	quercetin	AKT1
Semen Cuscutae	quercetin	ALOX5
Semen Cuscutae	quercetin	AR
Semen Cuscutae	quercetin	BAX
Semen Cuscutae	quercetin	BCL2
Semen Cuscutae	quercetin	BCL2L1
Semen Cuscutae	quercetin	BIRC5
Semen Cuscutae	quercetin	CASP3
Semen Cuscutae	quercetin	CASP8
Semen Cuscutae	quercetin	CASP9
Semen Cuscutae	quercetin	CAV1
Semen Cuscutae	quercetin	CCL2
Semen Cuscutae	quercetin	CCND1
Semen Cuscutae	quercetin	CD40LG
Semen Cuscutae	quercetin	CDK1
Semen Cuscutae	quercetin	CDKN1A
Semen Cuscutae	quercetin	CHUK
Semen Cuscutae	quercetin	COL1A1
Semen Cuscutae	quercetin	COL3A1
Semen Cuscutae	quercetin	CRP
Semen Cuscutae	quercetin	CTSD
Semen Cuscutae	quercetin	CXCL10
Semen Cuscutae	quercetin	CXCL11
Semen Cuscutae	quercetin	CXCL8

Semen Cuscutae	quercetin	CYP1A2
Semen Cuscutae	quercetin	CYP3A4
Semen Cuscutae	quercetin	DPP4
Semen Cuscutae	quercetin	EGF
Semen Cuscutae	quercetin	EGFR
Semen Cuscutae	quercetin	F10
Semen Cuscutae	quercetin	F2
Semen Cuscutae	quercetin	F3
Semen Cuscutae	quercetin	F7
Semen Cuscutae	quercetin	FOS
Semen Cuscutae	quercetin	GJA1
Semen Cuscutae	quercetin	HIF1A
Semen Cuscutae	quercetin	HK2
Semen Cuscutae	quercetin	HMOX1
Semen Cuscutae	quercetin	HSF1
Semen Cuscutae	quercetin	HSPA5
Semen Cuscutae	quercetin	HSPB1
Semen Cuscutae	quercetin	ICAM1
Semen Cuscutae	quercetin	IFNG
Semen Cuscutae	quercetin	IGF2
Semen Cuscutae	quercetin	IGFBP3
Semen Cuscutae	quercetin	IL10
Semen Cuscutae	quercetin	IL1A
Semen Cuscutae	quercetin	IL1B
Semen Cuscutae	quercetin	IL2
Semen Cuscutae	quercetin	IL6
Semen Cuscutae	quercetin	INSR
Semen Cuscutae	quercetin	IRF1
Semen Cuscutae	quercetin	JUN
Semen Cuscutae	quercetin	MAOB
Semen Cuscutae	quercetin	MAPK1
Semen Cuscutae	quercetin	MMP1
Semen Cuscutae	quercetin	MMP2
Semen Cuscutae	quercetin	MMP3
Semen Cuscutae	quercetin	MMP9
Semen Cuscutae	quercetin	MPO
Semen Cuscutae	quercetin	MYC
Semen Cuscutae	quercetin	NCF1
Semen Cuscutae	quercetin	NFE2L2
Semen Cuscutae	quercetin	NFKBIA
Semen Cuscutae	quercetin	NOS3
Semen Cuscutae	quercetin	NPEPPS
Semen Cuscutae	quercetin	NQO1
Semen Cuscutae	quercetin	PARP1

Semen Cuscutae	quercetin	PIK3CG
Semen Cuscutae	quercetin	PLAT
Semen Cuscutae	quercetin	PLAU
Semen Cuscutae	quercetin	PON1
Semen Cuscutae	quercetin	PPARA
Semen Cuscutae	quercetin	PPARD
Semen Cuscutae	quercetin	PPARG
Semen Cuscutae	quercetin	PRKACA
Semen Cuscutae	quercetin	PRKCA
Semen Cuscutae	quercetin	PRKCB
Semen Cuscutae	quercetin	PTEN
Semen Cuscutae	quercetin	PTGS1
Semen Cuscutae	quercetin	PTGS2
Semen Cuscutae	quercetin	RAF1
Semen Cuscutae	quercetin	RASA1
Semen Cuscutae	quercetin	RB1
Semen Cuscutae	quercetin	RELA
Semen Cuscutae	quercetin	RUNX2
Semen Cuscutae	quercetin	RXRA
Semen Cuscutae	quercetin	SELE
Semen Cuscutae	quercetin	SERPINE1
Semen Cuscutae	quercetin	SOD1
Semen Cuscutae	quercetin	SPP1
Semen Cuscutae	quercetin	STAT1
Semen Cuscutae	quercetin	TGFB1
Semen Cuscutae	quercetin	THBD
Semen Cuscutae	quercetin	TNF
Semen Cuscutae	quercetin	TOP1
Semen Cuscutae	quercetin	TP53
Semen Cuscutae	quercetin	VCAM1
Semen Cuscutae	quercetin	VEGFA
Semen Cuscutae	quercetin	XDH
Semen Cuscutae	sesamin	AUH
Semen Cuscutae	sesamin	CCND1
Semen Cuscutae	sesamin	ECE1
Semen Cuscutae	sesamin	F10
Semen Cuscutae	sesamin	G6PD
Semen Cuscutae	sesamin	IL10
Semen Cuscutae	sesamin	NOS3
Semen Cuscutae	sesamin	NOX1
Semen Cuscutae	sesamin	PTGS2
Semen Cuscutae	sesamin	SREBF1
