# Supplementary material for: Integrated Network Pharmacology and Proteomic Analyses of Targets and Mechanisms of Jianpi Tianjing Decoction in Treating Vascular Dementia
Source: Evid Based Complement Alternat Med. 2023 Jan 18;2023:9021546. doi: 10.1155/2023/9021546 (PMC9876684; doi:10.1155/2023/9021546)
Supplement: Supplementary Materials — Supplementary Table 1: Active JTD chemical compositions and targets. Supplementary Table 2: Morris water maze results. Supplementary Table 3: Differentially expressed proteins (DEPs) identification results. [file 9021546.f1.zip › Table 2 Morris water maze results.pdf]

Table 2: The results of the Morris water maze test

1 = the sham surgery group,<br>2 = the model group,<br>3 = the JTD group.	Escape latency (EL)					Times for crossing platform (TCP)	Time spent in platform quadrant (TSPQ)
**Group**	**Day1**	**Day2**	**Day3**	**Day4**	**Day5**	**TCP**	**TSPQ**
1	51.9	42.2	42.01	30.31	30.23	4	18.14
1	54.49	47.55	37.93	36.29	37.44	4	19.25
1	48.91	45.98	44.77	38.84	32.56	5	20.92
1	54.26	47.17	36.02	32.18	31.61	4	18.15
1	54.77	44.54	39.76	34.95	35.24	5	22.47
1	50.7	46.85	43.85	31.57	31.31	6	21.9
1	48.51	42.69	38.01	38.53	37.89	4	18.83
1	53.47	46.91	38.57	36.31	38.51	6	22
1	52.32	47.94	40	35.58	30.45	5	18
1	54.59	46.55	42.8	37.88	39	4	20.88
2	50	51.89	52.84	46.44	48.48	2	13.02
2	51.92	51.03	46.88	51.82	46.71	2	11.24
2	48.45	53.38	46.02	50.56	52.15	3	14
2	54.12	49.6	50.18	50.13	51.02	3	12.49
2	48.86	47.66	47.72	47.74	47.82	3	11.96
2	51.51	53.76	50.37	47.25	43.41	2	9.8
2	54.57	49.73	47.48	48.21	51.04	2	8.65
2	51.77	50.61	43.44	52.23	48.71	3	11.76
2	51	49.69	51.58	48.41	45.46	2	13.12
2	49.93	48.06	45.06	49.01	44.92	2	8.19
3	52.1	45.67	43.5	41.5	38.83	4	16.27
3	48.25	44.52	45.6	42.91	40.98	3	17.78
3	52.09	49.83	39.37	35.18	38.42	3	15.03
3	53.11	47.87	46.2	38.57	35.8	4	18.29
3	51.63	44.47	42.26	35.69	37.05	4	18.22
3	54.37	45.8	40.46	43.07	35.52	4	16.45
3	54.41	49.21	38.28	42.17	37.21	3	16.42
3	51.06	47.83	37.17	43.13	41.51	4	15.61
3	48.31	49.68	45.93	36.29	39.82	3	17.62
3	50.71	47.99	45.83	36.47	36.08	4	15.4
