# Supplementary material for: Integrated Network Pharmacology and Proteomic Analyses of Targets and Mechanisms of Jianpi Tianjing Decoction in Treating Vascular Dementia
Source: Evid Based Complement Alternat Med. 2023 Jan 18;2023:9021546. doi: 10.1155/2023/9021546 (PMC9876684; doi:10.1155/2023/9021546)
Supplement: Supplementary Materials — Supplementary Table 1: Active JTD chemical compositions and targets. Supplementary Table 2: Morris water maze results. Supplementary Table 3: Differentially expressed proteins (DEPs) identification results. [file 9021546.f1.zip › Table 3 Differentially expressed proteins (DEPs) identification results (1).pdf]

Table 3a: The DEPs identified between the JTD group and the model group

Protein IDs	Gene Name	Average the JTD group	Average the model group	the JTD vs model group	Regulation	P value
P01863	Ighg	132343.3333	38131.33333	3.470723989	UP	0.016385582
P17665	Cox7c	1284633.333	376400	3.412947219	UP	0.000913416
Q05186	Rcn1	104613.3333	46271	2.260883347	UP	0.025802175
Q5DTX6	Jcad	29283	13429.33333	2.180525218	UP	0.002342196
Q91X78	Erlin1	27469	15694	1.750286734	UP	0.049963387
Q0VBF8	Stum	870403.3333	529113.3333	1.64502249	UP	0.038695525
Q99KE1	Me2	152106.6667	94879.33333	1.6031591	UP	0.036296991
Q9JI18	Lrp1b	110650	71292.5	1.552056668	UP	0.029075597
P26645	Marcks	1416166.667	939666.6667	1.507094714	UP	0.003651826
Q8R0J7	Vps37b	63644.33333	42486	1.498007187	UP	0.036461174
P06728	Apoa4	267883.3333	180233.3333	1.486314037	UP	0.009395931
Q9D164	Fxyd6	281886.6667	203396.6667	1.385896196	UP	0.025114909
Q9WUT3	Rps6ka2	87813.33333	64578.66667	1.359788578	UP	0.011720176
Q61147	Cp	268386.6667	197776.6667	1.35701886	UP	0.0072588
Q99MU3	Adar	19901	14712.66667	1.35264398	UP	0.003078668
Q8K0B2	Lmbrd1	30695.5	22713.66667	1.351411045	UP	0.037025844
Q9D8N2	Dennd10	36794.66667	27984.66667	1.314815256	UP	0.049485747
A2A8L5	Ptprf	27773	21174.66667	1.311614508	UP	0.032527525
Q9DCL8	Ppp1r2	269423.3333	206430	1.305155904	UP	0.001420645
A2AT37	Upf2	27937.66667	21600.66667	1.293370575	UP	0.019563722
Q9ERL9	Gucy1a1	45384	35301.33333	1.285617163	UP	0.028833633
Q9D0M5	Dynll2	1759733.333	1370866.667	1.283664835	UP	0.011330793
Q99KR6	Rnf34	31276.5	24460.5	1.278653339	UP	0.014061367
Q6NXK8	Asic1	33660.33333	26377.66667	1.276092149	UP	0.022814252
Q9CZS1	Aldh1b1	147590	115873.3333	1.273718428	UP	0.028433841
Q8BTH8	Csnk1g1	47583	37358.66667	1.27368036	UP	0.005233829
P23953	Ces1c	138003.3333	108430	1.272741246	UP	0.018796443
Q69ZS8	Kazn	230533.3333	181150	1.272610176	UP	0.046017386
P06837	Gap43	2794766.667	2198100	1.271446552	UP	0.048984185
P48428	Tbca	145856.6667	114833.3333	1.270159652	UP	0.011980203
Q3SXD3	Hddc2	93323.33333	73856	1.263584994	UP	0.002955351
Q91ZP9	Necab2	246193.3333	195040	1.262270987	UP	0.022744045
Q7TPH6	Mycbp2	205086.6667	163030	1.257968881	UP	0.022336459
Q6PAJ1	Bcr	88254.33333	71228	1.239039891	UP	0.042445536
P99024	Tubb5	9714500	7842033.333	1.238773107	UP	0.045380491
P63248	Pkia	52737	42822	1.231539863	UP	0.049607268
Q8JZP2	Syn3	144513.3333	117626.6667	1.228576287	UP	0.035217203
Q60738	Slc30a1	64048.66667	52189.33333	1.227236728	UP	0.017528601
Q9R087	Gpc6	11782.5	9704.366667	1.214144148	UP	0.028299623
Q91WM1	Strbp	35149.66667	42210.33333	0.832726584	DOWN	0.044084601
Q6GSS7	Hist2h2aa1	5095733.333	6150466.667	0.828511658	DOWN	0.041332783
Q9WVQ5	Apip	29318.5	35851	0.817787509	DOWN	0.037062859
P26043	Rdx	138693.3333	170473.3333	0.813577881	DOWN	0.039977514
Q14BI2	Grm2	104525.3333	128600	0.812794194	DOWN	0.040657166

Q9JL26	Fmnl1	49723	61363.33333	0.810304742	DOWN	0.001129598
Q9CQ48	Nudcd2	65056	80338	0.809778685	DOWN	0.006926828
P97384	Anxa11	107699	133050	0.809462608	DOWN	0.043743624
Q3TJZ6	Fam98a	44135.33333	54530	0.809377101	DOWN	0.023772184
Q91XL9	Osbpl1a	260843.3333	323073.3333	0.807381193	DOWN	0.039300799
Q00896	Serpina1c	750670	942486.6667	0.796478111	DOWN	0.04804475
Q8BR63	Fam177a1	39364.66667	49777.66667	0.790809801	DOWN	0.032845925
P55194	Sh3bp1	36711.33333	46508.66667	0.789343922	DOWN	0.004216103
Q8BYI8	Fam234b	49148	62287.33333	0.789052884	DOWN	0.02290384
P97315	Csrp1	544986.6667	690740	0.788989586	DOWN	0.021136537
Q80U19	Daam2	229880	295516.6667	0.777891828	DOWN	0.004583158
Q9D662	Sec23b	14781.33333	19254	0.767701949	DOWN	0.028442709
P61514	Rpl37a	57034	74987	0.760585168	DOWN	0.008864422
P63250	Kcnj3	32577.5	44691.66667	0.728939027	DOWN	0.016594292
E9PZ19	Igsf9b	17222	23940	0.719381788	DOWN	0.008403547
Q14B80	Kcnc2	36385.66667	53014.66667	0.686332084	DOWN	0.043072916
P46414	Cdkn1b	33239.33333	49406.66667	0.672770206	DOWN	0.012927636
Q60805	Mertk	23379	35038.33333	0.667240641	DOWN	0.015026922
Q9QYE9	Plekhb1	22807.66667	34800.33333	0.655386443	DOWN	0.002243339
Q9Z2N8	Actl6a	11289.93333	17588	0.641911152	DOWN	0.012447812
Q64676	Ugt8	14458.9	32360.33333	0.446809365	DOWN	0.039381391

Table 3b: The DEPs identified between the model group and the sham surgery group

Protein IDs	Gene Name	Average the sham surgery group	Average the model group	the model vs sham surgery group	Regulation	P value
Q6P2L7	Golm2	50908	27543.66667	1.848265179	UP	0.020914124
P13634	Ca1	95238.66667	52896	1.80048901	UP	0.026576698
P04919	Slc4a1	61184.33333	37641.66667	1.625441665	UP	0.020582396
Q61838	Pzp	235716.6667	152566.6667	1.545007647	UP	0.005895653
P17047	Lamp2	82843.66667	55580.66667	1.490512288	UP	0.043780094
Q9CPU0	Glo1	3557666.667	2403800	1.48001775	UP	0.035635137
Q00896	Serpina1c	942486.6667	643953.3333	1.46359467	UP	0.032266489
P62309	Snrpg	177493.3333	125846.6667	1.410393601	UP	0.026091916
P29699	Ahsg	168653.3333	122230.3333	1.379799341	UP	0.022846159
P02088	Hbb-b1	18817333.33	13818666.67	1.361732922	UP	0.020685453
Q9DCU2	Pllp	338260	249680	1.354774111	UP	0.027165794
P28665	Mug1	176593.3333	132870	1.329068513	UP	0.043560678
Q9JKV1	Adrm1	276473.3333	208033.3333	1.328985739	UP	0.040462237
Q14B80	Kcnc2	53014.66667	40292.66667	1.315739837	UP	0.03357753
P01027	C3	285593.3333	221146.6667	1.291420475	UP	0.04480379
Q9QWR8	Naga	39726	31085.33333	1.277966029	UP	0.03112367
Q6GSS7	Hist2h2aa1	6150466.667	4814700	1.277435077	UP	0.032421347
P16045	Lgals1	342620	270160	1.26821143	UP	0.024244442
Q921I1	Tf	571790	450913.3333	1.268070731	UP	0.029919048
Q00623	Apoa1	382443.3333	304690	1.255188333	UP	0.031602081

P58064	Mrps6	31966	25563	1.250479208	UP	0.024334854
Q8BYI8	Fam234b	62287.33333	50171.33333	1.241492486	UP	0.023126409
Q6EDY6	Carmil1	29139.5	23505	1.239714954	UP	0.004750879
Q9D338	Mrpl19	36687.33333	29693	1.235554957	UP	0.009313414
Q91WE1	Snx15	34813	28457.33333	1.223340205	UP	0.021080711
Q0GA42	Cnnm1	12036	9896.45	1.216193686	UP	0.042563402
O35459	Ech1	85625	70559	1.213523434	UP	0.043802569
O89116	Vti1a	97606.33333	80691.33333	1.209625982	UP	0.023323933
Q8BGN8	Synpr	367573.3333	305006.6667	1.205132128	UP	0.036962992
Q9DAW9	Cnn3	103084.6667	85721	1.202560244	UP	0.031655548
Q9QYI5	Dnajb2	190253.3333	158350	1.201473529	UP	0.039189928
Q3UIU2	Ndufb6	432110	360063.3333	1.200094428	UP	0.038408686
Q80V91	Dtx3	50558.66667	60908.66667	0.830073443	DOWN	0.049714591
Q8C5W3	Tbcel	28324.33333	34143	0.829579514	DOWN	0.014101843
P10107	Anxa1	15029	18390	0.817237629	DOWN	0.011616974
P62897	Cycs	1660600	2032200	0.817143982	DOWN	0.010049636
Q9ER73	Elp4	23840.66667	29575	0.806108763	DOWN	0.01704677
Q80ZI6	Lrsam1	27544	34294.33333	0.80316476	DOWN	0.018927402
Q8BTY2	Slc4a7	76863.33333	95872.66667	0.801723119	DOWN	0.010957983
Q8R086	Suox	18049.66667	22743.33333	0.793624505	DOWN	0.029927018
Q99MU3	Adar	14712.66667	18952.5	0.776291606	DOWN	0.023834243
Q6Y7W8	Gigyf2	38396.33333	50029	0.767481527	DOWN	0.009293656
Q9QZN4	Fbxo6	31119.66667	40754.33333	0.763591602	DOWN	0.033625207
Q91YX5	Lpgat1	24631.66667	32329	0.761906235	DOWN	0.009423113
P62996	Tra2b	185990	244930	0.759359817	DOWN	0.002744073
Q8CGA4	Mturn	23371	30860.66667	0.757307036	DOWN	0.02465369
Q9CXR1	Dhrs7	37178	49644.66667	0.748882055	DOWN	0.009806626
Q8VBX6	Mpdz	19712.5	26633	0.740153193	DOWN	0.026324581
P23591	Gfus	53031.66667	72945	0.727008934	DOWN	0.046808621
Q9D0I4	Stx17	26639	36850	0.722903664	DOWN	0.045064805
Q8C7H1	Mmaa	44901.66667	62393.66667	0.719651033	DOWN	0.044531151
Q61503	Nt5e	16684	25714	0.648829431	DOWN	0.038043105
P01863	Ighg	38131.33333	157560	0.242011509	DOWN	0.044859537

Table 3c: The DEPs identified between the JTD group and the sham surgery group

Protein IDs	Gene Name	Average the JTD group	Average the sham surgery group	the JTD vs sham surgery group	Regulation	P value
P06909	Cfh	37624	20364.5	1.847528788	UP	0.037729271
Q99JR5	Tinagl1	33597	20737	1.620147562	UP	0.027206909
Q64519	Sdc3	35726.66667	22502	1.587710722	UP	0.022555767
P26645	Marcks	1416166.667	923533.3333	1.533422363	UP	0.008978324
O08677	Kng1	64320.66667	42320.33333	1.519852553	UP	0.049257189
P06728	Apoa4	267883.3333	179196.6667	1.49491248	UP	0.044845644
Q8R0J7	Vps37b	63644.33333	44084.66667	1.443684123	UP	0.031899975
Q9JHW4	Eefsec	26577	18504.66667	1.436232302	UP	0.032487023
P23953	Ces1c	138003.3333	96652	1.427837327	UP	0.029223656

P04919	Slc4a1	52710	37641.66667	1.40030994	UP	0.022405975
Q8JZV7	Amdhd2	14996.5	10816.5	1.386446632	UP	0.021467724
P13634	Ca1	73321	52896	1.386135057	UP	0.012149454
Q9EPV8	Ubl5	42892.5	31128	1.377939476	UP	0.047397986
Q69ZX8	Ablim3	11946.66667	8870.7	1.346755799	UP	0.012897954
Q9D0M5	Dynll2	1759733.333	1311966.667	1.34129424	UP	0.006849092
Q00623	Apoa1	391110	304690	1.283632545	UP	0.024663608
Q91X96	Rabif	131593.3333	102546.3333	1.283257324	UP	0.038273496
Q8BTS4	Nup54	216556.6667	169566.6667	1.277118144	UP	0.020441676
O35459	Ech1	88601.33333	70559	1.255705627	UP	0.04564748
Q9QX47	Son	45528.33333	36314.33333	1.253729014	UP	0.012688733
Q9CR60	Golt1b	35447	28643.33333	1.237530548	UP	0.025563514
Q9DAW6	Prpf4	24545	19919.66667	1.232199334	UP	0.031846927
Q3U2A8	Vars2	32603	26865	1.213586451	UP	0.044326315
Q7TNV0	Dek	54990.33333	66077	0.83221595	DOWN	0.017792533
Q99K23	Ufsp2	22529	27195.33333	0.828414189	DOWN	0.029963251
Q9EPC1	Parva	40672.66667	49529.33333	0.821183406	DOWN	0.004661963
P55288	Cdh11	42736	52051.66667	0.821030386	DOWN	0.021362301
Q3TCN2	Plbd2	54343	71410.66667	0.760992756	DOWN	0.015246981
Q9JIX0	Eny2	48700.66667	64680	0.752947846	DOWN	0.037399722
Q922F4	Tubb6	821573.3333	1102390	0.74526559	DOWN	0.024280068
Q8BUY8	Gprasp2	22233.33333	30666.5	0.72500394	DOWN	0.024727935
Q8VBX6	Mpdz	19129	26633	0.718244283	DOWN	0.004984888
Q6P8I4	Pcnp	77923.66667	117415	0.663660236	DOWN	0.01457305
Q8CHG3	Gcc2	94143	372916.6667	0.252450503	DOWN	0.03244557
